# *In Vitro* and *in Silico* Analysis of Phytochemicals From *Fallopia dentatoalata* as Dual Functional Cholinesterase Inhibitors for the Treatment of Alzheimer’s Disease

**DOI:** 10.3389/fphar.2022.905708

**Published:** 2022-07-11

**Authors:** Yichuang Wu, Xiangdong Su, Jielang Lu, Meifang Wu, Seo Young Yang, Yang Mai, Wenbin Deng, Yongbo Xue

**Affiliations:** ^1^ School of Pharmaceutical Sciences (Shenzhen), Sun Yat-sen University, Shenzhen, China; ^2^ Department of Pharmaceutical Engineering, Sangji University, Wonju, South Korea

**Keywords:** *Fallopia dentatoalata*, Alzheimer’s disease, cholinesterase (AChE, BChE), polyphenols, phenylpropanoid sucrose esters, kinetic—spectrophotometric method, molecular docking

## Abstract

Current studies have found that butyrylcholinesterase (BuChE) replaces the biological function of acetylcholinesterase (AChE) in the late stage of Alzheimer’s disease. Species in the genus of Fallopia, rich in polyphenols with diverse chemical structures and significant biological activities, are considered as an important resource for screening natural products to against AD. In this study, thirty-four compounds (1–34) were isolated from Fallopia dentatoalata (Fr. Schm.) Holub, and their inhibitory effects against AChE and BuChE were assessed. Compounds of the phenylpropanoid sucrose ester class emerged as the most promising members of the group, with 31–33 displaying moderate AChE inhibition (IC50 values ranging from 30.6 ± 4.7 to 56.0 ± 2.4 µM) and 30–34 showing potential inhibitory effects against BuChE (IC50 values ranging from 2.7 ± 1.7 to 17.1 ± 3.4 µM). Tacrine was used as a positive control (IC50: 126.7 ± 1.1 in AChE and 5.5 ± 1.7 nM in BuChE). Kinetic analysis highlighted compounds 31 and 32 as non-competitive inhibitors of AChE with Ki values of ∼30.0 and ∼34.4 µM, whilst 30–34 were revealed to competitively inhibit BuChE with Ki values ranging from ∼1.8 to ∼17.5 µM. Molecular binding studies demonstrated that 30–34 bound to the catalytic sites of BuChE with negative binding energies. The strong agreement between both *in vitro* and in silico studies highlights the phenylpropanoid sucrose esters 30–34 as promising candidates for use in future anti-cholinesterase therapeutics against Alzheimer’s disease.

## Introduction

Alzheimer’s disease (AD) is a chronic and occult neurodegenerative disease featured by permanent memory loss and progressive cognitive impairments, which is relatively prevalent in the elder population (Wang and Zhang, 2019). The progression of AD believes to involve with multiple complicated pathogenesis and etiologies, and the cholinergic hypothesis is the earliest and most widely studied pathogenetic mechanism of AD (Du et al., 2018; Wang and Zhang, 2019). According to the cholinergic hypothesis, acetylcholine (ACh) is an essential neurotransmitter which acts as a chemical messenger when secreted by nerve cells (Du et al., 2018). This signaling is important for numerous biological processes, including the support of cognitive functions and memory in the central nervous system (CNS), and activating muscles in the peripheral nervous system (PNS) (Krátký et al., 2018). The cholinesterases, including acetylcholinesterase (AChE, EC 3.1.1.7) and butyrylcholinesterase (BuChE, EC 3.1.1.8), are a group of esterases that catalyze the hydrolysis of cholinergic neurotransmitters into choline and their corresponding acids, which results in the return of the activated cholinergic neuron back to its resting state (Nicolet et al., 2003). AChE is regarded as a high-performance cholinesterase with highly specific catalytic activity towards ACh (80%), while BuChE, a substitute for AChE, is a non-selective cholinesterase, which can degrade both ACh and butyrylcholine (Nicolet et al., 2003; Dvir et al., 2010). Previous studies have demonstrated that ACh deficiency can cause several neurological disorders in the cortical cholinergic sites of the brain among patients with AD (Martinez and Castro, 2006). Several pharmacotherapeutics that act as acetylcholinesterase inhibitors (AChEIs) have been demonstrated to increase the concentration of ACh and thereby contribute to the alleviation the symptoms of AD (Martinez and Castro, 2006). Although AChEIs hitherto still remain the most prevailing drugs for the treatment of AD, those drugs demonstrate the limited clinical outcomes and fail to prevent the disease progression (Wang and Zhang, 2019). Research into the pathological mechanisms of the disease has revealed that AChE levels accumulate at a much higher rate than those of BChE during the early stages of AD, whereas towards the later stages of the disease, the levels of BChE increase and ultimately substitute the function of AChE (Mesulam et al., 2002; Martinez and Castro, 2006). Whilst a number of AChE inhibitors that originate from natural sources have been developed into therapeutic agents for the treatment of AD, the number of BChE inhibitory therapeutics from the natural pool is substantially lower (Clive et al., 2005). The phytochemical study outlined herein aims to address this deficiency through the characterization and biological evaluation of the natural constituents isolated from *F. dentatoalata*.

*F. dentatoalata* is an annual plant with wing-shaped flowers which belongs to the genus *Fallopia*. It is naturally distributed throughout several East Asian countries, including China, Japan, and India. *F. dentatoalata* is a popular cultivated species in China, especially in the Jiangsu, Hubei, and Jilin provinces (Meng et al., 2021a). In recent decades, a substantial number of phytochemical studies have led to the isolation of various chemical constituents from *F. dentatoalata*, including anthraquinones, stilbenes, amides, and flavonoids (Meng et al., 2021a). *Fallopia multiflora* is the most popular *Fallopia* species and its roots enjoy use as a traditional Chinese medicine, mainly to boost immunity and prevent aging (Lin et al., 2015). Moreover, recent studies have demonstrated its preventive and curative effects in the treatment of neurodegenerative diseases (Qian et al., 2020). Some evidence indicates that the chemical constituents of *F. dentatoalata* share a similar profile to those of *F. multiflora*. Emodin (**21**) and tetrahydroxystilbene​-​2-​*O*-​β-​D-​glucoside (**26**), for example, are two major compounds isolated from *F. dentatoalata* and *F. multiflora* which exhibit a wide range of neurobiological properties including neuroprotection and anti-neuroinflammation (Fakhri et al., 2021; Semwal et al., 2021). In adidtion, extracts from *F*. *multiflora* have been reported to show inhibitory activity against AChE (IC_50_: 9.11 μg/ml) and BuChE (IC_50_: 4.83 μg/ml) (Li et al., 2017). By contrast, the chemical extracts from *F. dentatoalata* have yet to have their inhibitory activities agaisnt AChE and BuChE assessed.

Our ongoing program to discover natural anti-cholinergic candidates from *Fallopia* species has resulted the isolation of thirty-four compounds from the aerial parts of *F. Dentatoalata*, which are disclosed for the first time herein*.* The chemical structures of these isolates were elucidated based on extensive methods of characterization, including 1D- and 2D-NMR, HR-FAB-MS spectroscopic analyses, and by comparison with previously reported data. Enzymatic assays were conducted to evaluate their inhibitory activities against AChE and BuChE.

## Materials and Methods

### General Experimental Procedures

Column chromatographic procedures were performed using silica gel (80–120 mesh and 200–300 mesh, Qingdao Marine Chemical Co. Ltd., Qingdao, China) and Sephadex™ LH-20 gel (40–70 μm; Merck, Darmstadt, Germany), whereas precoated silica gel (GF254, Qingdao Marine Chemical Co. Ltd., Qingdao) plates were used for TLC analyses. Spots were visualized by heating silica gel plates sprayed with 10% H_2_SO_4_ in EtOH. UV spectra were recorded using a Waters UV-2401A spectrophotometer equipped with a DAD and a cell of 1 cm pathlength. Methanolic samples were scanned from 190 to 400 nm in 1 nm steps. Semipreparative HPLC was performed on an Agilent 1120 apparatus equipped with a UV detector and a reversed-phase C_18_ column (5 μm, 10 × 250 mm, Welch Ultimate XB-C18). 1D (^1^H, ^13^C) spectra of all compounds were recorded on Bruker AM-600, AM-500, and AM-400 NMR spectrometers (Bruker, Karlsruhe, Germany), with TMS as the internal reference. |Enzymatic activity experiments were performed using SpectraMax i3x (Molecular Devices, Austria).

### Chemicals and Reagents

All HPLC solvents were purchased from Guangdong Guanghua Sci-Tech Co. Ltd. (Guangzhou, China). Acetylcholinesterase (C3389), acetylthiocholine iodide (A5751), butyrylcholinesterase (C1057), butyrylthiocholine iodide (B3253), and 5,5-dithiobis (2-nitrobenzoic acid) (DTNB), tacrine and dimethylsulfoxide (DMSO) were purchased from Sigma-Aldrich Co. (St. Louis, Missouri, United States). All chemicals and solvents used in column chromatography and assays were acquired from commercial sources.

### Plant Material

The dried aerial parts of *F*. *dentatoalata* were collected from Nanyang City of Henan Province in China and taxonomically identified by Prof. Zulin Ning (Key Laboratory of Plant Resources Conservation and Sustainable Utilization, Chinese Academy of Sciences). A voucher specimen (SYSUSZ-2019-X3) was deposited at the Department of Natural Medicines, School of Pharmaceutical Sciences (Shenzhen), Sun Yat-sen University.

### Extraction and Isolation

The air-dried aerial parts of *F*. *dentatoalata* (9.3 kg) were extracted using 70% aqueous ethanol (15 L × 4 × 2 h at room temperature) with ultrasonic assistance. The combined extracts were filtered and evaporated under reduced pressure to yield a brown residue (1.6 kg). The residue was suspended in H_2_O and successively partitioned with petroleum ether (PE) (10 L), ethyl acetate (EA) (10 L), and *n*-butanol (10 L), yielding petroleum ether (121.3 g), ethyl acetate (93.0 g) and *n*-BuOH (311.3 g) extracts.

The ethyl acetate layer was subjected to silica gel (100–200 mesh) column chromatography (CC) (CH_2_Cl_2_−MeOH, 100:1–1:1 v/v) to obtain seven fractions (Fr. 1–7). Fr. 2 (10.0 g) was decolorized using MCI gel CC (MeOH–H_2_O, 20–100%, v/v) to afford three subfractions (Fr. 2A−2C). Fr. 2B was subjected to silica gel CC (PE−EA, 20:1–5:1, v/v) to yield compound **21** (18.0 mg). Compound **22** (15.0 mg) was purified from Fr. 2C by silica gel CC (PE-EA, 20:1–5:1, v/v). Fr. 3 (8.3 g) was decolorized using MCI gel CC (MeOH–H_2_O, 20–100%, v/v) to afford three subfractions (Fr. 3A−3C). Fr. 3A was successively separated *via* Sephadex LH-20 CC (CH_2_Cl_2_−MeOH, 1:1, v/v) and silica gel CC (CH_2_Cl_2_−MeOH, 50:1–10:1, v/v) to afford compound **5** (5.0 mg) and **17** (10.0 mg). Compounds **1** (15.0 mg), **3** (8.0 mg), and **16** (13.0 mg) were purified from Fr. 3B by silica gel CC (CH_2_Cl_2_-MeOH, 50:1–10:1, v/v). Fr. 4 (7.0 g) was subjected to silica gel CC (CH_2_Cl_2_−MeOH, 50:1–10:1, v/v) to afford four subfractions (Fr. 4A−4D). Fr. 4D was successively separated via MCI CC (MeOH–H_2_O, 20–100%, v/v) and Sephadex LH-20 CC (CH_2_Cl_2_−MeOH, 1:1, v/v) to afford compounds **28** (16.0 mg) and **29** (26.0 mg). Fr. 5 (17.5 g) was subjected to silica gel CC (CH_2_Cl_2_−MeOH, 50:1–1:1, v/v) to afford six subfractions (Fr. 5A−5F). Fr. 5D was successively separated *via* Sephadex LH-20 CC (CH_2_Cl_2_−MeOH, 1:1, v/v) and silica gel CC (CH_2_Cl_2_−MeOH, 50:1–30:1, v/v) to afford **7** (26.0 mg). Fr. 5E was successively separated *via* silica gel CC (CH_2_Cl_2_−MeOH, 30:1–2:1, v/v), Sephadex LH-20 CC (CH_2_Cl_2_−MeOH, 1:1, v/v), and semi-preparative HPLC (MeOH−H_2_O, 30:70 to 80:20, v/v, 3 ml/min) to afford **18** (10.0 mg, *t*_R_ 6.3 min) and **19** (2.0 mg, *t*_R_ 6.6 min). Fr. 5F was successively separated *via* Sephadex LH-20 CC (CH_2_Cl_2_−MeOH, 1:1, v/v) and semi-preparative RP-HPLC (MeCN–H_2_O, 20:80–60: 40, v/v, 3 ml/min) to afford **30** (4.0 mg, *t*_R_ 7.1 min), **31** (7.0 mg, *t*_R_ 8.1 min), and **32** (5.0 mg, *t*_R_ 7.3 min). Fr. 6 (35.0 g) was subjected to silica gel CC (CH_2_Cl_2_−MeOH, 50:1–1:1, v/v) to afford six subfractions (Fr. 6A−6F). Fr. 6C was successively separated *via* Sephadex LH-20 CC (CH_2_Cl_2_−MeOH, 1:1, v/v) and silica gel CC (CH_2_Cl_2_−MeOH, 30:1–5:1, v/v) to afford compounds **26** (16.0 mg) and **27** (8.0 mg). Fr. 6D was successively separated *via* silica gel CC (CH_2_Cl_2_−MeOH, 30:1–2:1, v/v), Sephadex LH-20 CC (CH_2_Cl_2_−MeOH, 1:1, v/v), and semi-preparative RP-HPLC (MeOH−H_2_O, 40:60 to 80:20, v/v, 3 ml/min) to afford compounds **4** (10.0 mg, *t*_R_ 8.9 min), **8** (5.0 mg, *t*_R_ 5.7 min), **9** (15.0 mg, *t*_R_ 11.7 min), **11** (2.0 mg, *t*_R_ 10.1 min), and **24** (15.0 mg, *t*_R_ 13.6 min). Similarly, compounds **10** (12.0 mg, *t*_R_ 15.1 min), **12** (74.0 mg, *t*_R_ 11.5 min), **13** (24.0 mg, *t*_R_ 12.7 min), **20** (6.0 mg, *t*_R_ 16.6 min), **33** (6.0 mg, *t*_R_ 17.4 min), and **34** (7.0 mg, *t*_R_ 19.6 min) were obtained from Fr. 6E and Fr. 6F by semi-preparative RP-HPLC (MeOH–H_2_O, 20:80–80:20, v/v, 3 ml/min). Fr. 7 (7.0 g) was successively separated *via* silica gel CC (CH_2_Cl_2_−MeOH, 20:1–0:1, v/v) and semipreparative RP-HPLC (MeOH–H_2_O, 20:80–100:0, v/v, 3 ml/min) to afford two subfractions (Fr. 7A−7B). Fr. 7B was successively separated *via* Sephadex LH-20 CC (CH_2_Cl_2_−MeOH, 1:1, v/v), silica gel CC (CH_2_Cl_2_−MeOH, 20:1–1:1, v/v), and semipreparative RP-HPLC (MeOH–H_2_O, 50:50–100:0, v/v, 3 ml/min) to afford **2** (10.0 mg, *t*_R_ 4.6 min), **6** (7.0 mg, *t*_R_ 6.3 min), **14** (10.0 mg, *t*_R_ 7.6 min), **15** (10.0 mg, *t*_R_ 8.7 min), **23** (6.0 mg, *t*_R_ 12.6 min), and **25** (10.0 mg, *t*_R_ 9.1 min).

### Lapathoside B (30)

Yellowish amorphous solid. UV (MeOH): *λ*_max_ (log ε) 217 (2.70), 323 (3.20) nm, see Supplementary Figure S6. ^1^H (600 MHz, CD_3_OD-*d*_*4*_) and ^13^C-NMR (151 MHz, CD_3_OD-*d*_*4*_) data, see Supplementary Table S1; HR-FAB-MS *m/z* 1039.2842 [M+Na]^+^ (calcd for C_51_H_52_NaO_22_^+^ 1039.2848), see Supplementary Figure S1.

### Vanicoside B (31)

Yellowish amorphous solid. UV (MeOH): *λ*_max_ (log ε) 213 (3.00), 318 (5.10) nm, see Supplementary Figure S7. ^1^H (600 MHz, CD_3_OD-*d*_*4*_) and ^13^C NMR (151 MHz, CD_3_OD-*d*_*4*_) data, see Supplementary Table S1; HR-FAB-MS *m/z* 1011.2840 [M+Na+CH_3_OH]^+^ (calcd for C_50_H_52_NaO_21_^+^ 1011.2899), see Supplementary Figure S2.

### Lapathoside A (32)

Yellowish amorphous solid. UV (MeOH): *λ*_max_ (log ε) 215 (4.70), 322 (5.80) nm, see Supplementary Figure S8. ^1^H (600 MHz, CD_3_OD-*d*_*4*_) and ^13^C NMR (151 MHz, CD_3_OD-*d*_*4*_) data, see Supplementary Table S1; HR-FAB-MS *m/z* 1004.3183 [M+NH_4_]^+^ (calcd for C_50_H_54_NO_21_^+^ 1004.3188), see Supplementary Figure S3.

### Smilaside J (33)

Yellowish amorphous solid. UV (MeOH): *λ*_max_ (log ε) 2218 (4.70), 235.7 (3.20), 332 (5.80) nm, see Supplementary Figure S9. ^1^H (400 MHz, CD_3_OD-*d*_*4*_) and ^13^C NMR (101 MHz, CD_3_OD-*d*_*4*_) data, see Supplementary Table S2; HR-FAB-MS *m/z* 858.2823 [M+NH_4_]^+^ (calcd for C_41_H_48_NO_19_^+^ 858.2821), see Supplementary Figure S4.

### Smilaside G (34)

Yellowish amorphous solid. UV (MeOH): *λ*_max_ (log ε) 229 (2.70), 314 (4.70) nm, see Supplementary Figure S10. ^1^H (600 MHz, CD_3_OD-*d*_*4*_) and ^13^C NMR (150 MHz, CD_3_OD-*d*_*4*_) data, see Supplementary Table S2; HR-FAB-MS *m/z* 833.2271 [M+Na]^+^ (calcd for C_40_H_42_NaO_18_^+^ 833.2263), see Supplementary Figure S5.

### AChE and BuChE Assays

*In vitro* cholinesterase assays were performed using a modified version of previously published methods (Kim et al., 2016). Briefly, 130 µL of enzyme (acetylcholinesterase and butyrylcholinesterase: 0.05 Unit/mL) in 50 mM potassium phosphate buffer (pH 7.4) was mixed with 20 µL of compounds (1–0.002 mM) dissolved in methanol in a 96-well plate. 25 µL of 1 mM DTNB [5,5′-dithiobis (2-nitrobenzoic acide)] and 25 µL of 5 mM substrate, acetylthiocholine iodide (A5751), and butyrylthiocholine iodide (B3253) were sequentially added to wells in the plate. Plates were incubated at 37°C for 30 min then monitored with a Microplate reader (SpectraMax i3x) (405 nm). Tacrine was used as positive control. The inhibition ratio was calculated using the equation:

Inhibitory activity(%)=[(ΔC-ΔI)/ΔC]×100

Where C and I are the intensity of control and inhibitor after 20 min, respectively.

### Molecular Docking Simulation

Autodock package 4.2 (La Jolla, CA. United States) was used for the molecular docking of receptor with ligand. Ligands were built as 3D structures and minimized with MM2 charge using Chem3D Pro 17.1. For flexible ligands, single bonds were assigned using AutoDockTools. The 3D structures of BuChE (pdb ID: 1P0I) was derived using the RCSB protein data bank. Hydrogens were added to both, then each were assigned with computed gasteiger charges. To simulate docking, a grid containing the active site was set (grid points X.Y.Z 80.80.100 for AChE, and X.Y.Z 60.80.70 for BuChE) with 0.375 Å spacing. Docking simulations of protein structures and newly built ligands were performed using the Lamarckian Genetic Algorithm. Finally, ligands were docked into the box 25,000,000 times, then the results of the top 50 ranks were extracted. Data were presented in figures using Discovery Studio and Ligpot (Cambridge, United Kingdom) and Chimera (San Francisco, CA, United States).

### Statistical Analysis

All inhibitory concentration data was obtained from independent experiments carried out in triplicate. Results are shown as the mean ± standard error of the mean (SEM). The results were subjected to analysis using Sigma plot 14.5 (Systat Software Inc., San Jose, CA, United States).

## Results and Discussion

### Isolation and Identification

**FIGURE 1:**
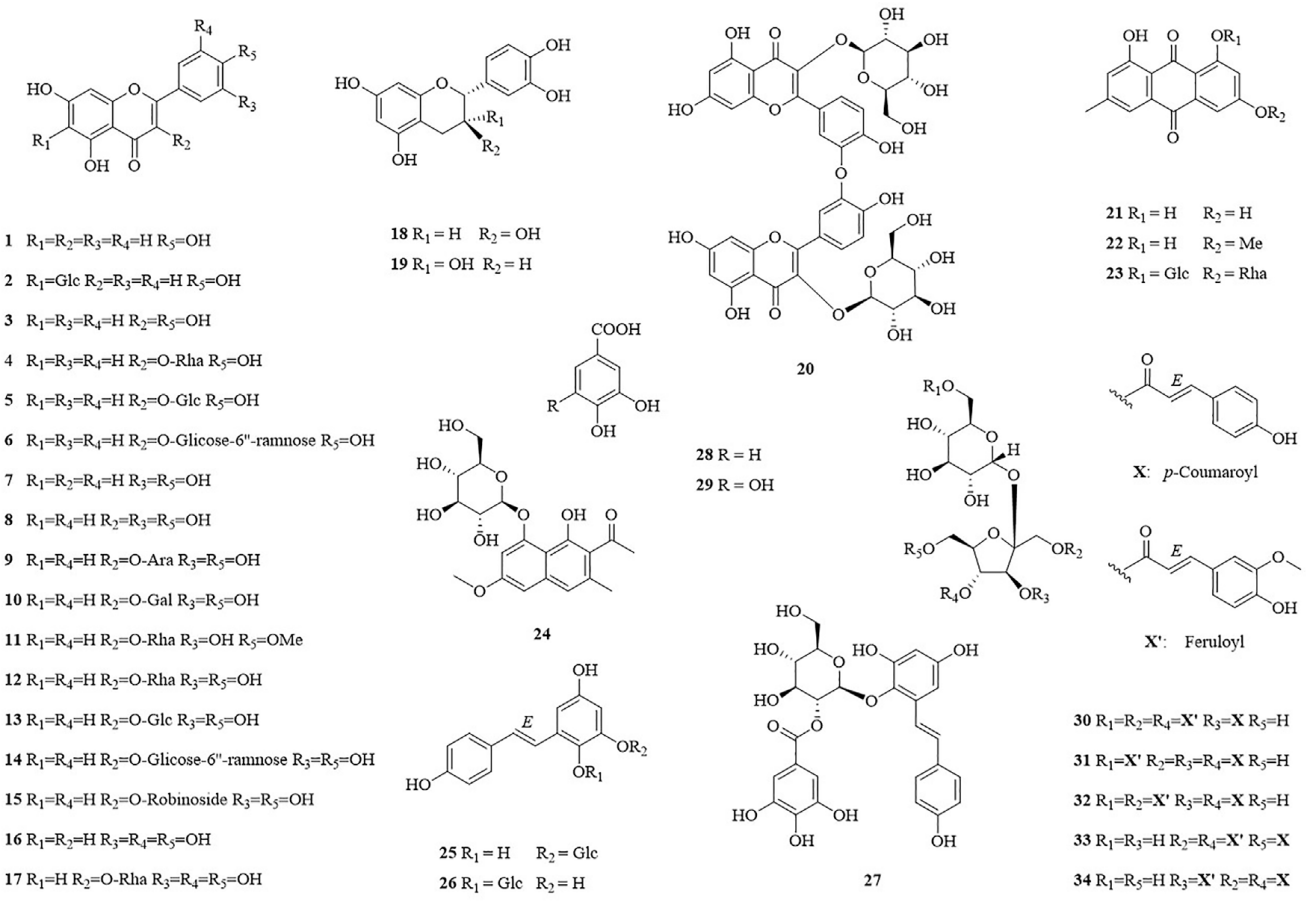
Structure of isolated compounds **1**–**34** from *F. dentatoalata*.

In this study, thirty-four compounds (**1**–**34**) were isolated from the aerial parts of *F. dentatoalata via* extensive column chromatography. These were identified as apigenin (**1**) (Ha et al., 2012), isovitexin (**2**) (Jayasinghe et al., 2004), kaempferol (**3**) (Fukai and Nomura, 1988), afzelin (**4**) (Xu et al., 2009), astragalin (**5**) (Otsuka et al., 1989), kaempferol-3-​rutinoside (**6**) (de Sa de Sousa Nogueira et al., 2013), luteolin (**7**) (Zhang et al., 2015), quercetin (**8**) (Lesjak et al., 2018), guaijaverin (**9**) (Yoshida et al., 1992), hyperoside (**10**) (Isaza et al., 2001), tamarixetin-​3-​rhamnoside (**11**) (Norman et al., 2021), quercitrin (**12**) (Chen et al., 2008), isoquercitrin (**13**) (Jin et al., 2009), rutin (**14**) (Kazuma et al., 2003), quercetin-​3-​*O*-​robinoside (**15**) (Dossou et al., 2021), myricetin (**16**) (Zhang et al., 2004), myricitrin (**17**) (Kil et al., 2019), (+)-​catechin (**18**) (Foo et al., 1997), (-)-​epicatechin (**19**) (Lin and Lee, 2010), zizyflavoside B (**20**) (Yang et al., 2020), emodin (**21**) (Meselhy, 2003), physcion (**22**) (Jo et al., 2011), glucofrangulin A (**23**) (Bezabih and Abegaz, 1998), torachrysone-​8-​*O*-​β-​D-​glucoside (**24**) (Zhao et al., 2017), polydatin (**25**) (Yi et al., 2020), tetrahydroxystilbene​-​2-​*O*-​β-​D-​glucoside (**26**) (Tsai et al., 2018), (*E*)-2,3,5,4′-tetrahydroxystilbene-2-*O*-(2″-*O*-galloyl)-β-D-glucoside (**27**) (Nguyen et al., 2020), protocatechuic acid (**28**) (Meng et al., 2021b), gallic acid (**29**) (Chen et al., 2021), lapathoside B (**30**) (Takasaki et al., 2001), vanicoside B (**31**) (Kumagai et al., 2005), lapathoside A (**32**) (Takasaki et al., 2001), smilaside J (**33**) (Zhang et al., 2008), and smilaside G (**34**) (Takasaki et al., 2001). Identification was enabled by comparison of their spectroscopic data with those reported previously, see Supplementary Table S3–S13 (Figure 1).

### Enzyme Assays

**FIGURE 2:**
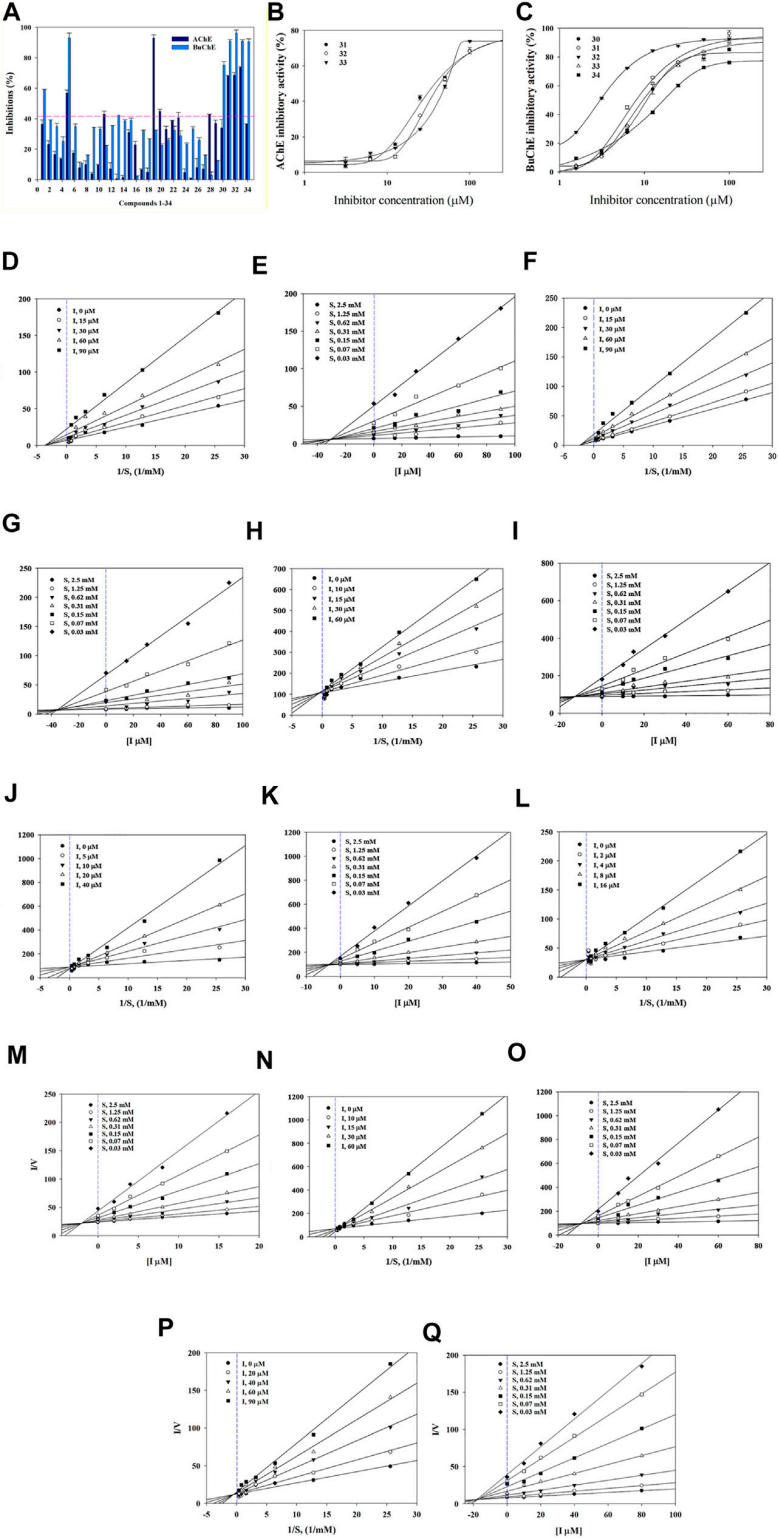
Inhibitory activity of compounds **1**–**34** at 100 µM towards AChE and BuChE **(A)**. IC_50_ values of **31**–**33** on AChE **(B)** and **30**–**34** BuChE **(C)**. Lineweaver-Burk plots **(D,F)** and Dixon plots **(E,G)** of compounds **31** and **32** on AChE. Lineweaver-Burk plots **(H, J, L, N, P)** and Dixon plots **(I, K, M, O, Q)** of compounds **30**–**34** on BuChE.

Compounds were first evaluated *in vitro* for their inhibitory activities against AChE and BuChE at a concentration of 100 µM. The amount of 5-thio-2-nitrobenzoic acid produced from the substrates (acetylthiocholine iodide and butyrylthiocholine iodide) was quantified in the presence or absence of compounds using a SpectraMax i3x model at 405 nm. Tacrine was used as a positive control (IC_50_: 126.7 ± 1.1 in AChE and 5.5 ± 1.7 nM in BuChE). Compounds **1**–**34** exhibited inhibitory effects on AChE and BuChE with different ratios, ranging from 0.9 ± 0.3% to 73.8 ± 0.4%, and from 2.3 ± 0.3% to 96.3 ± 2.0% of the control value at 100 μM, respectively (Figure 2A). Among them, compounds 31–33 exhibited more than 60% inhibition in a dose-dependent manner on AChE, with IC_50_ values of 32.3 ± 4.7, 30.6 ± 4.7, and 56.0 ± 2.4 µM, respectively (Figure 2B; Table 1). Compounds 30–33 displayed potent inhibitory activities against BuChE with IC_50_ values of 2.7 ± 1.7 and 10.9 ± 4.9 µM, while compound 34 exhibited a moderate inhibition on BuChE with an IC_50_ value of 17.1 ± 3.4 µM (Figure 2C; Table 1).

**TABLE 1:** Inhibitory activity of compounds **30**–**34** against AChE and BuChE.

Comp	AChE^a^			BuChE^a^		
	IC_50_ (µM)	Inhibition type	*K*_*i*_ (µM)	IC_50_ (µM)	Inhibition type	*K*_*i*_ (µM)
30	>100	‒	‒	10.9 ± 4.9	Competitive	∼12.1
31	32.3 ± 4.7	Non-Competitive	∼30.0	7.5 ± 4.1	Competitive	∼3.5
32	30.6 ± 4.7	Non-Competitive	∼34.4	2.7 ± 1.7	Competitive	∼1.8
33	56.0 ± 2.4	‒	‒	10.1 ± 4.6	Competitive	∼8.5
34	>100	‒	‒	17.1 ± 3.4	Competitive	∼17.5
Tacrine^b^	126.7 ± 1.1 nM	‒	‒	5.5 ± 1.7 nM	‒	‒

a All compounds were examined in triplicate.

b Positive control.

(‒) Not tested.

Structure-activity relationships (SAR) of compounds **30**–**34** were guided by their IC_50_ values in order to better understand their respective pharmacophores (Supplementary Figure S11). Compound **33** (IC_50_:10.1 µM) with a *p*-coumaroyl motif linked to the C-4 position of fructose, demonstrated an inhibitory effect comparable with compound **30** (IC_50_:10.9 µM), while compound **31** (IC_50_: 7.5 µM), which has a feruloyl group linked to the C-6′ position of glucose and a *p*-coumaroyl linked to the C-3 position of fructose, exhibited an inhibitory activity over 2-fold higher than that of Compound **34** (IC_50_:17.1 µM). In addition, the presence of a feruloyl group at C-6′, a *p*-coumaroyl group at C-6, and a feruloyl group linked to the C-1 position of fructose such as those found in compound **32** (IC_50_: 2.7 µM), are likely to be the structural units most responsible for the observed anti-cholinesterase activities. Consequently, the inhibitory activities decreased in the order **32** > **31** > **33** > **30** > **34**, with compound **32** being the most potent of the series.

### Enzyme Kinetics Study

The binding mechanisms of isolated compounds which displayed IC_50_ values less than 50 µM against cholinesterase were investigated. This was achieved by performing enzyme kinetic studies on AChE/BuChE in the presence of different concentrations of compounds **30**–**34** (2–90 µM) at various steady-state substrate concentrations (0.07–5 mM). The interactions between compounds and cholinesterase are represented using classic double-reciprocal Lineweaver-Burk and Dixon plots (Lineweaver and Burk, 1934; Dixon, 1953). Compounds **31** and **32** were revealed to have various V_max_ values and a K_m_ value, which confirmed both were non-competitive inhibitors that docked with both AChE and substrate-bound AChE (Figures 2D,F) (Lee et al., 2018). The resulting linear equations for compounds **30**–**34** with BuChE led to a series of lines with different slopes that crossed at similar intercepts on the vertical axis and different points on the horizontal axis. Compounds **30**–**34** were therefore designated as competitive inhibitors of BuChE (Figures 2H,J,L,N,P) (Kim et al., 2016). Furthermore, the intersections of the lines on the Dixon plots indicated that the inhibition constants (*K*_i_) of compounds **31** and **32** towards AChE were ∼30.0 and ∼34.4 μM, respectively (Figures 2E,G; Table 1). The *K*_i_ values of compounds **30**–**34** for the inhibition of BuChE were ∼12.1, ∼3.5, ∼1.8, ∼8.5 and 17.5 μM, respectively (Figures 2I,K,M,O,Q; Table 1).

### Molecular Docking of BuChE Inhibition

**FIGURE 3:**
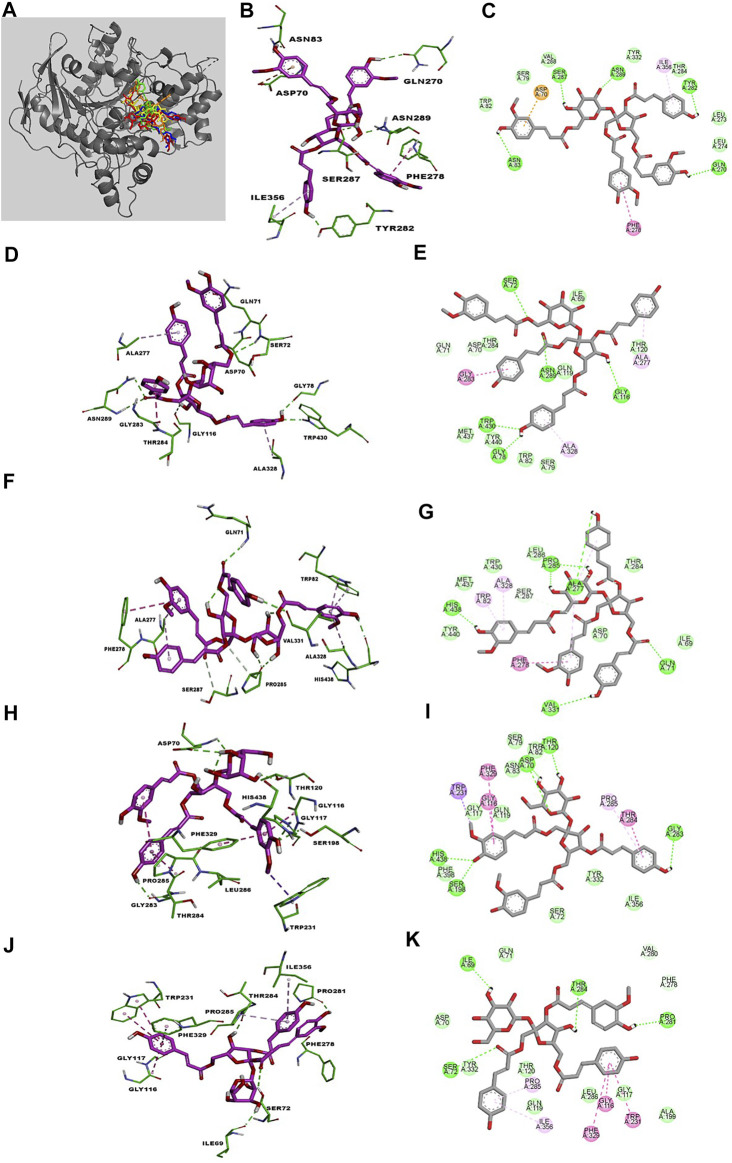
The best poses of compounds **30**–**34** (**30**, red; **31**, green; **32**, orange; **33**, yellow; **34**, blue) docked with BuChE **(A)**. Molecular docking models **(B, D, F, H, J)** and 2D ligand interaction diagrams **(C, E, G, I, K)** of BuChE inhibition at the catalytic pocket by compounds **30**–**34**, respectively. Different interactions between compounds and amino acid residues in the catalytic site are designated by the following: thick light purple stick models represent compounds **30**–**34**, green dotted lines represent hydrogen bonds, light green lines represent Van der Waals interactions, dark pink lines represent π–π and π–σ interactions, and light pink lines represent π–alkyl interactions.

Kinetic studies demonstrated that compounds **30**–**34** significantly downregulated the catalytic activity of BuChE by competitively binding to its active site. Previous researches demonstrated that the active site gorge of BuChE include: catalytic triad (Ser198-His438-Glu325), acyl loop (Ala277-Leu286-Val288), π-cation site (Tyr82-Ala328), ꞷ-loop (Ile69-Ser79), oxyanion hole (Ala199-Gly116-Gly117), and peripheral site (Asn68-Glu70-Tyr332) (Nicolet et al., 2003; Vyas et al., 2010). Molecular docking simulations were subsequently performed in order to confirm and identify their binding energies and outline the binding interactions (i.e. hydrogen-bonding, Van der Waals, and hydrophobic interactions) between ligands **30**–**34** and BuChE (Table 2). Full docking views of each phenylpropanoid sucrose ester bound to the catalytic site of BuChE are shown in Figure 3.

**TABLE 2:** Binding site residues and docking scores of compounds **30**–**34** bound to BuChE obtained using Autodock 4.2.

Comp	Binding energy (kcal/mol)	Hydrogen bond interaction	Van der Waals	Hydrophobic interactions			Other interactions	
				π-π stacked	π-σ	π-alkyl	π-anion/cation	π-amide
**30**	–7.13	Asn83, Ser287, Asn289, Tyr282, Gln270	Val288, Trp82, Ser79, Tyr332, Leu273, Thr284, Leu274	Phe278		Ile356	Asp70	
**31**	–7.36	Gly78, Trp430, Ser72, Asn289, Gly116	Gln71, Asp70, Thr284, Ile69, Thr120, Met437, Tyr440, Trp82, Ser79			Ala328, Ala277		Gly283
**32**	–7.55	Ala277, His438, Pro285, Val331, Gln71	Ile69, Tyr440, Met437, Trp430, Leu286, Asp70, Thr284	Phe278		Ala328, Trp82, Ala277		
**33**	–5.33	His438, Ser198, Asp70, Thr120, Gly283	Phe398, Ile356, Gly117, Gln119, Asn83, Ser79, Trp82, Tyr332, Ser72	Phe329	Trp231	Pro285		Thr284
								Gly116
**34**	–5.23	Ile69, Ser72, Thr284, Pro281	Asp70, Tyr332, Gln119, Thr120, Leu286, Gln71, Gly117, Phe278	Phe329		Pro285		Gly116
				Trp231		Ile356		

Compound **30** achieved and Autodock score of –7.13 kcal/mol (Table 2). Hydroxyl and carboxyl groups displayed hydrogen bonding with the key amino acids Asn83 (2.72 Å), Ser287 (2.66 Å), Asn289 (2.61 Å), Tyr282 (2.83 Å), and Gln270 (3.19 Å) (Figures 3B,C). An aromatic ring of the feruloyl group attached to C-1 of fructose exhibited π-π stacking with Phe278 at a distance of 4.59 Å. Other residues from different active sites, including Val288, Trp82, Ser79, Tyr332, Leu273, Leu274, Thr284, Ile356, and Asp70, showed bonding with **30** *via* Van der Waals, π-alkyl and π-anion interactions (Figures 3B,C).

Compounds **31** and **32** displayed stable binding energies of –7.36 and –7.55 kcal/mol when bound to the active site, which arose as a result of interactions with five (Gly78 at 2.09 Å, Trp430 at 2.31 Å, Ser72 at 2.90 Å, Asn289 at 2.38 and 2.68 Å, Gly116 2.78 Å) and six (Ala277 at 2.02 Å, His438 at 2.03 Å, Pro285 at 2.01 and 2.50 Å, Val331 at 2.48 Å, Gln71 at 2.41 Å) hydrogen bonds to amino acid residues in the BuChE active site, respectively (Figures 3D,E; Table 2). Both **31** and **32** shared the same catalytic residues of Tyr440, Met437, Asp70, Ile69 (*via* Van der Waals interactions) and Ala328 and Ala277 (*via* π-alkyl interactions) (Figures 3D–G; Table 2). Furthermore, compound **31** exhibited additional Van der Waals interactions with Gln71, Thr284, Thr120, Trp82, Ser79, and a π-amide interaction with Gly283, which further confirmed its interaction with BuChE (Figures 3D,E; Table 2). The remaining residues of Trp430, Leu286, Thr284, and Trp82 from the active site formed Van der Waals and π-alkyl interactions with compound **32** (Figures 3F,G; Table 2).

Compounds **33** and **34** bound to the catalytic region of BuChE with a relatively large binding energies of –5.33 and –5.23 kcal/mol respectively, which suggests that the number of phenylpropanoids affects the binding energy. As shown in Figures 3H–K, compounds **33** and **34** formed five hydrogen bonds (His438 at 2.35 Å, Ser198 at 2.16 Å, Asp70 at 2.36 and 2.58 Å, Thr120 at 2.02 Å, Gly283 at 2.12 Å), meanwhile **34** formed four (Thr284 at 1.74, Pro281 at 2.02 Å, Ile69 at 2.11 Å and Ser72 at 3.04 Å), respectively (Table 2). Additionally, **33** and **34** shared the same residues of Tyr332, Gly117, and Gln119 (*via* Van der Waals interactions), Phe329 and Trp231 (*via* π-π and π-σ interactions), Pro285 and Gly116 (*via* π-alkyl and π-amide interactions), respectively (Table 2). The other residue interactions of **33** and **34** are similar with those of compounds **30**–**32** (Figure 3; Table 2)**.** The molecular docking results are consistent with the those from the kinetic studies, which confirm compounds **30**–**34** to be competitive inhibitors binding at the catalytic active site of BuChE. Furthermore, molecular docking simulations between these isolates and BuChE enzyme suggest that phenylpropanoid sucrose esters represent a novel molecular architecture in the development of BuChE inhibitors.

## Conclusion

Thirty-four previously reported compounds were isolated from the MeOH extracts of the aerial parts of *F. dentatoalata*. These compounds were further classified into seventeen flavonoids and their glycosides (**1**–**17**), two flavan-3-ols (**18**–**19**), a biflavone (**20**), three anthraquinones (**21**–**23**), a naphtolic glycoside (**24**), three stilbenoid derivatives (**25**–**27**), two phenolic acids (**28**–**29**), and five phenylpropanoid sucrose esters (**30**–**34**). The inhibitory activities of all compounds against AChE and BuChE were evaluated. To the best of our knowledge, this is the first phytochemical investigation on potential anti-cholinesterase candidates from *F. dentatoalata*. However, several compounds already have been evaluated for their potential anti-cholinesterase effects before, especially flavonoids (Wu et al., 2017; Borowiec et al., 2022). For examples, compounds **1**, **5** and **19** showed over 50% inhibitions against AChE and BuChE, which further proved to be comparable with reported values (Kim et al., 2016; Nugroho et al., 2018; Islam et al., 2021; Karatas et al., 2022). In addition, compounds **31**–**33** showed moderate inhibition of AChE, and were determined to be non-competitive inhibitors. Compounds **30**–**34** significantly suppressed the activity of BuChE and were identified as competitive inhibitors. *In vitro* and *in silico* results indicate that the phenylpropanoid sucrose esters **30**–**34** from *F. dentatoalata* hold potential as candidates for future development as anti-cholinesterase therapeutics against AD.

## Data Availability

The original contributions presented in the study are included in the article/Supplementary Material, further inquiries can be directed to the corresponding authors.
