# Supplementary figures and images for: In Vitro and in Silico Analysis of Phytochemicals From Fallopia dentatoalata as Dual Functional Cholinesterase Inhibitors for the Treatment of Alzheimer’s Disease
Source: Front Pharmacol. 2022 Jul 11;13:905708. doi: 10.3389/fphar.2022.905708 (PMC9313597; doi:10.3389/fphar.2022.905708)

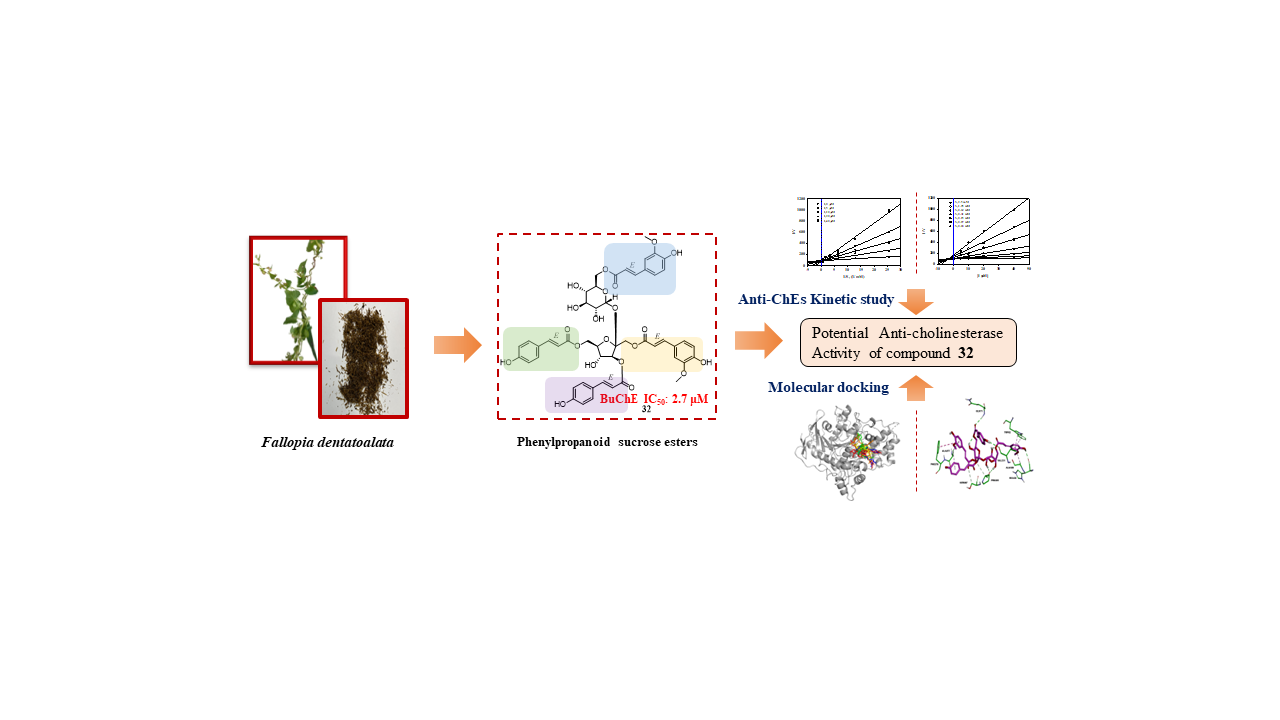

Supplement: Supplementary file 1 [file Image1.tif]
