# Supplementary material for: In Vitro and in Silico Analysis of Phytochemicals From Fallopia dentatoalata as Dual Functional Cholinesterase Inhibitors for the Treatment of Alzheimer’s Disease
Source: Front Pharmacol. 2022 Jul 11;13:905708. doi: 10.3389/fphar.2022.905708 (PMC9313597; doi:10.3389/fphar.2022.905708)

Supplementary Material

***In vitro* and *in silico* analysis of phytochemicals from Fallopia dentatoalata as dual functional cholinesterase inhibitors for the treatment of Alzheimer’s disease**

Yichuang Wu ^1, †^, Xiangdong Su ^1, †^, Jielang Lu ^1^, Meifang Wu ^1^, Seo Young Yang ^2,^ Yang Mai ^1^, Wenbin Deng ^1,^*, Yongbo Xue ^1,^*

^1^ School of Pharmaceutical Sciences (Shenzhen), Sun Yat-sen University, Shenzhen 518107, China

^2^ Department of Pharmaceutical Engineering, Sangji University, Wonju 26339, Republic of Korea

*** Correspondence:**

Yongbo Xue

[xueyb@mail.sysu.edu.cn](mailto:xueyb@mail.sysu.edu.cn)

Wenbin Deng

[dengwb5@mail.sysu.edu.cn](mailto:dengwb5@mail.sysu.edu.cn)

^†^There authors have contributed equally to this work and shared first authorship

**List of Supporting Information**

Content Page

[Figure S1. HR-FAB-MS spectrum of compound](#_Toc85485185) **[30](#_Toc85485185)** [4](#_Toc85485185)

[Figure S2. HR-FAB-MS spectrum of compound **31** 4](#_Toc85485186)

[Figure S3. HR-FAB-MS spectrum of compound **32** 5](#_Toc85485187)

[Figure S4. HR-FAB-MS spectrum of compound **33** 5](#_Toc85485188)

[Figure S5. HR-FAB-MS spectrum of compound **35** 6](#_Toc85485189)

[Figure S6. UV spectrum of compound **30** 6](#_Toc85485190)

[Figure S7. UV spectrum of compound **31** 6](#_Toc85485191)

[Figure S8. UV spectrum of compound **32** 7](#_Toc85485192)

[Figure S9. UV spectrum of compound **33** 7](#_Toc85485193)

[Figure S10. UV spectrum of compound **34** 7](#_Toc85485194)

[Figure S11. Graphical depiction of the structure-activity relationships (SARs) of compounds **30**–**34** 8](#_Toc85485195)

[Table S1. ^1^H (600 MHz, CD_3_OD-*d_4_*) and ^13^C NMR (151 MHz, CD_3_OD-*d_4_*) Data for **30**–**32**. 9](#_Toc85485196)

[Table S2. ^1^H (400 MHz, CD_3_OD-*d_4_*) and ^13^C NMR (101 MHz, CD_3_OD-*d_4_*) Data for **33**, 1H (600 MHz, CD_3_OD-*d_4_*) and 13C NMR (151 MHz, CD_3_OD-*d_4_*) Data for **34**. 11](#_Toc85485197)

[Table S3. ^1^H (600 MHz, DMSO-*d*_6_) and ^13^C NMR (151 MHz, DMSO-*d*_6_) Data for **1** and **3**, ^1^H (400 MHz, DMSO-*d*_6_) and ^13^C NMR (101 MHz, DMSO-*d*_6_) Data for**2**. 12](#_Toc85485197)

[Table S4. ^1^H (600 MHz, CD_3_OD-*d_4_*) and ^13^C NMR (151 MHz, CD_3_OD-*d_4_*) Data for **4**–**6** 13](#_Toc85485197)

[Table S5. ^1^H (600 MHz, DMSO-*d*_6_) and ^13^C NMR (151 MHz, DMSO-*d*_6_) Data for **7** and **8**, ^1^H (600 MHz, CD_3_OD-*d_4_*) and ^13^C NMR (151 MHz, CD_3_OD-*d_4_*) Data for **9** 14](#_Toc85485197)

[Table S6. ^1^H (600 MHz, DMSO-*d*_6_) and ^13^C NMR (151 MHz, DMSO-*d*_6_) Data for **10**, ^1^H (600 MHz, CD_3_OD-*d_4_*) and ^13^C NMR (151 MHz, CD_3_OD-*d_4_*) Data for **11** and **12** 15](#_Toc85485197)

[Table S7. ^1^H (600 MHz, DMSO-*d*_6_) and ^13^C NMR (151 MHz, DMSO-*d*_6_) Data for **13**, ^1^H (600 MHz, CD_3_OD-*d_4_*) and ^13^C NMR (151 MHz, CD_3_OD-*d_4_*) Data for **14**, ^1^H (400 MHz, DMSO-*d*_6_) and ^13^C NMR (101 MHz, DMSO-*d*_6_) Data for **15** 16](#_Toc85485197)

[Table S8. ^1^H (600 MHz, DMSO-*d*_6_) and ^13^C NMR (151 MHz, DMSO-*d*_6_) Data for **16** and **17**, ^1^H (400 MHz, CD_3_OD-*d_4_*) and ^13^C NMR (101 MHz, CD_3_OD-*d_4_*) Data for **18**, ^1^H (600 MHz, CD_3_OD-*d_4_*) and ^13^C NMR (151 MHz, CD_3_OD-*d_4_*) Data for **19** 17](#_Toc85485197)

[Table S9. ^1^H (600 MHz, CD_3_OD-*d_4_*) and ^13^C NMR (151 MHz, CD_3_OD-*d_4_*) Data for **20** 18](#_Toc85485197)

[Table S10. ^1^H (400 MHz, DMSO-*d*_6_) and ^13^C NMR (101 MHz, DMSO-*d*_6_) Data for **21** and **22**, ^1^H (600 MHz, CD_3_OD-*d_4_*) and ^13^C NMR (151 MHz, CD_3_OD-*d_4_*) Data for **23** 18](#_Toc85485197)

[Table S11. ^1^H (600 MHz, CD_3_OD-*d_4_*) and ^13^C NMR (151 MHz, CD_3_OD-*d_4_*) Data for **24** 19](#_Toc85485197)

[Table S12. ^1^H (600 MHz, CD_3_OD-*d_4_*) and ^13^C NMR (151 MHz, CD_3_OD-*d_4_*) Data for **25** and **26,** ^1^H (400 MHz, CD_3_OD-*d_4_*) and ^13^C NMR (101 MHz, CD_3_OD-*d_4_*) Data for **27** 20](#_Toc85485197)

[Table S13. ^1^H (400 MHz, CD_3_OD-*d_4_*) and ^13^C NMR (101 MHz, CD_3_OD-*d_4_*) Data for **28** and **29** 21](#_Toc85485197)

[Figure S12-S36. Purity Anylysis by HPLC of Compounds **1**‒**13**, **16**, **18**, **20**‒**22**, **24**, **26**, **31**‒**34** 21](#_Toc85485195)

Figure S27-S95. ^1^H NMR and ^13^C NMR Spectrum (400 and 600 MHz) of  [Compounds **1**‒**34** 29](#_Toc85485195)


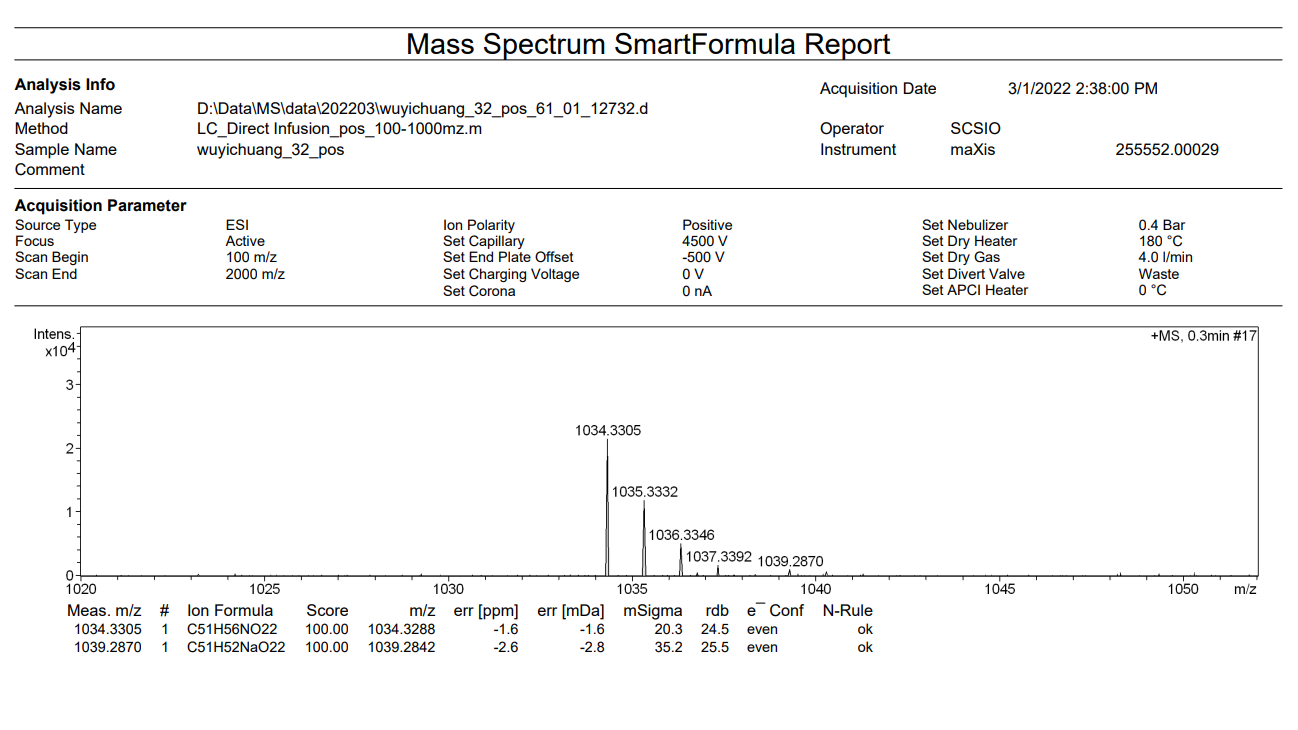


**Figure S1**. HR-FAB-MS spectrum of compound **30**

**
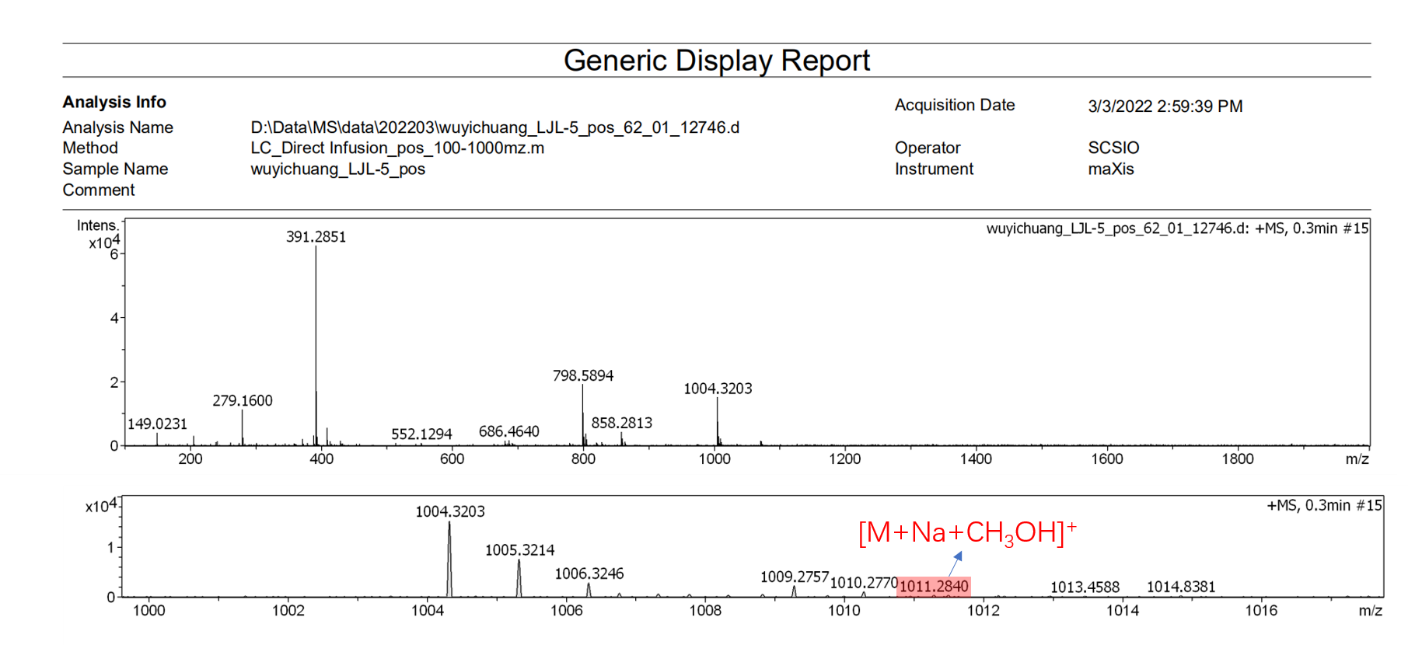
**

**Figure S2**. HR-FAB-MS spectrum of compound **31**


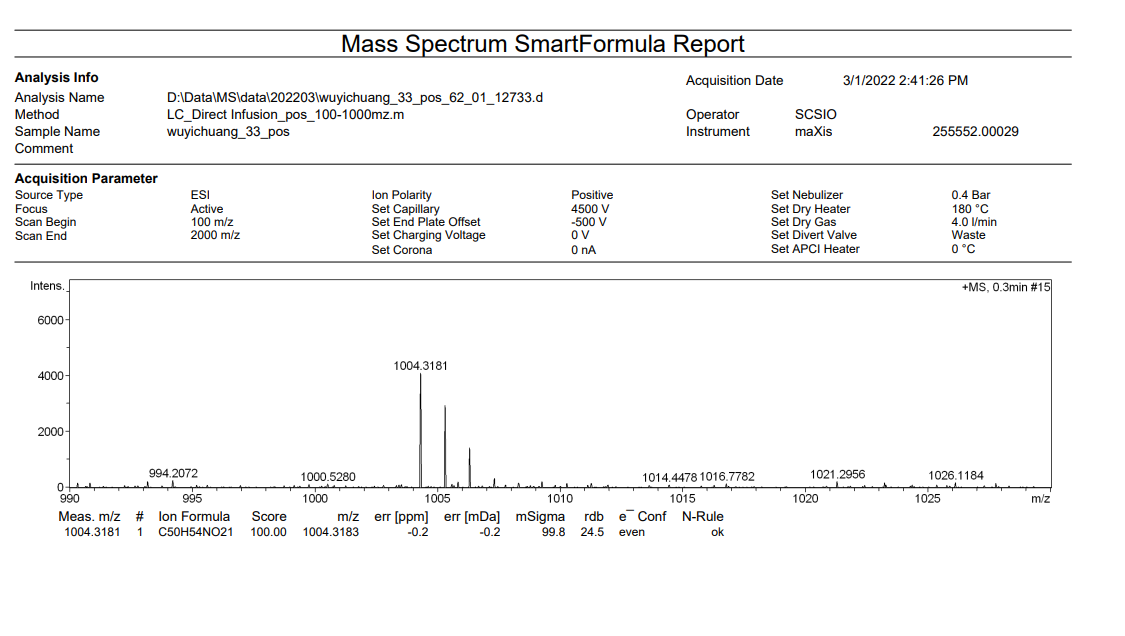


**Figure S3**. HR-FAB-MS spectrum of compound **32**


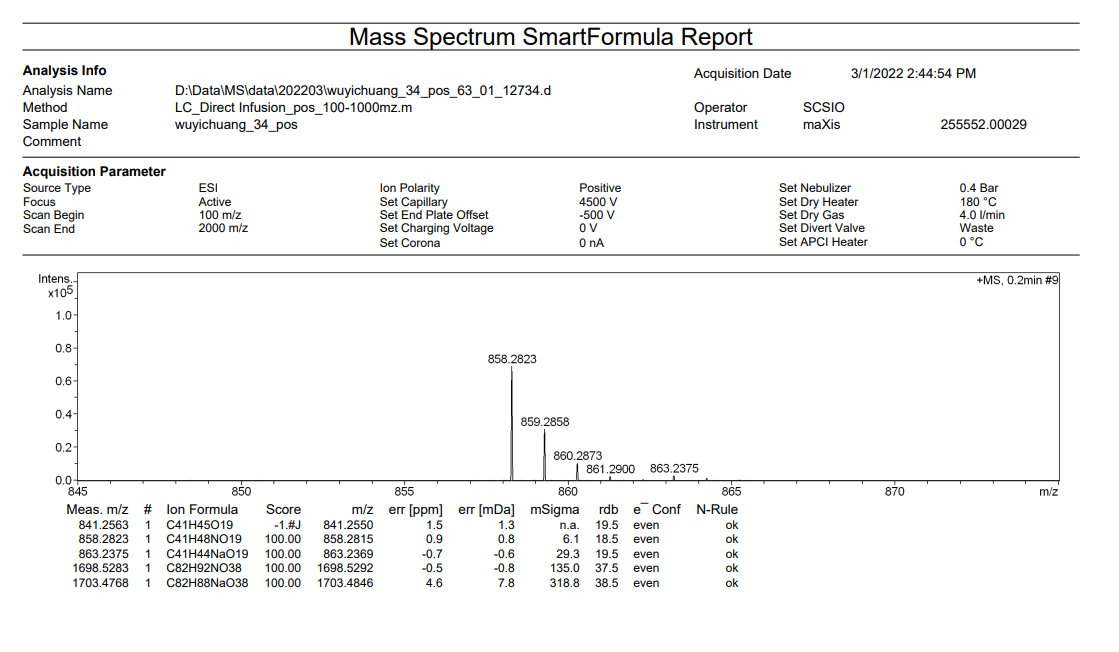


**Figure S4**. HR-FAB-MS spectrum of compound **33**


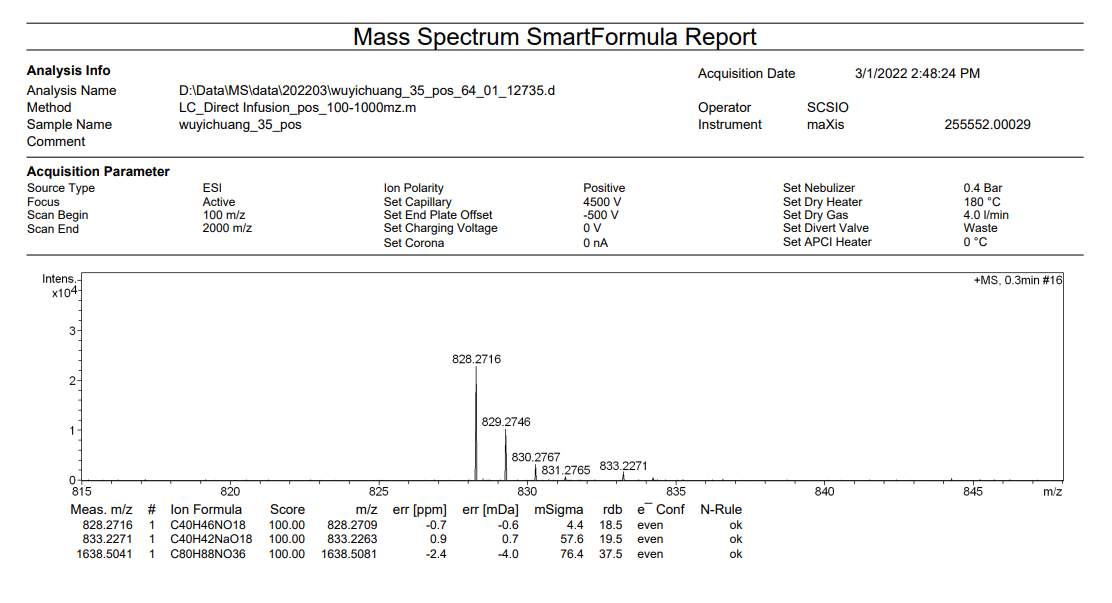
**Figure S5**. HR-FAB-MS spectrum of compound **34**

**Figure S6.** UV spectrum of compound **30**

**Figure S7.** UV spectrum of compound **31**

**Figure S8.** UV spectrum of compound **32**

**Figure S9.** UV spectrum of compound **33**

**Figure S10.** UV spectrum of compound **34**


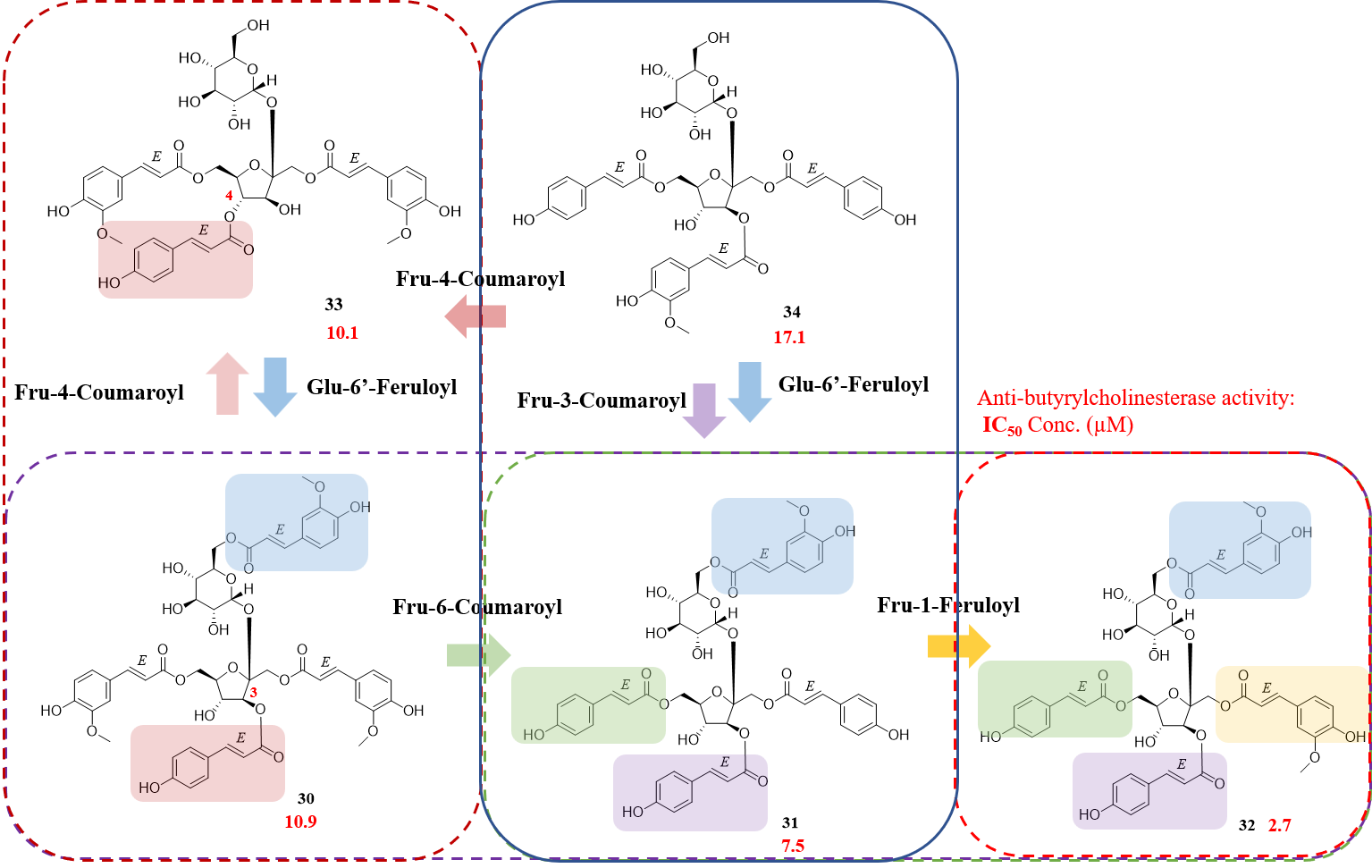


**Figure S11.** Graphical depiction of the structure-activity relationships (SARs) of compounds **30**–**34**

**Table S1**. ^1^H (600 MHz, CD_3_OD-*d_4_*) and ^13^C NMR (151 MHz, CD_3_OD-*d_4_*) Data for **30**–**32**.

|  | **30** | |  | **31** | |  | **32** | |
| --- | --- | --- | --- | --- | --- | --- | --- | --- |
|  | ***δ*_H_** | ***δ_C_*** |  | ***δ*_H_** | ***δ_C_*** |  | ***δ*_H_** | ***δ_C_*** |
| fructose |  |  |  |  |  |  |  |  |
| 1 | 4.32 (2H, m) | 66.3 |  | 4.54 (2H, m) | 66.3 |  | 4.31 (2H, m) | 66.3 |
| 2 |  | 103.4 |  |  | 103.4 |  |  | 103.4 |
| 3 | 5.64 (d, 8.4) | 79.1 |  | 5.64 (d, 8.7) | 79.1 |  | 5.63 (d, 8.7) | 79.1 |
| 4 | 4.73 (m) | 73.9 |  | 4.73(t, 8.8) | 81.0 |  | 4.71 (m) | 73.9 |
| 5 | 4.20 (m) | 81.1 |  | 4.20 (m) | 65.5 |  | 4.20 (m) | 81.0 |
| 6 | 4.53-4.59 (m) | 65.4 |  | 4.56 (2H, m) |  |  | 4.56- 4.58 (m) | 65.4 |
| glucose |  |  |  |  |  |  |  |  |
| 1' | 5.56 (d, 3.8) | 93.0 |  | 5.56 (d, 3.8) | 92.9 |  | 5.56 (d, 3.8) | 92.9 |
| 2' | 3.47 (m) | 72.9 |  | 3.46 (dd, 9.8, 3.8) | 72.9 |  | 3.46 (dd, 9.8, 3.8) | 72.9 |
| 3' | 3.65 (m) | 74.9 |  | 3.65 (t, 9.3) | 75.0 |  | 3.65 (dd, 9.3, 9.0 | 74.9 |
| 4' | 3.30 (m) | 72.3 |  | 3.31 (m) | 72.3 |  | 3.30 (m) | 72.2 |
| 5' | 3.31 (m) | 72.4 |  | 4.54 (m) | 72.4 |  | 3.30 (m) | 72.4 |
| 6' | 4.73 (m), 4.20 (m) | 65.7 |  | 4.18-4.24 (m) | 65.8 |  | 4.71 (m), 4.20 (m) | 65.7 |
| phenylpropanoids  (glc-6’) | feruloyl |  |  | feruloyl |  |  | feruloyl |  |
| 9'' |  | 169.3 |  |  | 169.3 |  |  | 169.3 |
| 8'' | 6.46 (d, 15.9) | 115.2 |  | 6.48 (d, 15.9) | 115.3 |  | 6.45 (d, 15.9) | 115.3 |
| 7'' | 7.65 (d, 15.9) | 147.2 |  | 7.62 (d, 15.9) | 147.2 |  | 7.64 (d, 15.9) | 147.2 |
| 1'' |  | 127.6 |  |  | 127.7 |  |  | 127.6 |
| 2'' | 7.20 (d, 1.7) | 111.5 |  | 7.21 (d, 1.5) | 111.5 |  | 7.14 (d, 2.0) | 111.5 |
| 3'' |  | 149.3 |  |  | 149.4 |  |  | 148.3 |
| 4'' |  | 150.8 |  |  | 150.7 |  |  | 150.7 |
| 5'' | 6.73-6.79 | 116.8 |  | 6.79 (d, 8.6) | 116.3 |  | 6.76 (d, 8.3) | 116.5 |
| 6'' | 7.03 (d, 8.3, 1.7) | 124.4 |  | 7.01 (dd, 8.3, 1.5) | 124.6 |  | 6.96 (dd, 8.3, 2.0) | 124.5 |
| O-Me | 3.88 (3H, s) | 56.5 |  |  | 56.5 |  | 3.84 (3H, s) | 56.5 |
| (fruc-1) | feruloyl |  |  | *p*-coumaroy |  |  | feruloyl |  |
| 9''' |  | 168.5 |  |  | 168.5 |  |  | 168.5 |
| 8''' | 6.40 (d, 15.9) | 114.9 |  | 6.35 (d, 15.9) | 114.7 |  | 6.33 (d, 15.9) | 115.1 |
| 7''' | 7.66 (d, 15.9) | 147.5 |  | 7.66 (d, 15.9) | 147.9 |  | 7.70 (d, 15.9) | 147.2 |
| 1''' |  | 127.6 |  |  | 127.0 |  |  | 127.6 |
| 2''' | 7.14 (d, 1.7) | 111.6 |  | 7.43 (d, 8.6) | 131.3 |  | 7.12 (d, 1.8) | 111.6 |
| 3''' |  | 149.4 |  | 6.79 (d, 8.6) | 116.8 |  |  | 149.3 |
| 4''' |  | 151.1 |  |  | 161.7 |  |  | 150.8 |
| 5''' | 6.73-6.79 | 116.3 |  | 6.79 (d, 8.6) | 116.8 |  | 6.76 (d, 8.3) | 116.3 |
| 6''' | 7.08 (d, 8.3, 1.7) | 124.2 |  | 7.43 (d, 8.6) | 131.3 |  | 6.98 (dd, 8.3, 2.0) | 124.2 |
| O-Me | 3.83 (3H, s) | 56.4 |  |  |  |  | 3.83 (3H, s) | 56.4 |
| (fruc-3) | *p*-coumaroyl |  |  | fruc-(*p*-coumaroy） |  |  | *p*-coumaroyl |  |
| 9'''' |  | 168.4 |  |  | 168.5 |  |  | 168.5 |
| 8'''' | 6.44 (d, 15.9) | 114.2 |  | 6.44 (d, 15.9) | 114.3 |  | 6.43 (d, 15.9) | 114.3 |
| 7'''' | 7.70 (d, 15.9) | 147.9 |  | 7.72 (d, 15.9) | 147.2 |  | 7.70 (d, 15.9) | 147.9 |
| 1'''' |  | 127.0 |  |  | 127.0 |  |  | 127.0 |
| 2'''', 6'''' | 7.46 (d, 8.7) | 131.5 |  | 7.49 (d, 8.6) | 131.3 |  | 7.47 (d, 8.6) | 131.6 |
| 3'''', 5'''' | 6.73-6.79 | 116.9 |  | 6.79 (d, 8.6) | 116.8 |  | 6.76 (d, 8.3) | 116.8 |
| 4'''' |  | 161.7 |  |  | 161.7 |  |  | 161.6 |
| (fruc-6) | feruloyl |  |  | *p*-coumaroy |  |  | *p*-coumaroyl |  |
| 9''''' |  | 168.9 |  |  | 168.9 |  |  | 168.9 |
| 8''''' | 6.33 (d, 15.9) | 115.1 |  | 6.27 (d, 15.9) | 114.8 |  | 6.29 (d, 15.9) | 114.6 |
| 7''''' | 7.68 (d, 15.9) | 147.1 |  | 7.58 (d, 16.0) | 146.9 |  | 7.59 (d, 15.9) | 147.1 |
| 1''''' |  | 127.5 |  |  | 127.0 |  |  | 127.0 |
| 2''''', 6'''''(2''''') | 7.14 (d, 1.7) | 111.6 |  | 7.35 (d, 8.5) | 131.2 |  | 7.40 (d, 8.5) | 131.3 |
| 3''''', 5'''''(3''''') |  | 149.4 |  | 6.77 (d, 8.6) | 116.8 |  | 6.78 (d, 8.6) | 116.9 |
| 4''''' |  | 150.9 |  |  | 161.4 |  |  | 161.4 |
| (5''''') | 6.73-6.79 | 116.5 |  |  |  |  |  |  |
| (6''''') | 7.03 (d, 8.3, 1.7) | 124.2 |  |  |  |  |  |  |
| (O-Me) | 3.85 (3H, s) | 56.4 |  |  |  |  |  |  |

**Table S2**. ^1^H (400 MHz, CD_3_OD-*d_4_*) and ^13^C NMR (101 MHz, CD_3_OD-*d_4_*) Data for **33**, ^1^H (600 MHz, CD_3_OD-*d_4_*) and ^13^C NMR (151 MHz, CD_3_OD-*d_4_*) Data for **34**.

|  | **33** | |  | **34** | |
| --- | --- | --- | --- | --- | --- |
|  | ***δ*_H_** | ***δ_C_*** |  | ***δ*_H_** | ***δ_C_*** |
| fructose |  |  |  |  |  |
| 1 | 4.34 (2H, m) | 66.5 |  | 3.60 (2H, m) | 63.9 |
| 2 |  | 103.6 |  |  | 104.4 |
| 3 | 5.67 (d, 8.6) | 79.3 |  | 5.44 (d, 7.5) | 78.7 |
| 4 | 4.51 (m) | 73.1 |  | 4.60 (m) | 76.3 |
| 5 | 4.33 (m) | 81.2 |  | 4.12 (m) | 80.9 |
| 6 | 4.59 (2H, m) | 65.9 |  | 4.49 (2H, m) | 66.5 |
| glucose |  |  |  |  |  |
| 1' | 5.59 (d, 3.7) | 93.1 |  | 5.51 (d, 3.4) | 93.9 |
| 2' | 3.46 (d, 9.7) | 72.6 |  | 3.47 (dd, 9.7, 3.8) | 73.1 |
| 3' | 3.68 (t, 9.2) | 75.1 |  | 3.60 (dd, 9.6, 9.0) | 74.7 |
| 4' | 3.49 (t, 9.5) | 72.4 |  | 3.19 (m) | 72.2 |
| 5' | 3.86 (m) | 74.1 |  | 3.23 (m) | 72.3 |
| 6' | 3.81-3.90(m) | 65.7 |  | 4.63 (m), 4.20 (m) | 65.5 |
| phenylpropanoids  (glc-6’) |  |  |  | feruloyl |  |
| 9'' |  |  |  |  | 169.3 |
| 8'' |  |  |  | 6.43 (d, 15.9) | 115.3 |
| 7'' |  |  |  | 7.54 (d, 15.9) | 147.2 |
| 1'' |  |  |  |  | 127.7 |
| 2'' |  |  |  | 7.19 (d, 1.9) | 111.6 |
| 3'' |  |  |  |  | 150.6 |
| 4'' |  |  |  |  | 150.6 |
| 5'' |  |  |  | 6.75 (d, 8.1) | 116.3 |
| 6'' |  |  |  | 6.95 (dd, 8.2, 1.9) | 124.2 |
| O-Me |  |  |  | 3.84 (3H, s) | 56.4 |
| (fruc-1) | feruloyl |  |  |  |  |
| 9''' | 7.62 (d, 16.2) | 169.1 |  |  |  |
| 8''' | 6.35 (d, 15.6) | 114.5 |  |  |  |
| 7''' |  | 148.1 |  |  |  |
| 1''' |  | 127.9 |  |  |  |
| 2''' | 7.19 (d, 1.5) | 111.9 |  |  |  |
| 3''' |  | 149.5 |  |  |  |
| 4''' |  | 150.8 |  |  |  |
| 5''' | 6.81 (d,8.8) | 116.5 |  |  |  |
| 6''' | 7.09 (d, 8.9) | 124.7 |  |  |  |
| O-Me | 3.86 (s) | 56.7 |  |  |  |
| (fruc-4or3) | fruc-4(*p*-coumaroy) |  |  | *p*-coumaroy |  |
| 9'''' | 7.62 (d, 16.2) | 168.7 |  |  | 168.5 |
| 8'''' | 6.35 (d, 15.6) | 114.9 |  | 6.43 (d, 15.9) | 114.6 |
| 7'''' |  | 147.4 |  | 7.66 (d, 15.9) | 149.3 |
| 1'''' |  | 127.3 |  |  | 127.6 |
| 2'''', 6'''' | 7.42 (d, 8.4) | 131.5 |  | 7.46 (d, 8.7) | 131.3 |
| 3'''', 5'''' | 6.76 (d, 8.7) | 117.0 |  | 6.79 (d, 8.3) | 116.9 |
| 4'''' |  | 161.7 |  |  | 161.5 |
| (fruc-6) | feruloyl |  |  | *p*-coumaroy |  |
| 9''''' | 7.62 (d, 16.2) | 169.5 |  |  | 168.9 |
| 8''''' | 6.46 (d, 15.9) | 115.4 |  | 6.29 (d, 15.9) | 115.2 |
| 7''''' |  | 147.3 |  | 7.54 (d, 15.9) | 147.1 |
| 1''''' |  | 127.9 |  |  | 127.0 |
| 2''''', 6'''''(2''''') | 7.14 (d, 1.5) | 111.7 |  | 7.41 (d, 8.7) | 131.3 |
| 3''''', 5'''''(3''''') |  | 149.5 |  | 6.71 (d, 8.2) | 116.4 |
| 4''''' |  | 150.7 |  |  | 160.2 |
| (5''''') | 6.76 (d, 8.7) | 115.5 |  |  |  |
| (6''''') | 7.09 (d, 8.9) | 124.4 |  |  |  |
| (O-Me) | 3.84 (s, 3H) | 56.4 |  |  |  |

**Table S3**. ^1^H (600 MHz, DMSO-*d*_6_) and ^13^C NMR (151 MHz, DMSO-*d*_6_) Data for **1** and **3**, ^1^H (400 MHz, DMSO-*d*_6_) and ^13^C NMR (101 MHz, DMSO-*d*_6_) Data for**2**.

| Position | **1** | |  | **2** | |  | **3** | |
| --- | --- | --- | --- | --- | --- | --- | --- | --- |
|  | ***δ*_H_** | ***δ_C_*** |  | ***δ*_H_** | ***δ_C_*** |  | ***δ*_H_** | ***δ_C_*** |
| 2 |  | 121.2 |  |  | 163.5 |  |  | 146.8 |
| 3 | 6.74 (s) | 102.9 |  | 6.73 (s) | 102.8 |  |  | 135.7 |
| 4 |  | 181.8 |  |  | 181.9 |  |  | 175.9 |
| 5 |  | 157.3 |  |  | 161.2 |  |  | 160.7 |
| 6 | 6.44 (d, 2.1) | 98.9 |  |  | 108.9 |  | 6.27 (d, 1.6) | 98.2 |
| 7 |  | 164.2 |  |  | 163.5 |  |  | 163.9 |
| 8 | 6.15 (d, 2.1) | 93.9 |  | 6.47 (s) | 93.7 |  | 6.41(d, 1.6) | 93.5 |
| 9 |  | 161.5 |  |  | 156.3 |  |  | 156.2 |
| 10 |  | 103.7 |  |  | 103.4 |  |  | 103.1 |
| 1' |  | 164.2 |  |  | 121.1 |  |  | 121.7 |
| 2' | 6.88 (d, 8.8) | 115.9 |  | 6.88 (d, 8.4) | 128.5 |  | 8.01 (d, 8.8) | 129.5 |
| 3' | 7.88 (d, 8.8) | 128.5 |  | 7.88 (d, 8.4) | 116.0 |  | 6.93 (d, 8.8) | 115.5 |
| 4' |  | 161.2 |  |  | 160.7 |  |  | 159.2 |
| 5' | 7.88 (d, 8.8) | 128.5 |  | 7.88 (d, 8.4) | 116.0 |  | 6.93 (d, 8.8) | 115.5 |
| 6' | 6.88 (d, 8.8) | 115.9 |  | 6.88 (d, 8.4) | 128.5 |  | 8.01 (d, 8.8) | 129.5 |
| 1'' |  |  |  | 4.55 (d, 9.8) | 73.1 |  |  |  |
| 2'' |  |  |  | 4.00 (s) | 70.6 |  |  |  |
| 3'' |  |  |  |  | 78.9 |  |  |  |
| 4'' |  |  |  |  | 70.3 |  |  |  |
| 5'' |  |  |  |  | 81.5 |  |  |  |
| 6'' |  |  |  |  | 61.5 |  |  |  |

**Table S4**. ^1^H (600 MHz, CD_3_OD-*d_4_*) and ^13^C NMR (151 MHz, CD_3_OD-*d_4_*) Data for **4**–**6**.

|  | **4** | |  | **5** | |  | **6** | |
| --- | --- | --- | --- | --- | --- | --- | --- | --- |
|  | ***δ*_H_** | ***δ_C_*** |  | ***δ*_H_** | ***δ_C_*** |  | ***δ*_H_** | ***δ_C_*** |
| 2 |  | 159.2 |  |  | 156.3 |  |  | 159.4 |
| 3 |  | 136.2 |  |  | 133.2 |  |  | 135.5 |
| 4 |  | 179.5 |  |  | 177.5 |  |  | 179.4 |
| 5 |  | 163.2 |  |  | 159.9 |  |  | 163.0 |
| 6 |  | 100.0 |  | 6.16 (d, 2.1) | 98.7 |  | 6.21 (d, 1.5) | 100.0 |
| 7 |  | 166.34 |  |  | 164.2 |  |  | 166.1 |
| 8 |  | 94.9 |  | 6.39 (d, 2.1) | 93.7 |  | 6.40(s) | 94.9 |
| 9 |  | 158.6 |  |  | 156.4 |  |  | 158.6 |
| 10 |  | 105.8 |  |  | 104.0 |  |  | 105.6 |
| 1' |  | 122.6 |  |  | 120.9 |  |  | 122.8 |
| 2' | 7.73 (d, 8.6) | 131.9 |  | 8.01 (d, 7.2) | 131.3 |  | 8.06(d, 8.7) | 132.4 |
| 3' | 6.90 (d, 8.6) | 116.5 |  | 6.86 (d, 7.2) | 115.5 |  | 6.89(d, 8.7) | 116.1 |
| 4' |  | 161.6 |  |  | 161.6 |  |  | 161.5 |
| 5' | 6.90 (d, 8.6) | 116.5 |  | 6.86 (d, 7.2) | 115.5 |  | 6.89(d, 8.7) | 116.1 |
| 6' | 7.73 (d, 8.6) | 131.9 |  | 8.01 (d, 7.2) | 131.3 |  | 8.06(d, 8.7) | 132.4 |
| 1'' | 5.34 (d, 1.2) | 103.5 |  | 5.41 (d, 7.4) | 101.2 |  | 5.12(d, 7.3) | 104.6 |
| 2'' | 4.20 (d, 1.3) | 71.9 |  |  | 74.2 |  |  | 71.4 |
| 3'' | 3.69 (dd ,8.9, 3.2) | 72.1 |  |  | 76.4 |  | 3.47(m) | 78.1 |
| 4'' | 3.31 (dd, 7.3, 5.2) | 72.0 |  |  | 69.9 |  | 3.47(m) | 75.8 |
| 5'' | 3.31 (dd, 7.3, 5.2) | 73.2 |  |  | 77.5 |  | 3.47(m) | 69.7 |
| 6'' | 0.89 (t, 6.4) | 17.6 |  |  | 60.9 |  |  | 68.6 |
| 1''' |  |  |  |  |  |  | 4.51(s) | 102.4 |
| 2''' |  |  |  |  |  |  | 3.63(s) | 72.1 |
| 3''' |  |  |  |  |  |  | 3.52(d, 3.3) | 72.3 |
| 4''' |  |  |  |  |  |  |  | 77.2 |
| 5''' |  |  |  |  |  |  |  | 73.9 |
| 6''' |  |  |  |  |  |  | 1.09(d, 6.2) | 17.9 |

**Table S5**. ^1^H (600 MHz, DMSO-*d*_6_) and ^13^C NMR (151 MHz, DMSO-*d*_6_) Data for **7** and **8**, ^1^H (600 MHz, CD_3_OD-*d_4_*) and ^13^C NMR (151 MHz, CD_3_OD-*d_4_*) Data for **9**.

| Position | **7** | |  | **8** | |  | **9** | |
| --- | --- | --- | --- | --- | --- | --- | --- | --- |
|  | ***δ*_H_** | ***δ_C_*** |  | ***δ*_H_** | ***δ_C_*** |  | ***δ*_H_** | ***δ_C_*** |
| 2 |  | 164.2 |  |  | 147.8 |  |  | 158.4 |
| 3 | 6.66 (s) | 102.9 |  |  | 135.8 |  |  | 135.6 |
| 4 |  | 181.7 |  |  | 175.9 |  |  | 179.4 |
| 5 |  | 157.3 |  |  | 160.8 |  |  | 163.0 |
| 6 | 6.18 (d, 1.8) | 98.9 |  | 6.12 (d, 1.3) | 98.2 |  | 6.16 (s) | 99.9 |
| 7 |  | 163.9 |  |  | 163.9 |  |  | 166.0 |
| 8 | 6 .44 (d, 1.8) | 93.9 |  | 6.34 (s) | 93.4 |  | 6.35 (s) | 94.7 |
| 9 |  | 161.5 |  |  | 156.2 |  |  | 158.6 |
| 10 |  | 103.7 |  |  | 103.1 |  |  | 105.6 |
| 1' |  | 121.5 |  |  | 122.0 |  |  | 122.8 |
| 2' | 7.39 (d, 1.9) | 113.4 |  | 7.60 (d, 1.8) | 115.1 |  | 7.72 (s) | 117.5 |
| 3' |  | 145.8 |  |  | 146.9 |  |  | 145.9 |
| 4' |  | 149.7 |  |  | 145.1 |  |  | 149.9 |
| 5' | 6.89 (d, 8.2) | 116.1 |  | 6.82 (d, 8.4) | 115.7 |  | 6.84 (d, 8.4) | 123.0 |
| 6' | 7.42 (dd, 8.4, 2.3) | 119.0 |  | 7.47 (d, 8.4) | 120.0 |  | 7.54 (dd, 8.4, 1.5) | 116.2 |
| 1'' |  |  |  |  |  |  | 5.13 (d, 6.6) | 104.7 |
| 2'' |  |  |  |  |  |  |  | 72.9 |
| 3'' |  |  |  |  |  |  |  | 74.1 |
| 4'' |  |  |  |  |  |  |  | 69.1 |
| 5'' |  |  |  |  |  |  |  | 67.0 |
| 6'' |  |  |  |  |  |  |  |  |

**Table S6**. ^1^H (600 MHz, DMSO-*d*_6_) and ^13^C NMR (151 MHz, DMSO-*d*_6_) Data for **10**, ^1^H (600 MHz, CD_3_OD-*d_4_*) and ^13^C NMR (151 MHz, CD_3_OD-*d_4_*) Data for **11** and **12**.

| Position | **10** | |  | **11** | |  | **12** | |
| --- | --- | --- | --- | --- | --- | --- | --- | --- |
|  | ***δ*_H_** | ***δ_C_*** |  | ***δ*_H_** | ***δ_C_*** |  | ***δ*_H_** | ***δ_C_*** |
| 2 |  | 156.4 |  |  | 158.8 |  |  | 158.5 |
| 3 |  | 133.4 |  |  | 136.4 |  |  | 136.2 |
| 4 |  | 177.4 |  |  | 179.6 |  |  | 179.6 |
| 5 |  | 161.2 |  |  | 158.7 |  |  | 163.1 |
| 6 | 6.17 (s) | 98.9 |  | 6.35 (s) | 95.0 |  | 6.13 (d, 6.3) | 99.8 |
| 7 |  | 164.9 |  |  | 167.1 |  |  | 165.8 |
| 8 | 6.38 (s) | 93.6 |  | 6.19 (d, 1.5) | 100.2 |  | 6.13 (d, 3.7) | 94.7 |
| 9 |  | 156.1 |  |  | 163.2 |  |  | 159.3 |
| 10 |  | 103.7 |  |  | 105.7 |  |  | 105.9 |
| 1' |  | 121.1 |  |  | 124.4 |  |  | 122.9 |
| 2' | 7.52 (s) | 115.2 |  |  | 116.6 |  | 7.25 (dd, 8.3, 1.6) | 116.3 |
| 3' |  | 144.9 |  | 7.34 (d, 1.9) | 147.7 |  |  | 149.7 |
| 4' |  | 148.6 |  |  | 151.6 |  |  | 146.4 |
| 5' | 6.81 (d, 8.4) | 115.9 |  | 7.08 (d, 8.5) | 112.4 |  | 6.85 (d, 8.3) | 116.9 |
| 6' | 7.66 (d, 8.4) | 122.0 |  | 7.41 (d, 8.4) | 122.7 |  | 7.28 (d, 1.4) | 122.9 |
| 1'' | 5.37 (d, 7.7) | 101.9 |  | 5.37 (d, 1.0) | 103.5 |  | 5.30 (s) | 103.5 |
| 2'' | 3.55 (m) | 71.2 |  | 4.21 (d, 1.3) | 71.9 |  | 3.65–3.75 (m**)** | 72.1 |
| 3'' |  | 73.2 |  | 3.74 – 3.71 (m) | 72.0 |  | 3.30–3.45 (m**'**) | 72.0 |
| 4'' | 3.65 (s) | 67.9 |  | 3.35 – 3.31 (m) | 72.1 |  | 3.20–3.35 (m) | 73.2 |
| 5'' |  | 75.9 |  | 3.35 – 3.31 (m) | 73.2 |  | 4.15–4.25 (m) | 71.9 |
| 6'' |  | 60.2 |  | 0.91 (s) | 17.7 |  | 0.89 (d, 6.1) | 17.6 |
| OCH_3_ |  |  |  | 3.94 (s) | 56.4 |  |  |  |

**Table S7**. ^1^H (600 MHz, DMSO-*d*_6_) and ^13^C NMR (151 MHz, DMSO-*d*_6_) Data for **13**, ^1^H (600 MHz, CD_3_OD-*d_4_*) and ^13^C NMR (151 MHz, CD_3_OD-*d_4_*) Data for **14**, ^1^H (400 MHz, DMSO-*d*_6_) and ^13^C NMR (101 MHz, DMSO-*d*_6_) Data for **15**.

| Position | **13** | |  | **14** | |  | **15** | |
| --- | --- | --- | --- | --- | --- | --- | --- | --- |
|  | ***δ*_H_** | ***δ_C_*** |  | ***δ*_H_** | ***δ_C_*** |  | ***δ*_H_** | ***δ_C_*** |
| 2 |  | 156.2 |  |  | 158.5 |  |  | 156.4 |
| 3 |  | 133.4 |  |  | 135.6 |  |  | 133∙5 |
| 4 |  | 177.5 |  |  | 179.3 |  |  | 177.4 |
| 5 |  | 104.0 |  |  | 162.9 |  |  | 161.2 |
| 6 | 6.20 (d, 2.0) | 161.3 |  | 6.16 (d, 1.7) | 100.0 |  | 6.19 (s) | 98.8 |
| 7 |  | 98.8 |  |  | 166.1 |  |  | 164.5 |
| 8 | 6.45 (d, 1.9) | 164.3 |  | 6.35 (d, 1.7) | 94.9 |  | 6.39 (s) | 93.6 |
| 9 |  | 156.4 |  |  | 159.3 |  |  | 156.5 |
| 10 |  | 93.6 |  |  | 105.6 |  |  | 103.8 |
| 1' |  | 121.2 |  |  | 123.1 |  |  | 121.1 |
| 2' | 7.65 (d, 1.9) | 115.3 |  | 7.64 (d, 1.8) | 117.7 |  | 7∙53 (s) | 115.2 |
| 3' |  | 144.9 |  |  | 145.8 |  |  | 144.8 |
| 4' |  | 148.5 |  |  | 149.8 |  |  | 148.6 |
| 5' | 6.95 (d, 8.1) | 116.3 |  | 6.84 (d, 8.4) | 116.0 |  | 6.82 (d, 8.4) | 116.0 |
| 6' | 7.71 (dd, 8.1, 1.9) | 121.7 |  | 7.59 (dd, 8.4, 1.8) | 123.6 |  | 7.64 (d, 8.5) | 121.1 |
| 1'' | 5.08 (s) | 100.9 |  | 5.07 (d, 7.7) | 104.8 |  | 5.31 (d, 7.7) | 102.1 |
| 2'' | 3.05−3.90 (m) | 74.2 |  |  | 75.7 |  |  | 71.1 |
| 3'' | 3.05−3.90 (m) | 76.6 |  |  | 78.1 |  |  | 73∙1 |
| 4'' | 3.05−3.90 (m) | 70.0 |  |  | 71.4 |  |  | 68.1 |
| 5'' | 3.05−3.90 (m) | 77.6 |  |  | 77.2 |  |  | 73∙6 |
| 6'' | 3.05−3.90 (m) | 61.0 |  |  | 68.5 |  |  | 65.2 |
| 1''' |  |  |  | 4.49 (s) | 102.4 |  |  | 100.0 |
| 2''' |  |  |  |  | 72.1 |  |  | 70.5 |
| 3''' |  |  |  |  | 72.2 |  |  | 70.6 |
| 4''' |  |  |  |  | 73.9 |  |  | 72.0 |
| 5''' |  |  |  |  | 69.7 |  |  | 68∙3 |
| 6''' |  |  |  | 1.09 (d, 6.2) | 17.9 |  | 1.0 (m) | 17∙9 |

**Table S8**. ^1^H (600 MHz, DMSO-*d*_6_) and ^13^C NMR (151 MHz, DMSO-*d*_6_) Data for **16** and **17**, ^1^H (400 MHz, CD_3_OD-*d_4_*) and ^13^C NMR (101 MHz, CD_3_OD-*d_4_*) Data for **18**, ^1^H (600 MHz, CD_3_OD-*d_4_*) and ^13^C NMR (151 MHz, CD_3_OD-*d_4_*) Data for **19**.

| Position | **16** | | **17** | | **18** | | **19** | |
| --- | --- | --- | --- | --- | --- | --- | --- | --- |
|  | ***δ*_H_** | ***δ_C_*** | ***δ*_H_** | ***δ_C_*** | ***δ*_H_** | ***δ_C_*** | ***δ*_H_** | ***δ_C_*** |
| 2 |  | 146.9 |  | 157.5 | 4.63 (d, 7.5) | 82.9 | 4.59 (s) | 79.9 |
| 3 |  | 135.9 |  | 134.3 | 4.04 (dd, 13.4, 7.5) | 68.8 | 4.24 – 4.13 (m) | 67.5 |
| 4 |  | 175.8 |  | 177.8 | 2.91 (dd, 16.1, 5.4)  2.57 (dd, 16.1, 8.1) | 28.5 | 2.87 (dd, 16.7, 4.6)  2.74 (dd, 16.8, 2.8) | 29.3 |
| 5 | 12.45 (s) | 160.7 |  | 161.3 |  | 157.6 |  | 157.7 |
| 6 | 6.14 (s) | 98.2 | 6.17 (s) | 98.7 | 5.92 (s) | 96.3 | 5.92 (d, 2.3) | 96.4 |
| 7 | 10.75 (s) | 163.9 |  | 164.2 |  | 157.8 |  | 158.0 |
| 8 | 6.33 (s) | 93.2 | 6.34 (s) | 93.5 | 5.98 (d, 14.4) | 95.5 | 5.94 (d, 2.3) | 95.9 |
| 9 |  | 156.1 |  | 156.4 |  | 156.9 |  | 157.4 |
| 10 |  | 103.0 |  | 119.6 |  | 100.9 |  | 100.1 |
| 1' |  | 120.8 |  | 104.1 |  | 132.2 |  | 132.3 |
| 2' | 7.20 (d, 4.1) | 107.2 | 6.85 (s) | 107.9 | 6.90 (s) | 115.3 | 6.98 (d, 1.9) | 115.3 |
| 3' |  | 145.7 |  | 145.8 |  | 146.2 |  | 145.9 |
| 4' | 8.77 (s) | 135.9 |  | 136.5 |  | 146.2 |  | 145.8 |
| 5' |  | 145.7 |  | 145.8 | 6.83 (d, 8.1) | 116.1 | 6.76 (d, 8.1) | 115.9 |
| 6' | 7.20 (d, 4.1) | 107.2 | 6.85 (s) | 107.9 | 6.78 (d, 8.1) | 120.0 | 6.80 (dd, 8.2, 1.8) | 119.4 |
| 1'' |  |  | 5.16 (s) | 102.0 |  |  |  |  |
| 2'' |  |  |  | 70.0 |  |  |  |  |
| 3'' |  |  |  | 70.4 |  |  |  |  |
| 4'' |  |  |  | 71.3 |  |  |  |  |
| 5'' |  |  |  | 70.6 |  |  |  |  |
| 6'' |  |  | 0.81(d, 0.58) | 17.6 |  |  |  |  |

**Table S9**. ^1^H (600 MHz, CD_3_OD-*d_4_*) and ^13^C NMR (151 MHz, CD_3_OD-*d_4_*) Data for **20**.

| **20** | | | | | |
| --- | --- | --- | --- | --- | --- |
| Position | ***δ*_H_** | ***δ_C_*** | Position | ***δ*_H_** | ***δ_C_*** |
| 2 |  | 159.0 | 8'' | 6.29(s) | 94.7 |
| 3 |  | 135.6 | 9'' |  | 158.4 |
| 4 |  | 179.4 | 10'' |  | 105.5 |
| 5 |  | 162.9 | 1''' |  | 122.8 |
| 6 | 6.11(s) | 99.9 | 2''' | 7.79(d, 2.0) | 117.8 |
| 7 |  | 166.0 | 3''' |  | 145.7 |
| 8 | 6.29(s) | 94.7 | 4''' |  | 149.9 |
| 9 |  | 158.3 | 5''' | 7.59(d, 8.5) | 116.0 |
| 10 |  | 105.6 | 6''' | 6.79(d, 8.5) | 123.2 |
| 1' |  | 122.9 | 1'''' | 5.09(d, 7.7) | 104.4 |
| 2' | 7.65(d, 2.0) | 117.6 | 2'''' |  | 75.7 |
| 3' |  | 145.8 | 3'''' |  | 78.1 |
| 4' |  | 149.8 | 4'''' |  | 71.2 |
| 5' | 7.59(d, 8.5) | 116.0 | 5'''' |  | 78.3 |
| 6' | 6.81(d, 8.5) | 123.0 | 6'''' |  | 62.5 |
| 2'' |  | 158.7 | 1''''' | 5.18(s) | 105.5 |
| 3'' |  | 135.8 | 2''''' |  | 73.2 |
| 4'' |  | 179.4 | 3''''' | 3.79(t,8.9) | 75.1 |
| 5'' |  | 162.9 | 4''''' | 3.83(d, 3.4) | 70.0 |
| 6'' | 6.11(s) | 99.9 | 5''''' |  | 77.1 |
| 7'' |  | 166.0 | 6''''' | 3.69(dd,11.8,2.4)  3.61(dd,10.9,5.8) | 61.9 |

**Table S10**. ^1^H (400 MHz, DMSO-*d*_6_) and ^13^C NMR (101 MHz, DMSO-*d*_6_) Data for **21** and **22**, ^1^H (600 MHz, CD_3_OD-*d_4_*) and ^13^C NMR (151 MHz, CD_3_OD-*d_4_*) Data for **23**.

| Position | **21** | |  | **22** | |  | **23** | |
| --- | --- | --- | --- | --- | --- | --- | --- | --- |
|  | ***δ*_H_** | ***δ_C_*** |  | ***δ*_H_** | ***δ_C_*** |  | ***δ*_H_** | ***δ_C_*** |
| 1 | 12.05 (s) | 164.2 |  | 12.05 (s) | 164.4 |  |  | 162.0 |
| 2 | 6.56 (d, 2.4) | 107.7 |  | 6.56 (d, 2.4) | 107.9 |  | 7.30 (s) | 110.7 |
| 3 |  | 165.4 |  |  | 165.5 |  |  | 163.5 |
| 3-OCH_3_ |  |  |  |  | 48.6 |  |  |  |
| 4 | 7.12 (d, 2.4) | 108.7 |  | 7.12 (d, 2.4) | 108.7 |  | 7.44 (d, 2.2) | 112.2 |
| 4a |  | 134.8 |  |  | 135.0 |  |  | 137.8 |
| 5 | 7.44 (s) | 120.2 |  | 7.44 (s) | 120.4 |  | 7.34 (d, 2.2) | 121.1 |
| 6 |  | 148.0 |  |  | 148.2 |  |  | 149.0 |
| 6- CH_3_ | 2.39 (s) | 21.3 |  | 2.39 (s) | 21.5 |  | 2.34 (s) | 22.0 |
| 7 | 7.08 (d, 2.4) | 123.9 |  | 7.08 (d, 2.4) | 124.1 |  | 6.93 (s) | 125.5 |
| 8 | 11.97 (s) | 161.2 |  | 11.97 (s) | 161.4 |  |  | 163.1 |
| 8a |  | 113.0 |  |  | 113.3 |  |  | 117.5 |
| 9 |  | 189.4 |  |  | 189.6 |  |  | 188.5 |
| 9a |  | 108.6 |  |  | 108.9 |  |  | 115.8 |
| 10 |  | 181.0 |  |  | 181.2 |  |  | 182.7 |
| 10a |  | 132.5 |  |  | 132.7 |  |  | 133.4 |
| 1' |  |  |  |  |  |  |  | 104.2 |
| 2' |  |  |  |  |  |  |  | 74.7 |
| 3' |  |  |  |  |  |  |  | 77.5 |
| 4' |  |  |  |  |  |  |  | 71.2 |
| 5' |  |  |  |  |  |  |  | 78.8 |
| 6' |  |  |  |  |  |  |  | 62.5 |
| 1'' |  |  |  |  |  |  | 5.62 (s) | 99.9 |
| 2'' |  |  |  |  |  |  |  | 72.0 |
| 3'' |  |  |  |  |  |  |  | 71.6 |
| 4'' |  |  |  |  |  |  |  | 73.5 |
| 5'' |  |  |  |  |  |  |  | 71.4 |
| 6'' |  |  |  |  |  |  | 1.27 (d, 6.1) | 18.2 |

**Table S11**. ^1^H (600 MHz, CD_3_OD-*d_4_*) and ^13^C NMR (151 MHz, CD_3_OD-*d_4_*) Data for **24**.

| **24** | | | | | |
| --- | --- | --- | --- | --- | --- |
| Position | ***δ*_H_** | ***δ_C_*** | Position | ***δ*_H_** | ***δ_C_*** |
| 2-COCH_3_ |  | 208.1 | 6-OCH_3_ | 3.87 (s) | 56.0 |
| 2-COCH_3_ | 2.59 (s) | 32.6 | 7 | 7.01 (d, 2.1) | 104.3 |
| 1 |  | 160.4 | 8 |  | 153.7 |
| 2 |  | 124.0 | 8a |  | 110.3 |
| 3 |  | 135.5 | 1' | 5.10 (d, 7.8) | 102.5 |
| 3-CH_3_ | 2.29 (s) | 20.2 | 2' | 3.62-3.39 (m) | 74.9 |
| 4 | 7.03(s) | 120.3 | 3' | 3.62-3.39 (m) | 78.8 |
| 4a |  | 139.2 | 4' | 3.62-3.39 (m) | 71.3 |
| 5 | 6.82 (d, 2.0) | 104.4 | 5' | 3.62-3.39 (m) | 78.1 |
| 6 |  | 157.2 | 6' | 3.74 (H-6'α, s)  3.95 (H-6'β, s) | 62.4 |

**Table S12**. ^1^H (600 MHz, CD_3_OD-*d_4_*) and ^13^C NMR (151 MHz, CD_3_OD-*d_4_*) Data for **25** and **26,** ^1^H (400 MHz, CD_3_OD-*d_4_*) and ^13^C NMR (101 MHz, CD_3_OD-*d_4_*) Data for **27**.

| Position | **25** | |  | **26** | |  | **27** | |
| --- | --- | --- | --- | --- | --- | --- | --- | --- |
|  | ***δ*_H_** | ***δ_C_*** |  | ***δ*_H_** | ***δ_C_*** |  | ***δ*_H_** | ***δ_C_*** |
| 1 |  | 140.0 |  |  | 121.7 |  |  | 120.2 |
| 2 | 6.79 (s) | 102.7 |  |  | 137.9 |  |  | 136.2 |
| 3 |  | 159.1 |  |  | 152.0 |  |  | 152.0 |
| 4 | 6.45 (t, 2.1) | 105.6 |  | 6.26 (d, 2.8) | 103.6 |  | 6.24 (d, 2.8) | 103.8 |
| 5 |  | 158.2 |  |  | 158.3 |  |  | 155.6 |
| 6 | 6.62 (s) | 101.0 |  | 6.63 (d, 2.8) | 108.2 |  | 6.53 (d, 2.8) | 104.2 |
| 7 | 6.85 (d, 16.3) | 125.3 |  | 6.93 (d, 16.5) | 133.7 |  | 6.85 (d, 16.3) | 131.2 |
| 8 | 7.02 (d, 16.3) | 128.6 |  | 7.71 (d, 16.5) | 130.8 |  | 7.15 (d, 16.3) | 130.1 |
| 1' |  | 128.9 |  |  | 130.1 |  |  | 133.4 |
| 2' | 7.37 (d, 8.6) | 127.5 |  | 7.46 (d, 8.6) | 129.21 |  | 7.11 (d, 8.6) | 129.0 |
| 3' | 6.77 (d, 8.6) | 115.1 |  | 6.78 (d, 8.6) | 116.4 |  | 6.66 (d, 8.6) | 116.5 |
| 4' |  | 157.1 |  |  | 155.9 |  |  | 158.2 |
| 5' | 6.77 (d, 8.6) | 115.1 |  | 6.78 (d, 8.6) | 116.4 |  | 6.66 (d, 8.6) | 116.5 |
| 6' | 7.37 (d, 8.6) | 127.5 |  | 7.46 (d, 8.6) | 129.2 |  | 7.11 (d, 8.6) | 129.0 |
| 1'' |  | 101.0 |  | 4.52 (d, 7.9) | 102.7 |  | 4.8 (d, 7.9) | 103.5 |
| 2'' |  | 73.6 |  |  | 75.5 |  |  | 76.2 |
| 3'' |  | 76.8 |  |  | 78.2 |  |  | 75.8 |
| 4'' |  | 70.1 |  |  | 70.8 |  |  | 71.3 |
| 5'' |  | 76.7 |  |  | 77.9 |  |  | 78.2 |
| 6'' |  | 61.2 |  |  | 62.1 |  |  | 62.1 |
| Galloyl  1''' |  |  |  |  |  |  |  | 121.7 |
| 2''' |  |  |  |  |  |  |  | 110.7 |
| 3''' |  |  |  |  |  |  | 7.19 (d, 8.4) | 146.5 |
| 4''' |  |  |  |  |  |  |  | 139.9 |
| 5''' |  |  |  |  |  |  |  | 146.5 |
| 6''' |  |  |  |  |  |  | 7.19 (d, 8.4) | 110.7 |
| COO |  |  |  |  |  |  |  | 168.0 |

**Table S13**. ^1^H (400 MHz, CD_3_OD-*d_4_*) and ^13^C NMR (101 MHz, CD_3_OD-*d_4_*) Data for **28** and **29**.

| Position | **28** | | **29** | |
| --- | --- | --- | --- | --- |
|  | ***δ*_H_** | ***δ_C_*** | ***δ*_H_** | ***δ_C_*** |
| CO |  | 170.2 |  | 170.4 |
| 1 |  | 123.1 |  | 122.1 |
| 2 |  | 115.8 | 7.06 (s) | 110.3 |
| 3 |  | 146.0 |  | 146.4 |
| 4 |  | 151.5 |  | 139.6 |
| 5 | 6.78 (d, 8.3) | 117.7 |  | 146.4 |
| 6 |  | 123.9 | 7.06 (s) | 110.3 |

**Compound 1**


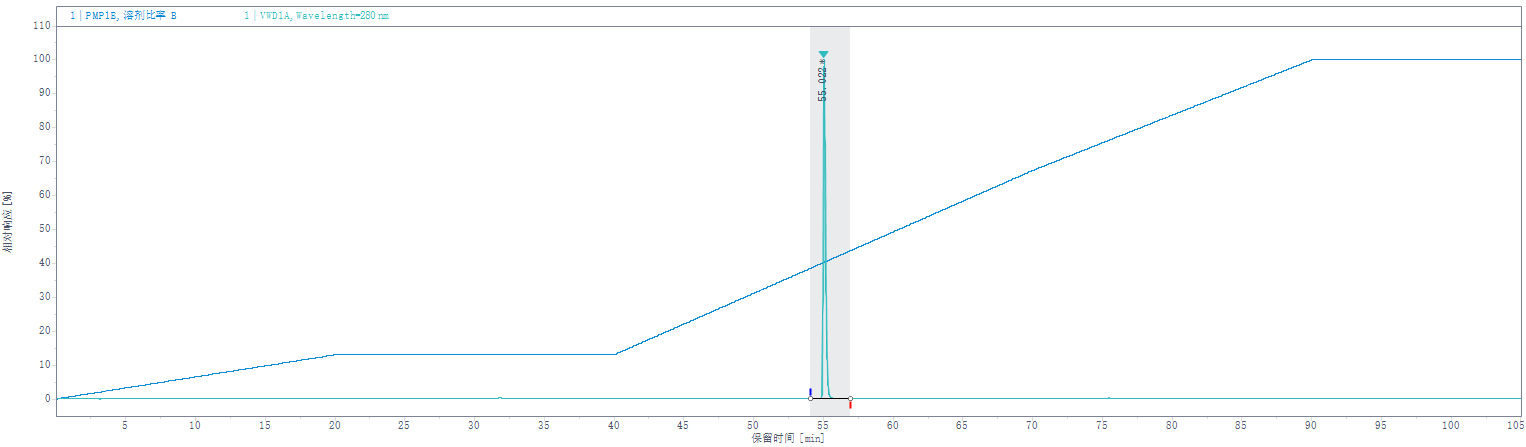


0-20min，0% MeOH-12% MeOH; 20-40min, 12% MeOH; 40-90min, 12% MeOH-100% MeOH

95-105min, 100% MeOH; Agilent 1120 (5 μm, 10 × 250 mm, Welch Ultimate XB-C18)

**Compound 2**


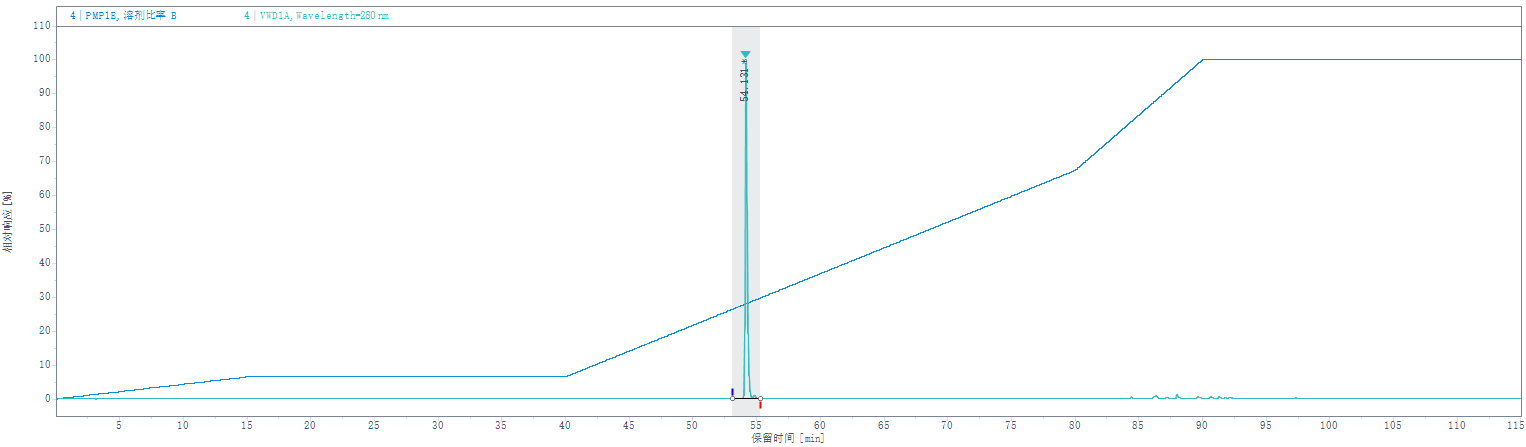


0-20min，0% MeOH-12% MeOH; 20-40min, 12% MeOH; 40-90min, 12% MeOH-100% MeOH

95-105min, 100% MeOH; Agilent 1120 (5 μm, 10 × 250 mm, Welch Ultimate XB-C18)

**Compound 3**


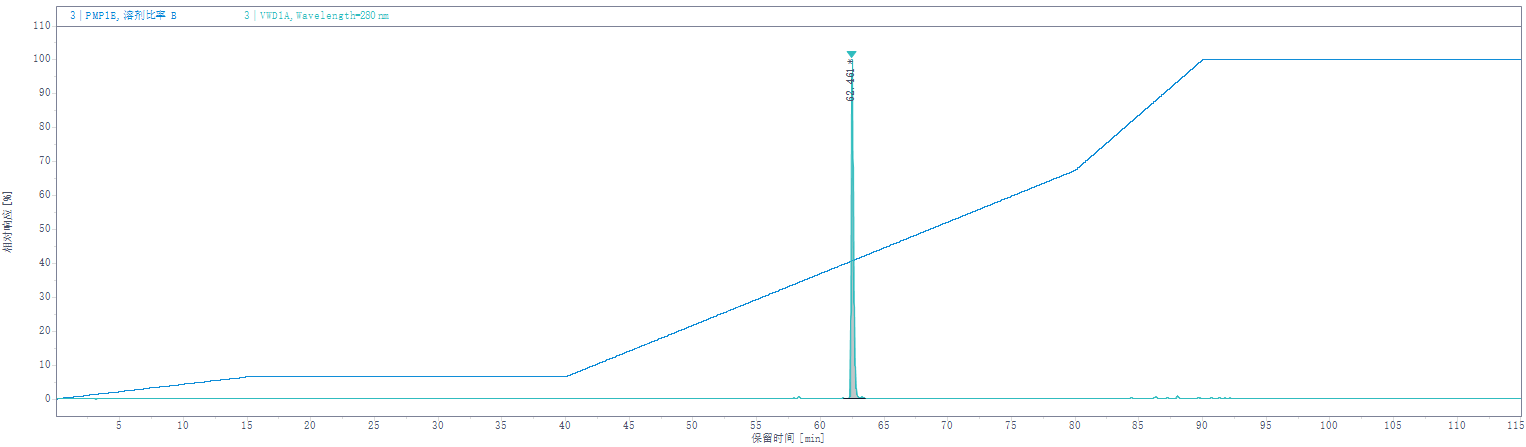


0-20min，0% MeOH-12% MeOH; 20-40min, 12% MeOH; 40-90min, 12% MeOH-100% MeOH

95-105min, 100% MeOH; Agilent 1120 (5 μm, 10 × 250 mm, Welch Ultimate XB-C18)

**Compoud 4**


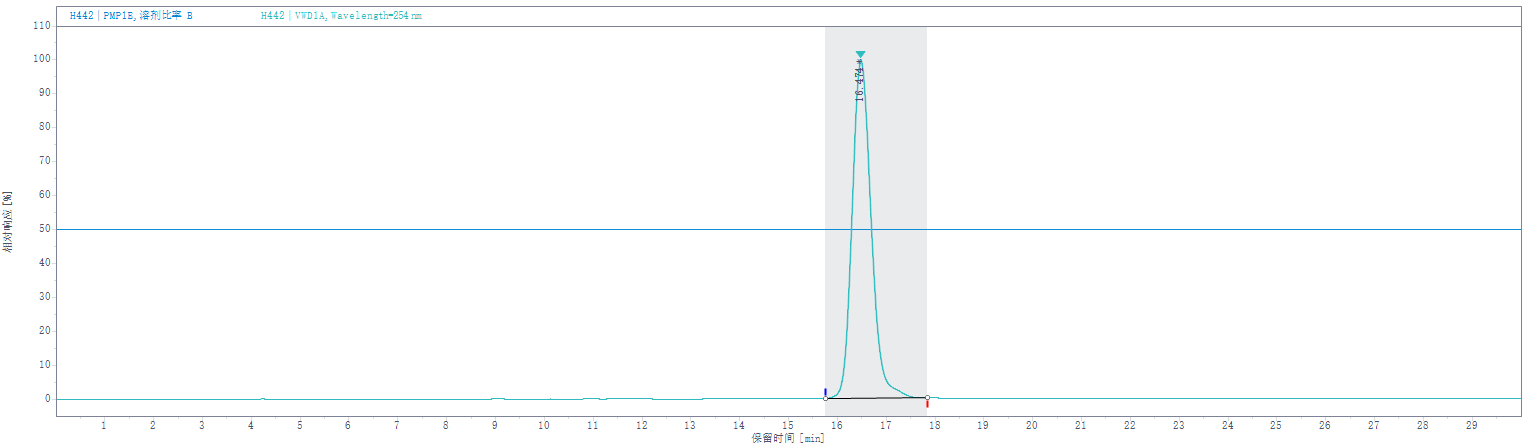


0-20min，5% MeOH; Agilent 1120 (5 μm, 10 × 250 mm, Welch Ultimate XB-C18)

**Compound 5**


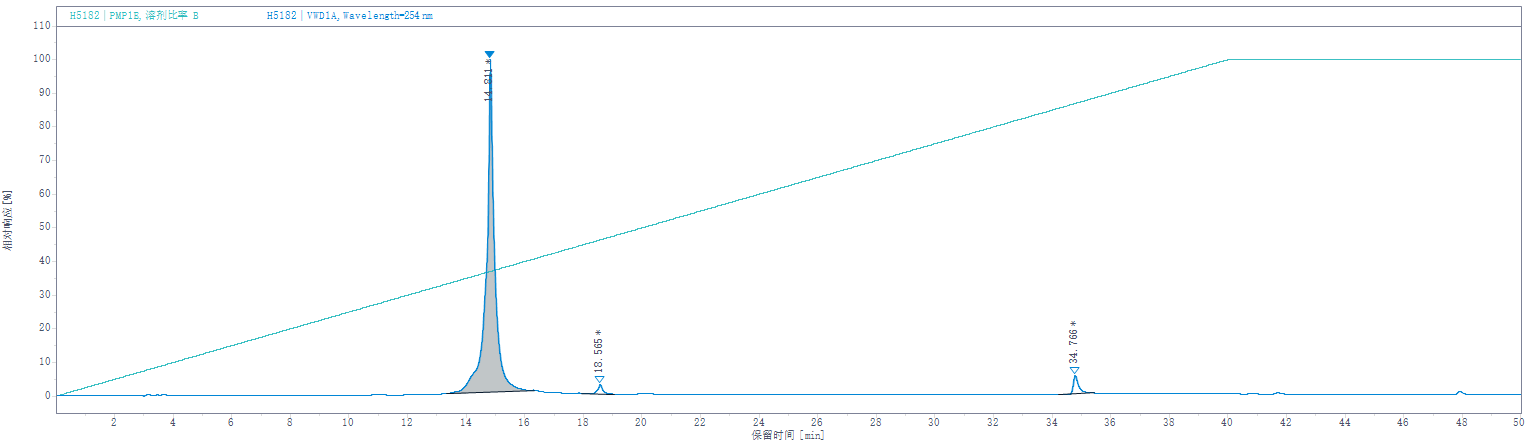


0-40min，10% MeOH-100%; Agilent 1120 (5 μm, 10 × 250 mm, Welch Ultimate XB-C18)

**Compound 6**


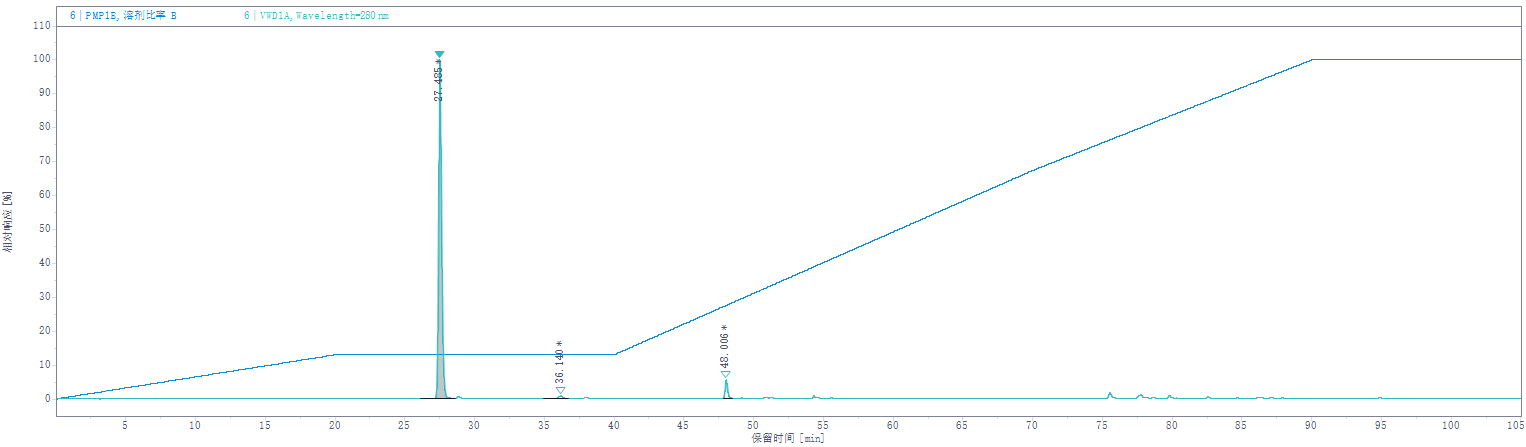


0-20min，0% MeOH-12% MeOH; 20-40min, 12% MeOH; 40-90min, 12% MeOH-100% MeOH

95-105min, 100% MeOH; Agilent 1120 (5 μm, 10 × 250 mm, Welch Ultimate XB-C18)

**Compound 7**


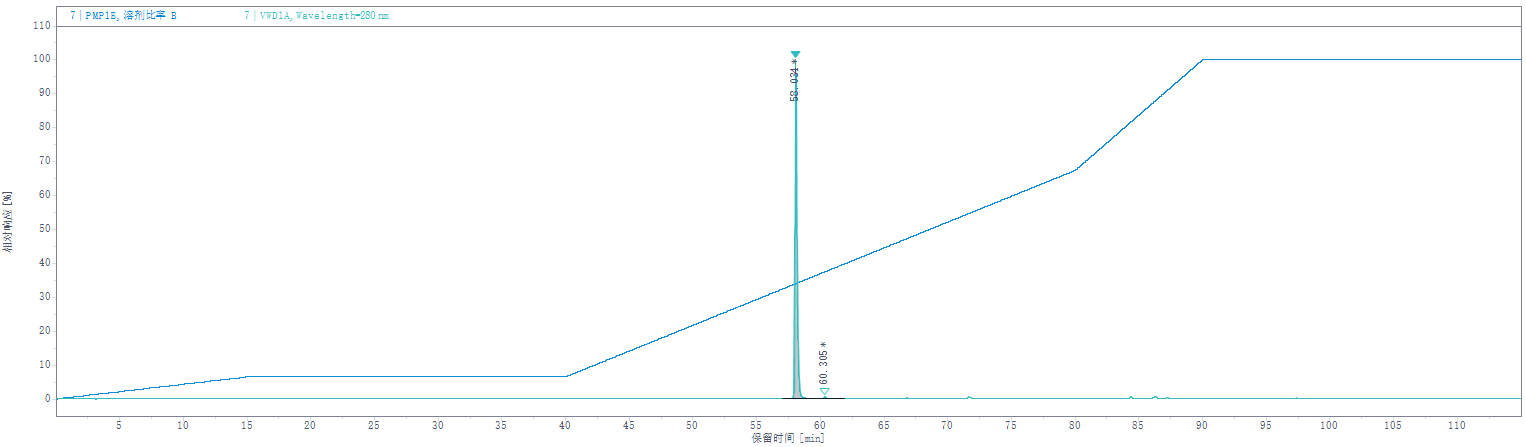


0-15min，0% MeOH-8% MeOH; 15-40min, 8% MeOH; 40-80min, 8% MeOH-70% MeOH

80-90min, 70% MeOH-100% MeOH; Agilent 1120 (5 μm, 10 × 250 mm, Welch Ultimate XB-C18)

**Compound 8**


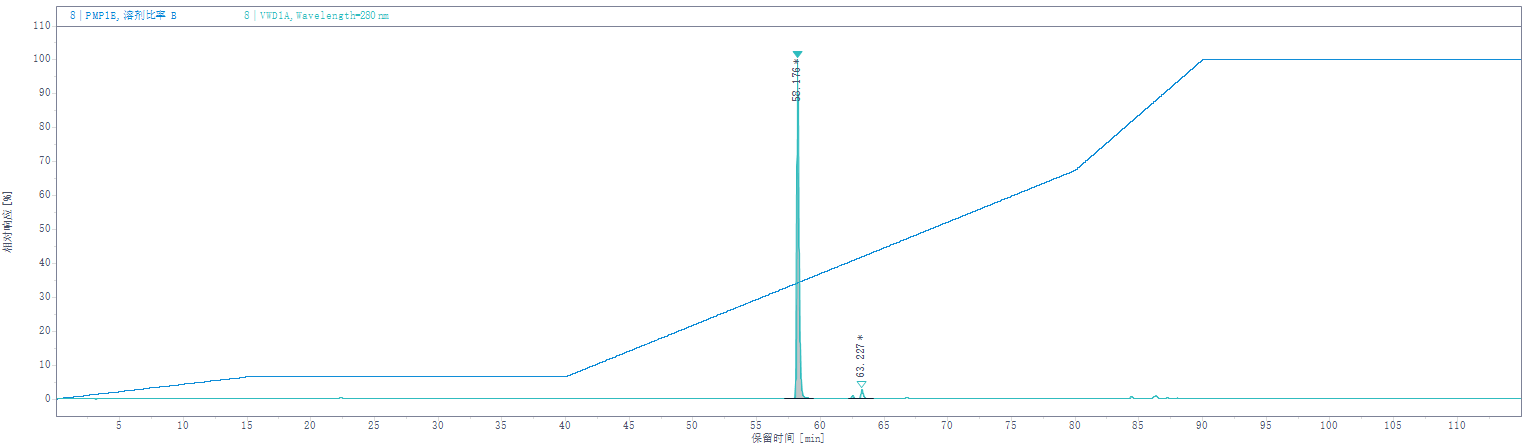


0-15min，0% MeOH-8% MeOH; 15-40min, 8% MeOH; 40-80min, 8% MeOH-70% MeOH

80-90min, 70% MeOH-100% MeOH; Agilent 1120 (5 μm, 10 × 250 mm, Welch Ultimate XB-C18)

**Compound 9**


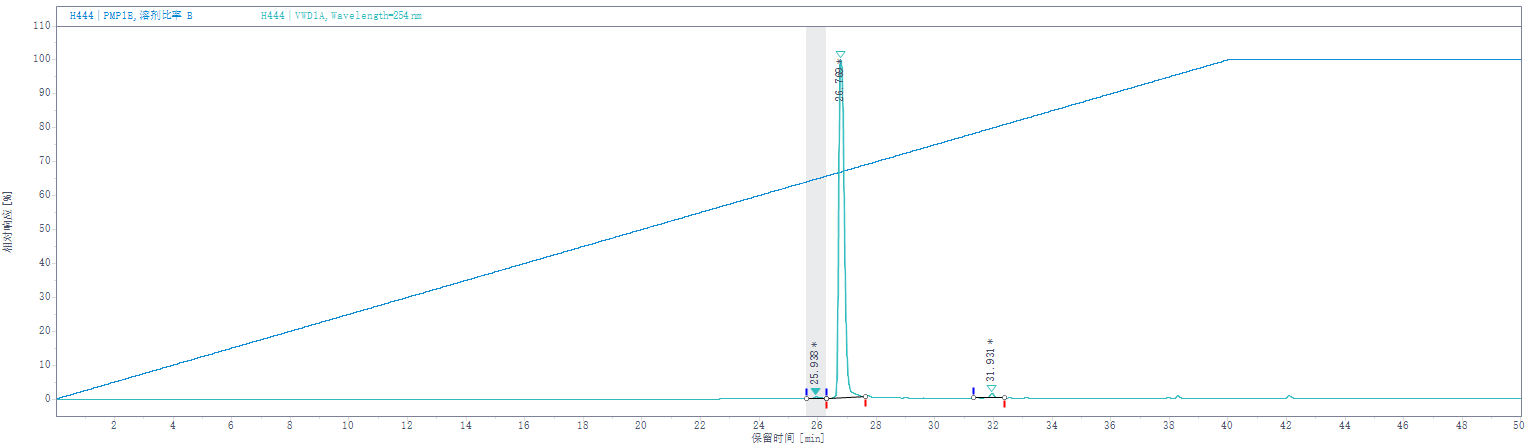


0-40min，0% MeOH-100% MeOH; 40-50min, 100% MeOH; Agilent 1120 (5 μm, 10 × 250 mm, Welch Ultimate XB-C18)

**化合物10**


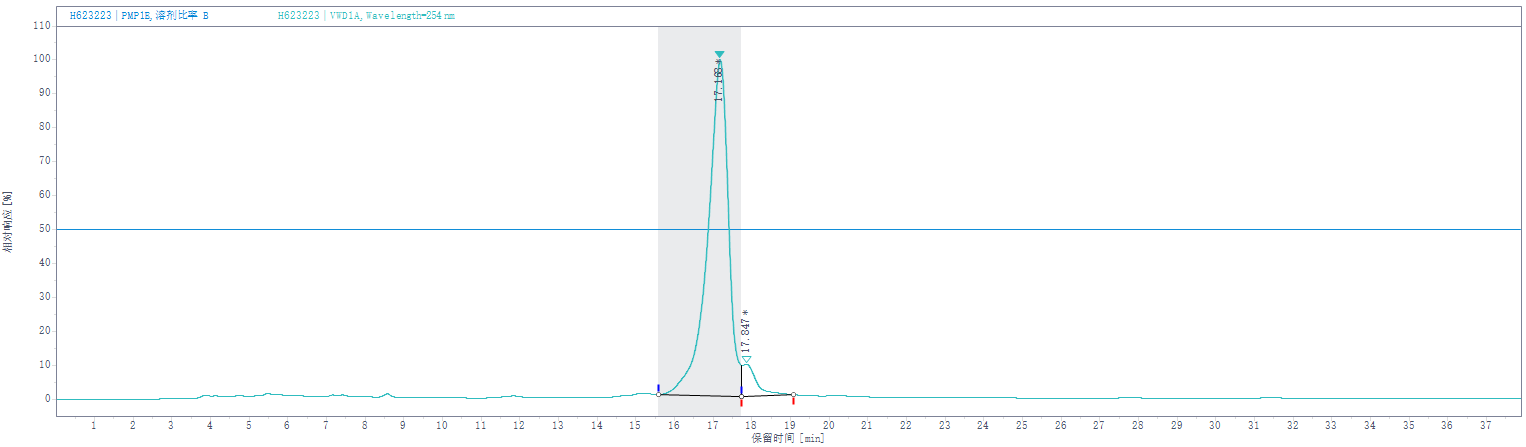


0-30min，50% MeOH; Agilent 1120 (5 μm, 10 × 250 mm, Welch Ultimate XB-C18)

**Compound 11**


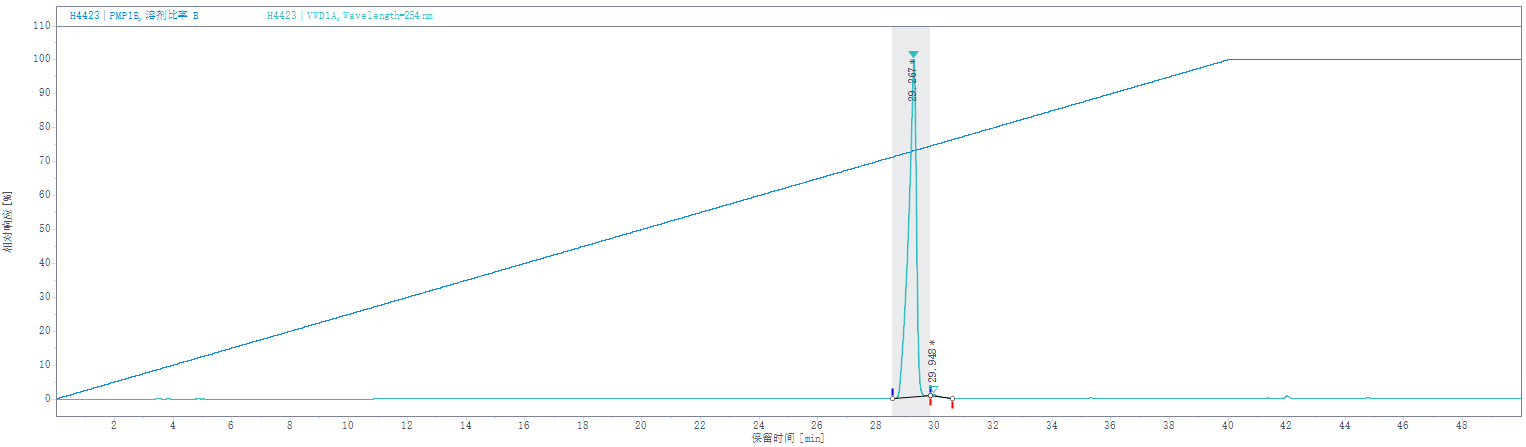


0-40min，0% MeOH-100% MeOH; 40-50min, 100% MeOH; Agilent 1120 (5 μm, 10 × 250 mm, Welch Ultimate XB-C18)

**Compound 12**


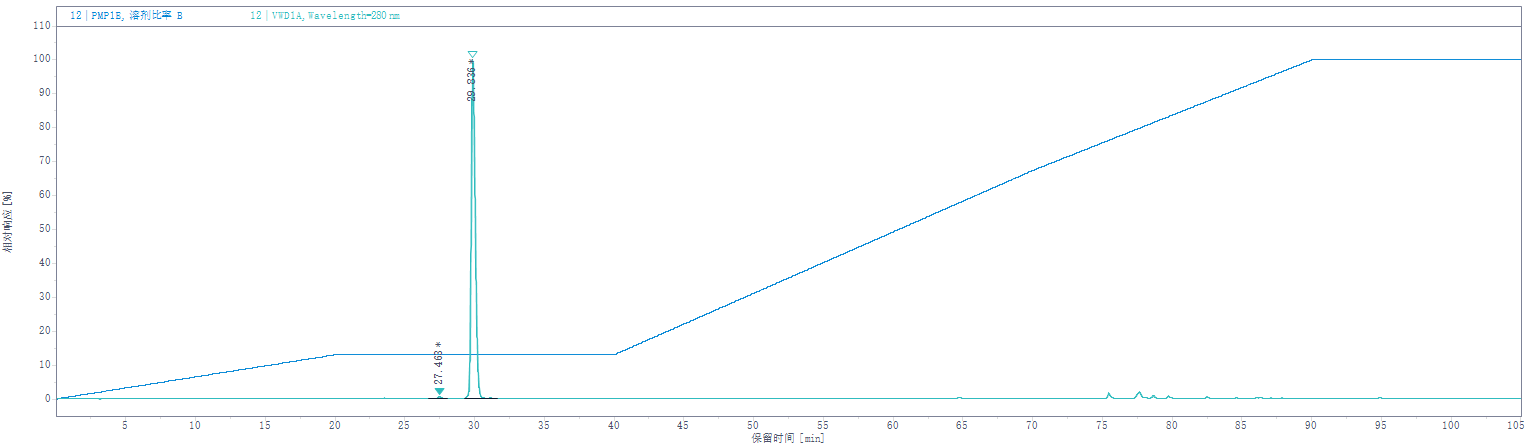


0-20min，0% MeOH-12% MeOH; 20-40min, 12% MeOH; 40-90min, 12% MeOH-100% MeOH; Agilent 1120 (5 μm, 10 × 250 mm, Welch Ultimate XB-C18)

**Compound 13**


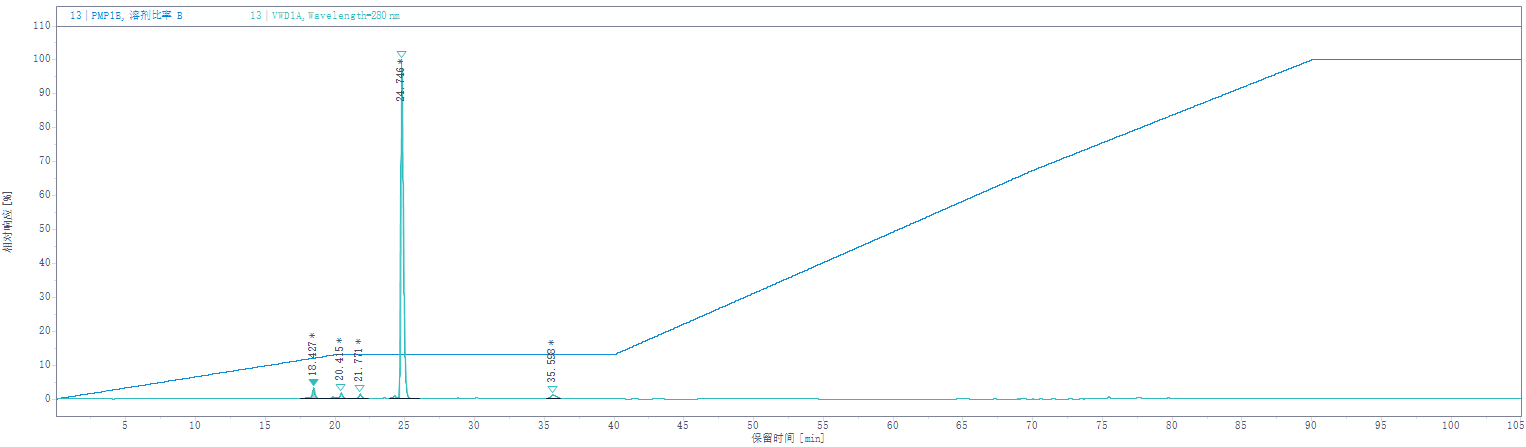


0-15min，0% MeOH-8% MeOH; 15-40min, 8% MeOH; 40-80min, 8% MeOH-70% MeOH

80-90min, 70% MeOH-100% MeOH; Agilent 1120 (5 μm, 10 × 250 mm, Welch Ultimate XB-C18)

**Compoud 16**


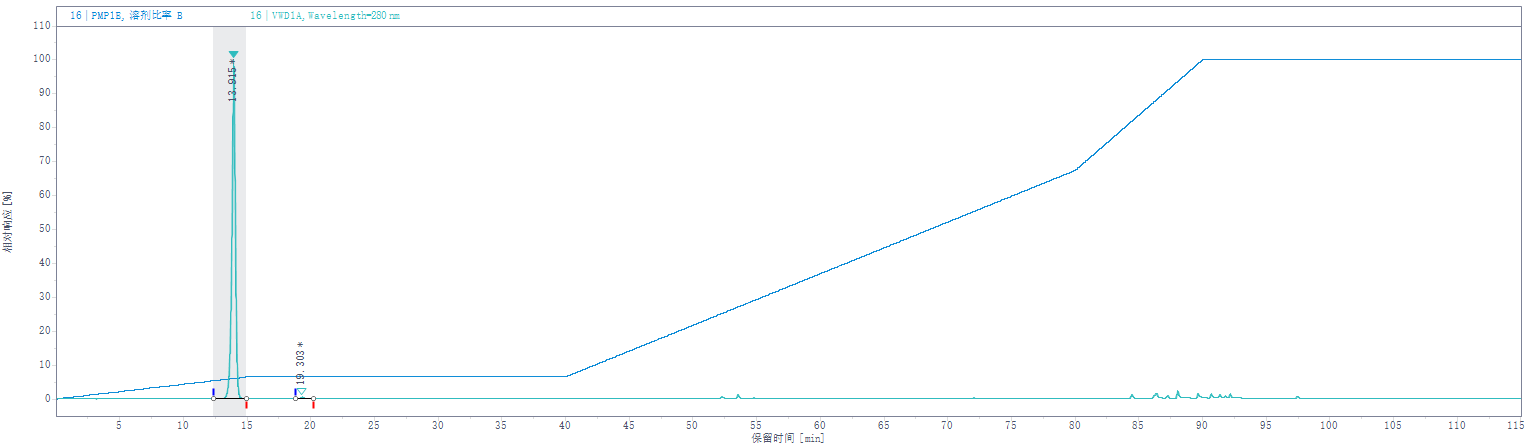


0-15min，0% MeOH-8% MeOH; 15-40min, 8% MeOH; 40-80min, 8% MeOH-70% MeOH

80-90min, 70% MeOH-100% MeOH; Agilent 1120 (5 μm, 10 × 250 mm, Welch Ultimate XB-C18)

**Compound 18**


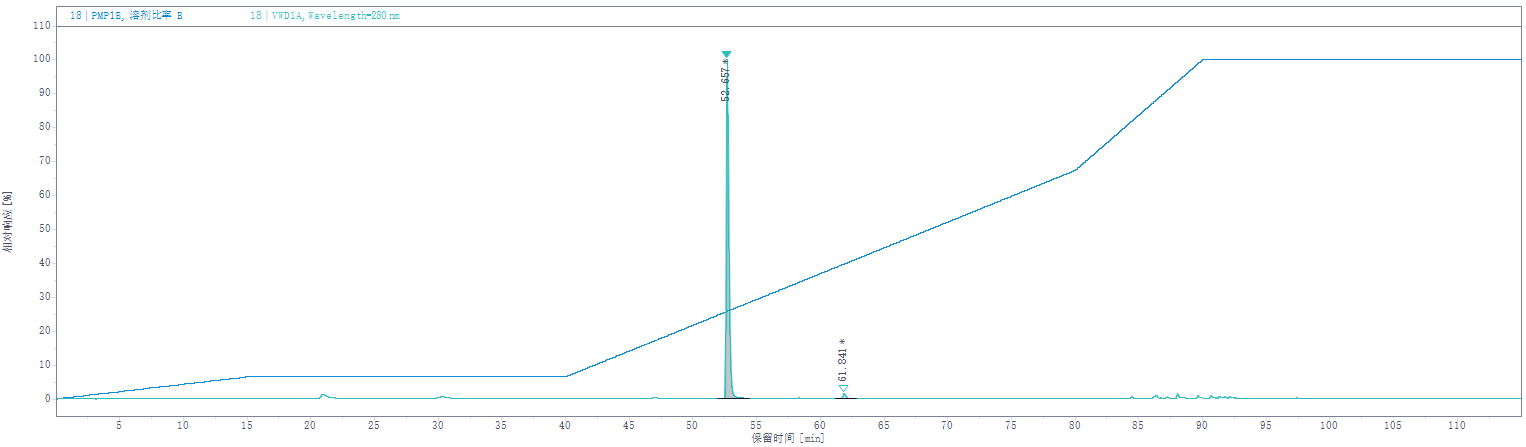


0-15min，0% MeOH-8% MeOH; 15-40min, 8% MeOH; 40-80min, 8% MeOH-70% MeOH

80-90min, 70% MeOH-100% MeOH; Agilent 1120 (5 μm, 10 × 250 mm, Welch Ultimate XB-C18)

**Compound 20**


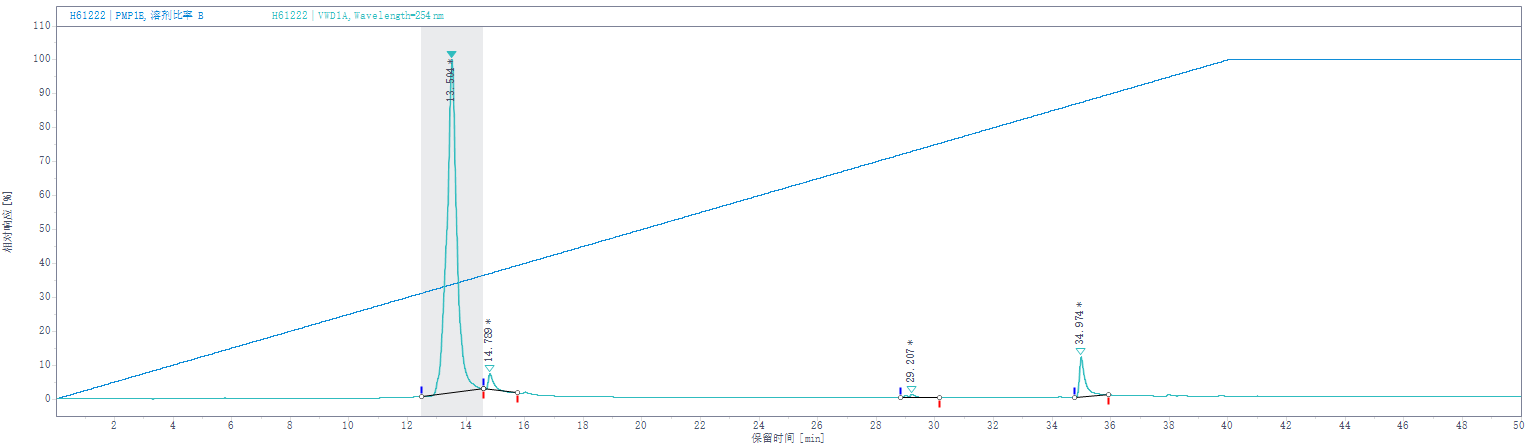


0-40min，0% MeOH-100% MeOH; 40-50min, 100% MeOH; Agilent 1120 (5 μm, 10 × 250 mm, Welch Ultimate XB-C18)

**Compound 21**


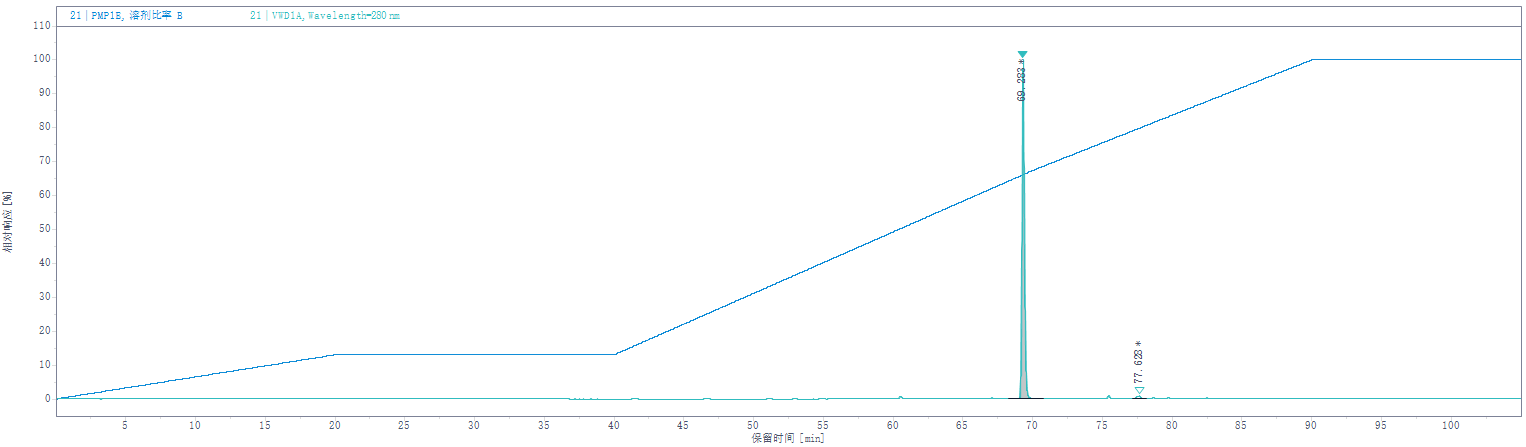


0-20min，0% MeOH-12% MeOH; 20-40min, 12% MeOH; 40-90min, 12% MeOH-100% MeOH

90-100min, 100% MeOH; Agilent 1120 (5 μm, 10 × 250 mm, Welch Ultimate XB-C18)

**Compound 22**


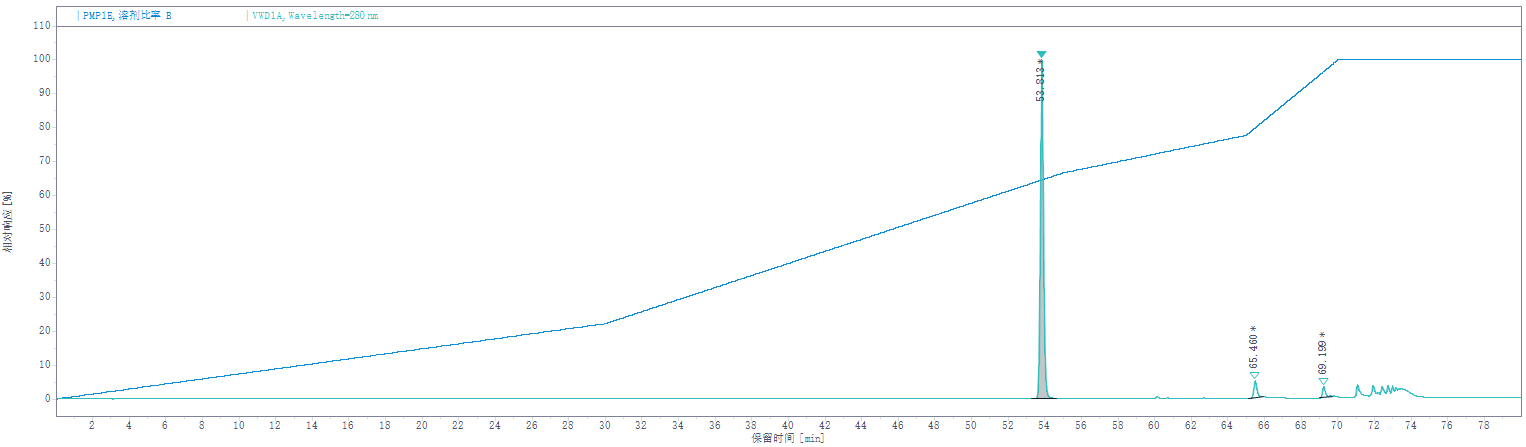


0-30min，0% MeOH-20% MeOH; 30-55min, 20% MeOH-60%; 55-65min MeOH, 60% MeOH-80% MeOH; 65-70min, 80% MeOH-100% MeOH; Agilent 1120 (5 μm, 10 × 250 mm, Welch Ultimate XB-C18)

**Compound 24**


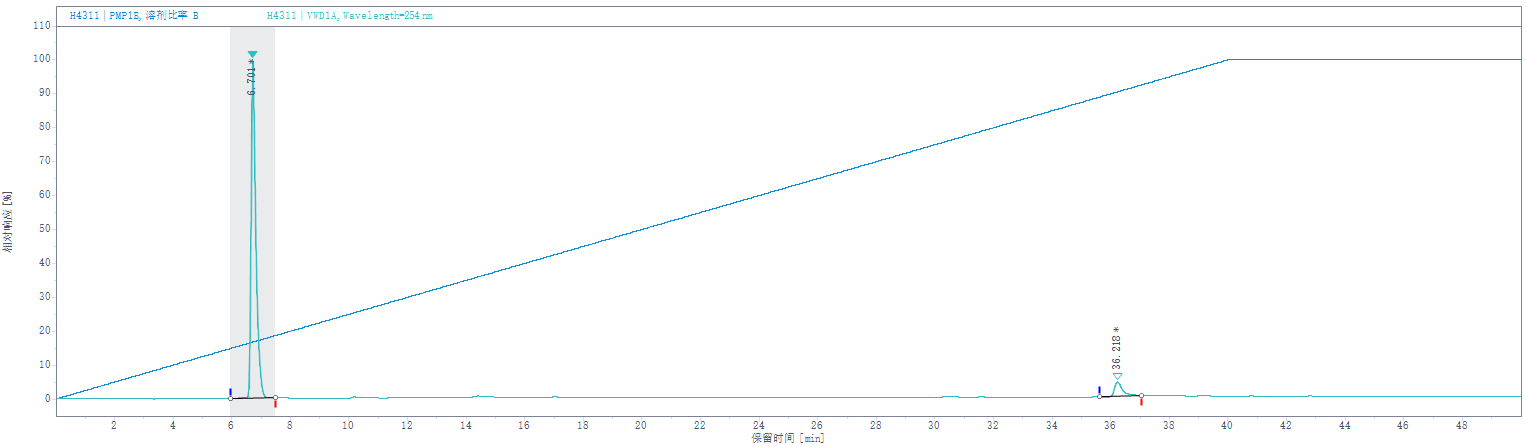


0-40min，0% MeOH-100% MeOH; 40-50min, 100% MeOH; Agilent 1120 (5 μm, 10 × 250 mm, Welch Ultimate XB-C18)

**Compound 26**


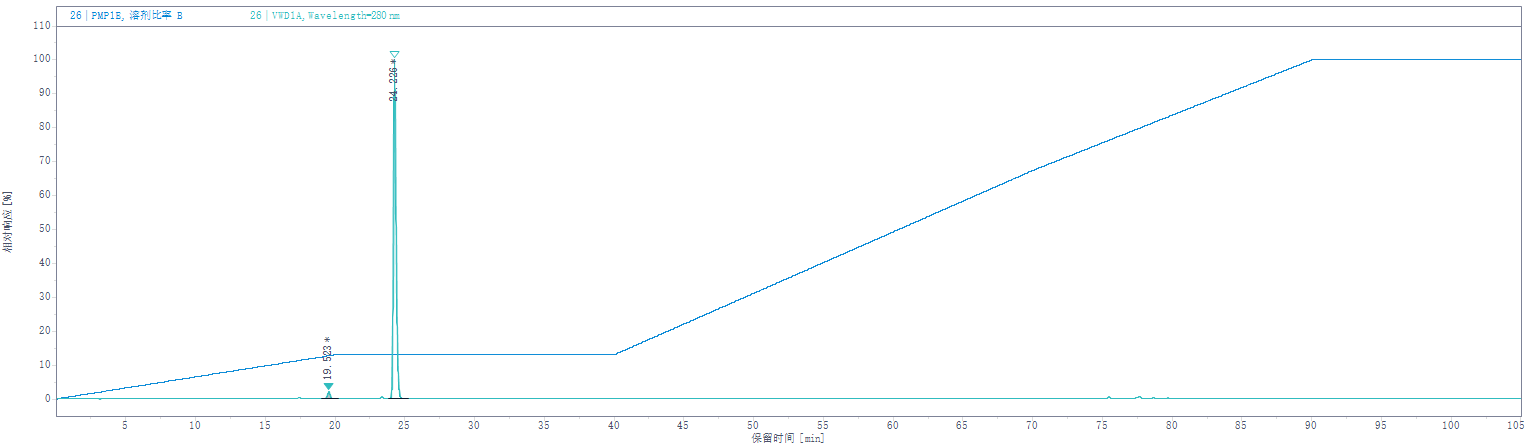


0-20min，0% MeOH-12% MeOH; 20-40min, 12% MeOH; 40-90min, 12% MeOH-100% MeOH

90-100min, 100% MeOH; Agilent 1120 (5 μm, 10 × 250 mm, Welch Ultimate XB-C18)

**Compound 31**


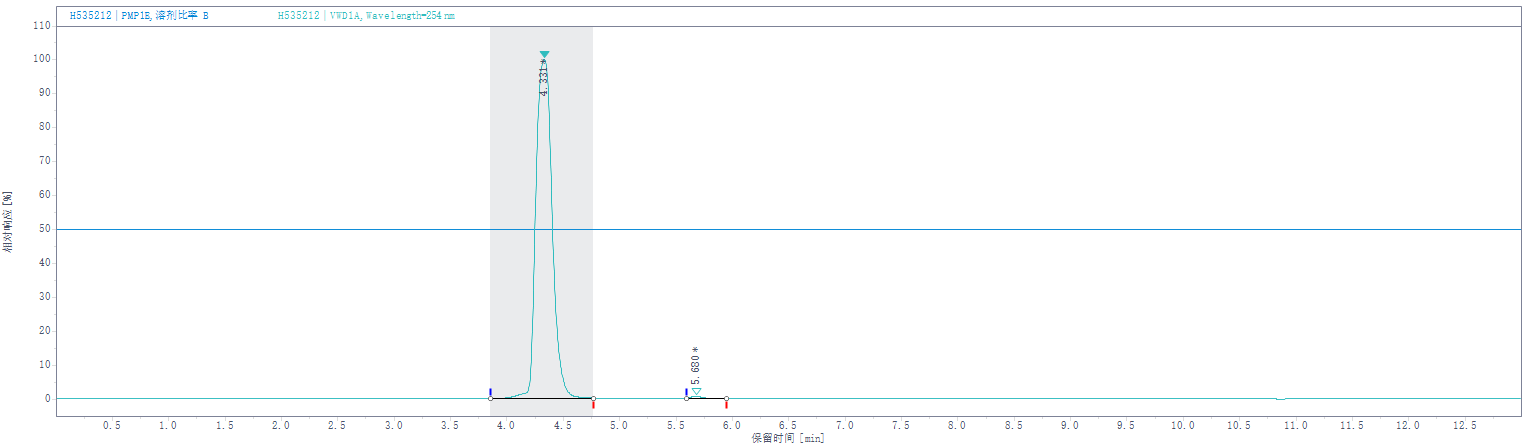


0-20min，50% MeOH; Agilent 1120 (5 μm, 10 × 250 mm, Welch Ultimate XB-C18)

**Compound 33**


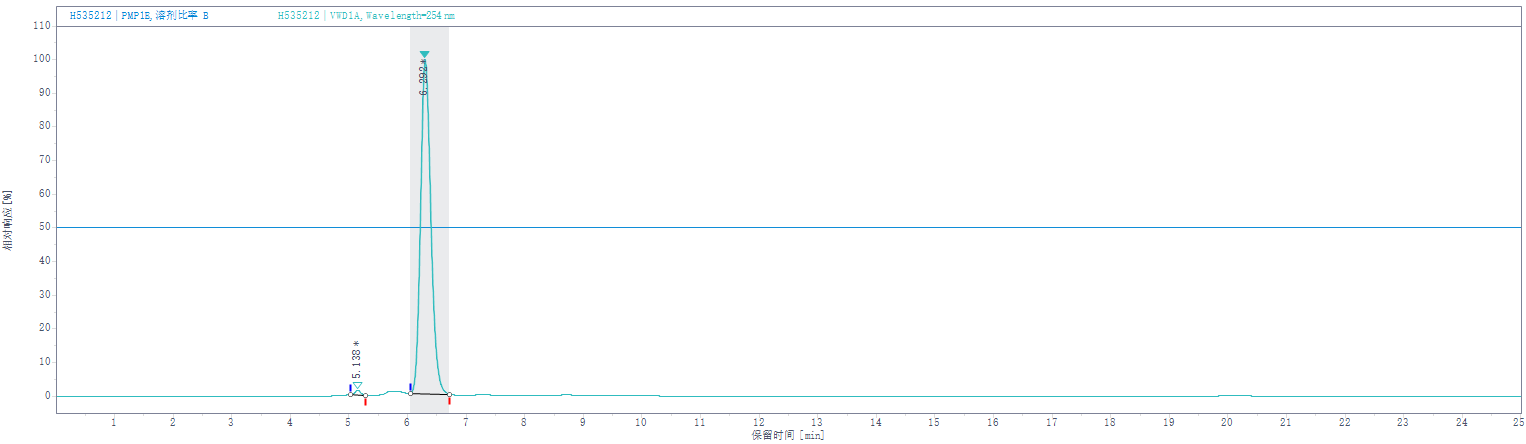


0-20min，50% MeOH; Agilent 1120 (5 μm, 10 × 250 mm, Welch Ultimate XB-C18)

**Compound 34**


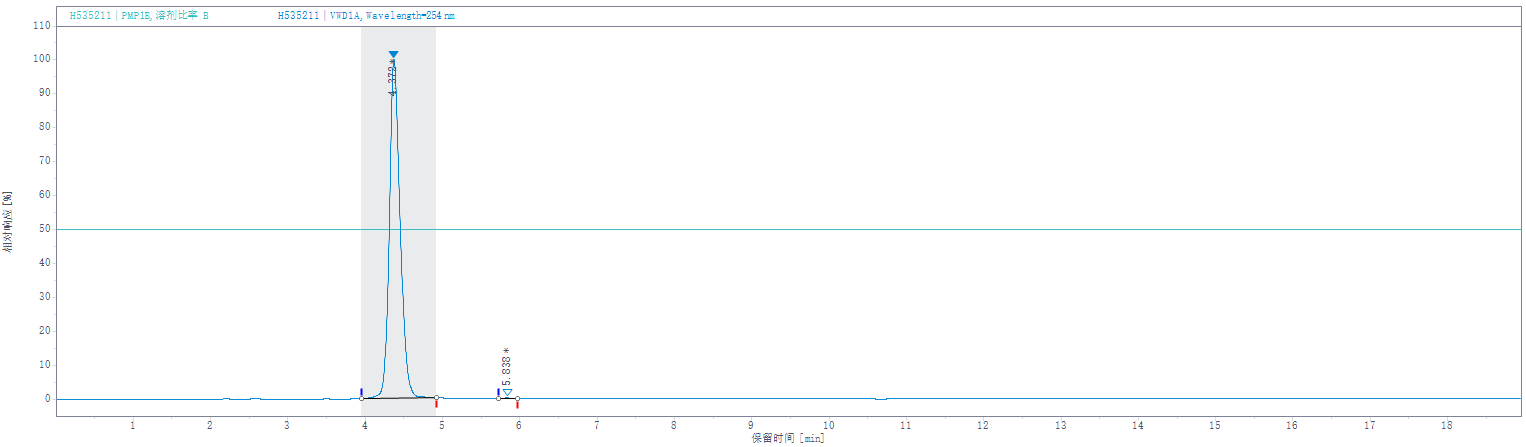


0-20min，50% MeOH; Agilent 1120 (5 μm, 10 × 250 mm, Welch Ultimate XB-C18)


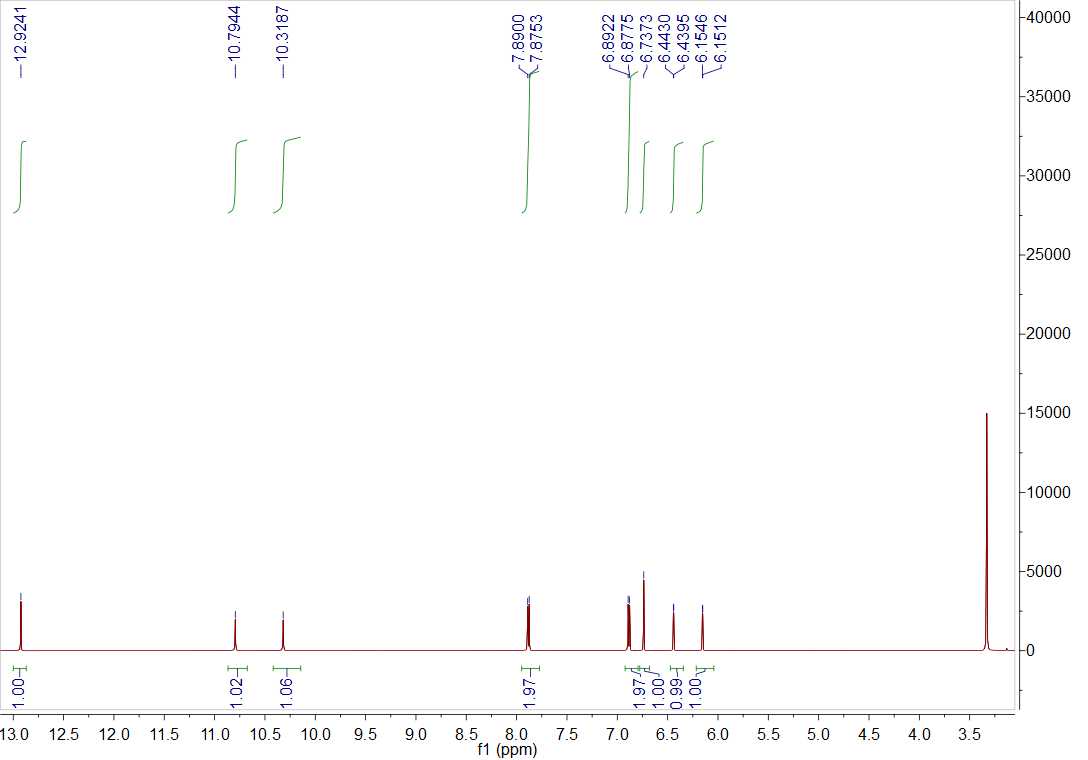
^1^H NMR spectrum (600 MHz) of compound **1** in DMSO-*d*_6_.

^13^C NMR spectrum (151 MHz) of compound **1** in DMSO-*d*_6_.


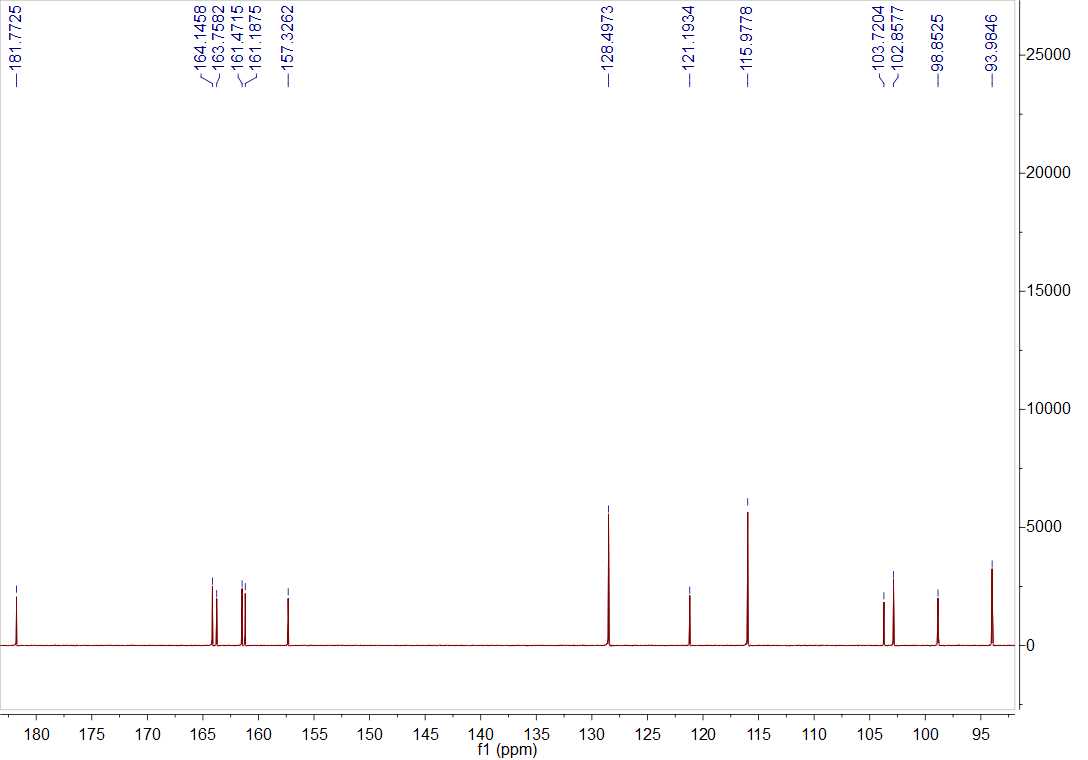


^1^H NMR spectrum (400 MHz) of compound **2** in DMSO-*d*_6_.


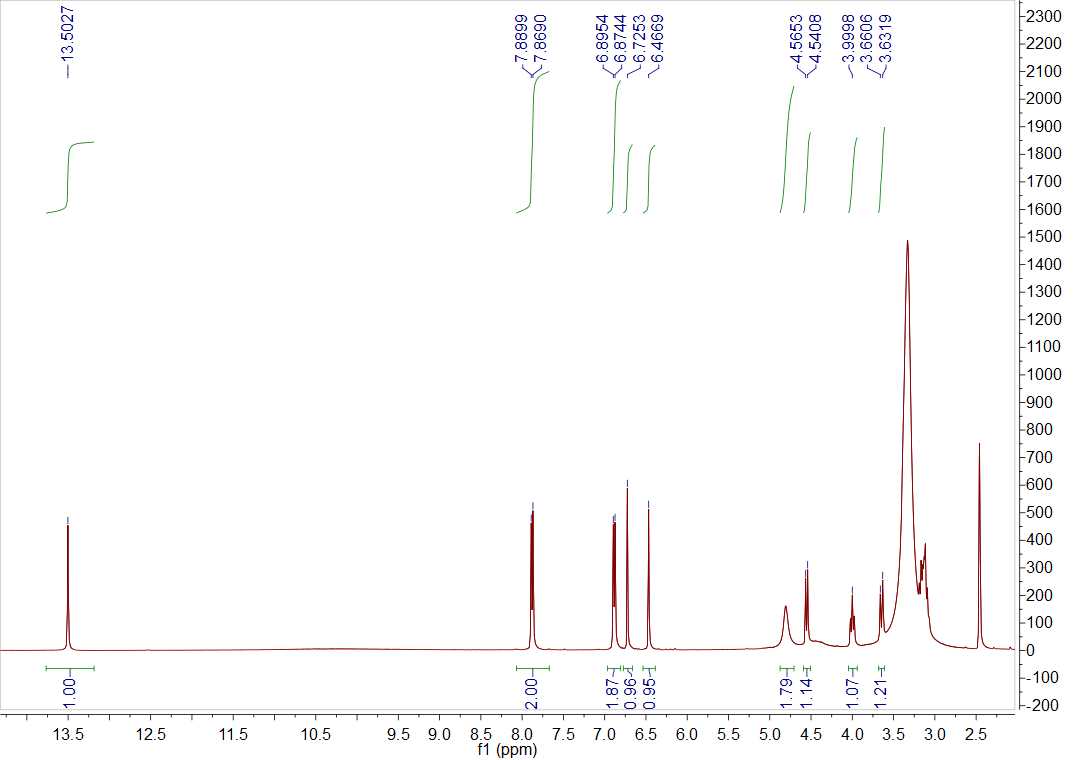


^13^C NMR spectrum (101 MHz) of compound **2** in DMSO-*d*_6_.


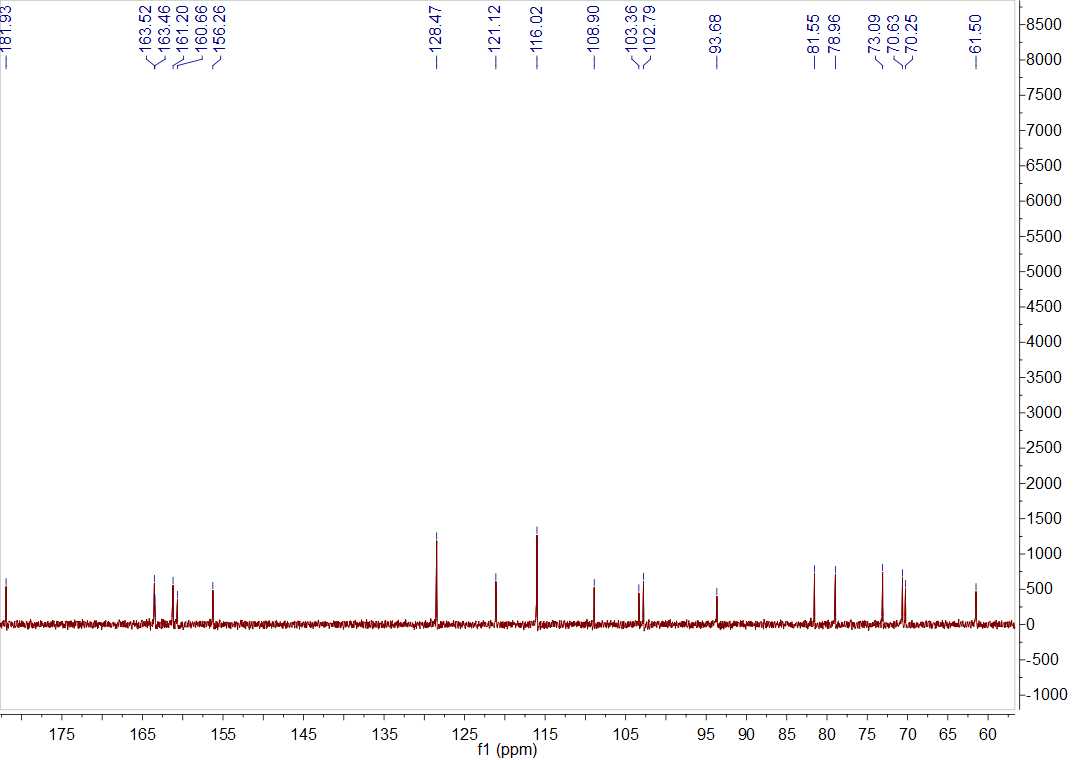


^1^H NMR spectrum (600 MHz) of compound **3** in DMSO-*d*_6_.


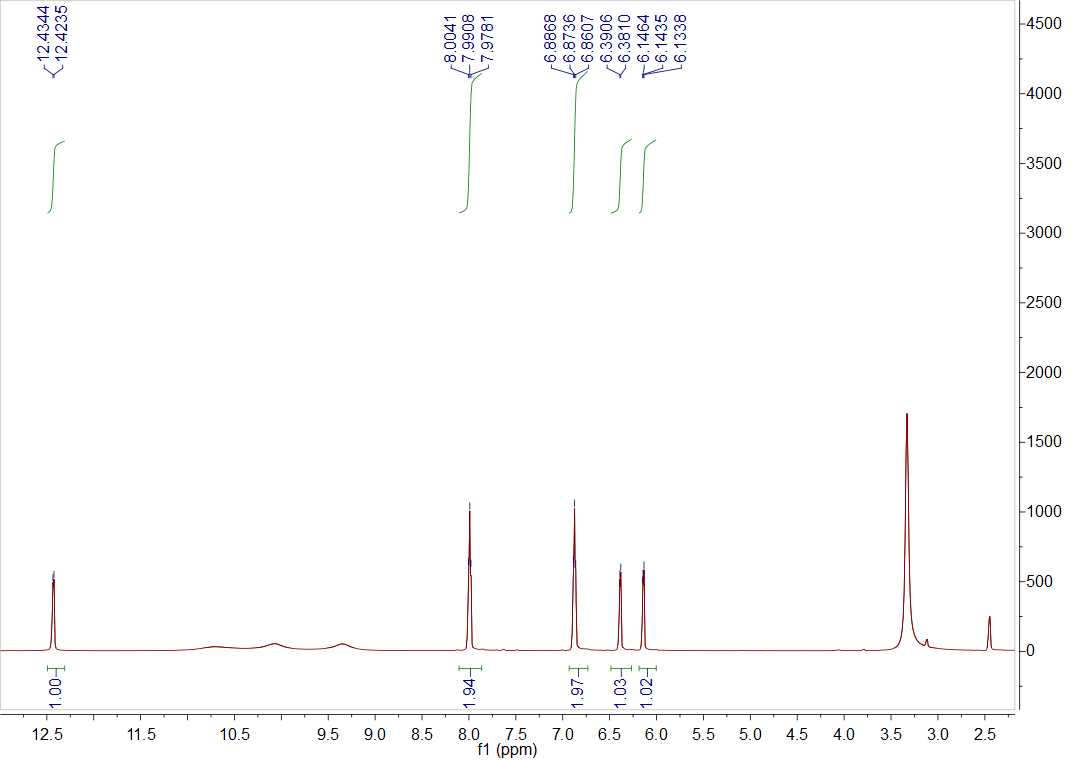


^13^C NMR spectrum (151 MHz) of compound **3** in DMSO-*d*_6_.


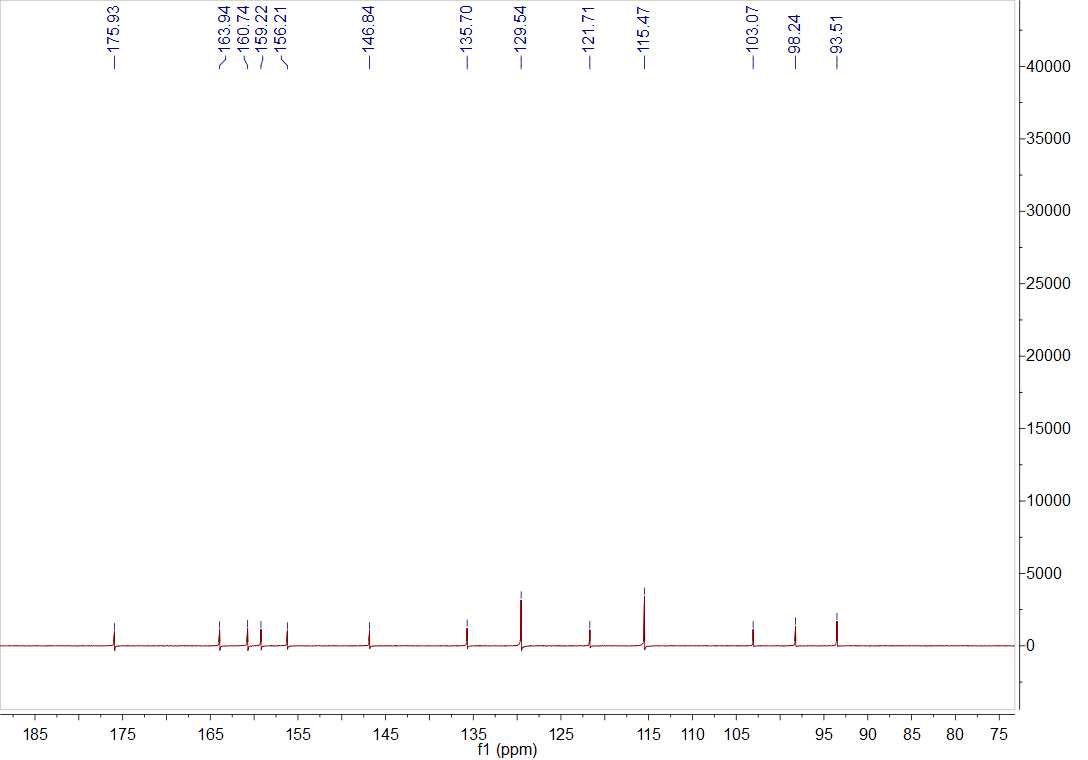


^1^H NMR spectrum (600 MHz) of compound **4** in CD_3_OD.


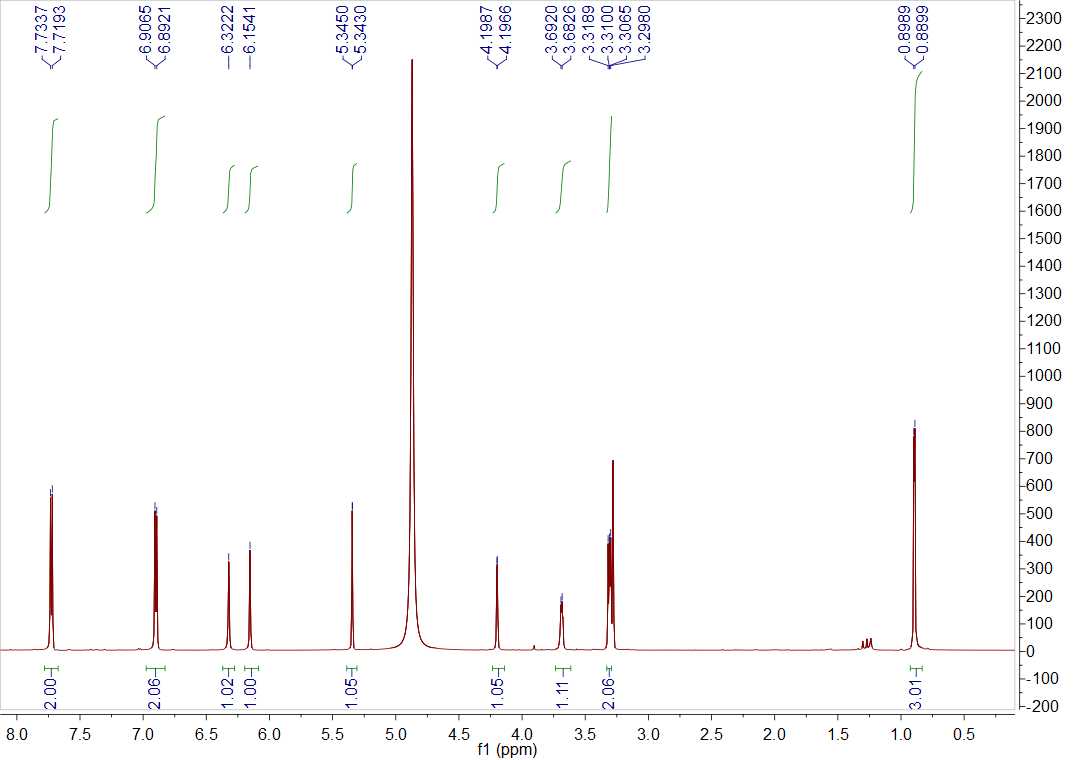


^13^C NMR spectrum (151 MHz) of compound **4** in CD_3_OD.


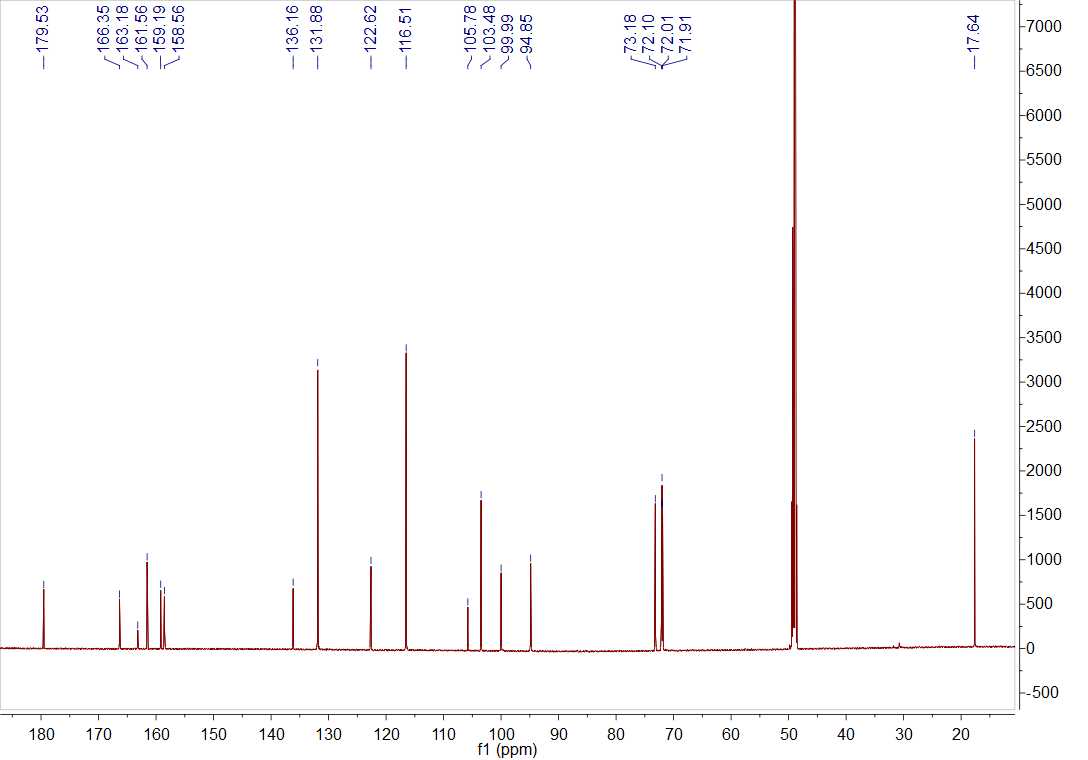


^1^H NMR spectrum (400 MHz) of compound **5** in DMSO-*d*_6_.


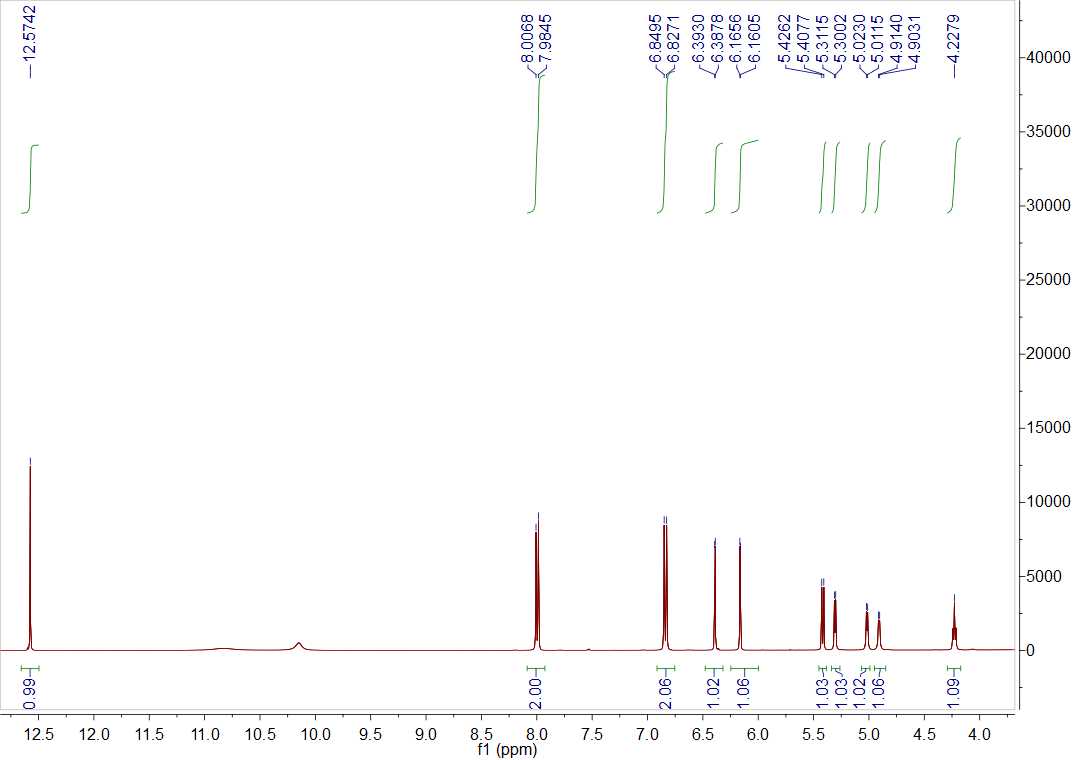


^13^C NMR spectrum (101 MHz) of compound **5** in DMSO-*d*_6_.


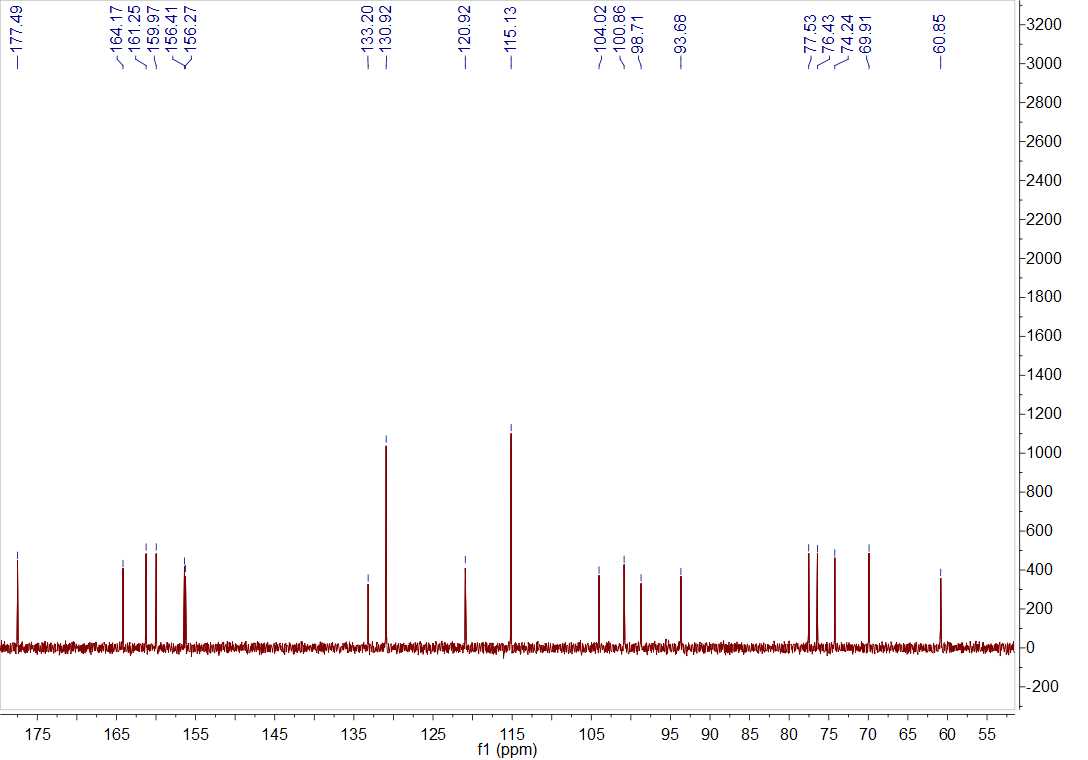


^1^H NMR spectrum (600 MHz) of compound **6** in CD_3_OD.


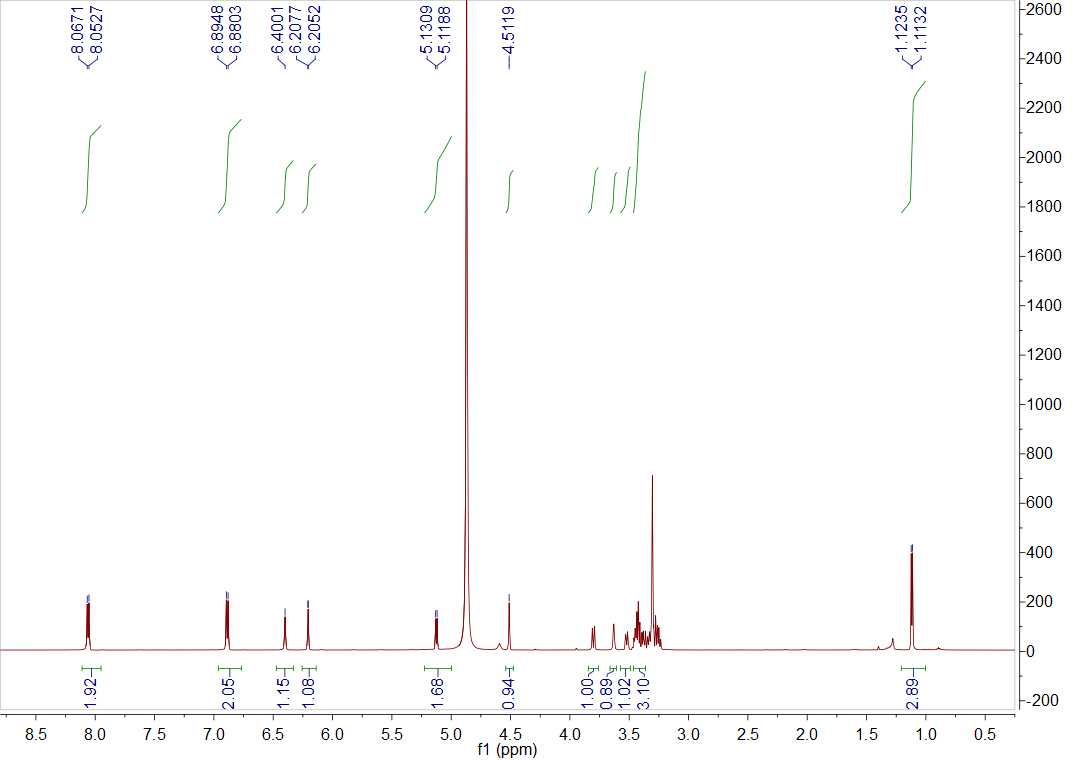


^13^C NMR spectrum (151 MHz) of compound **6** in CD_3_OD.


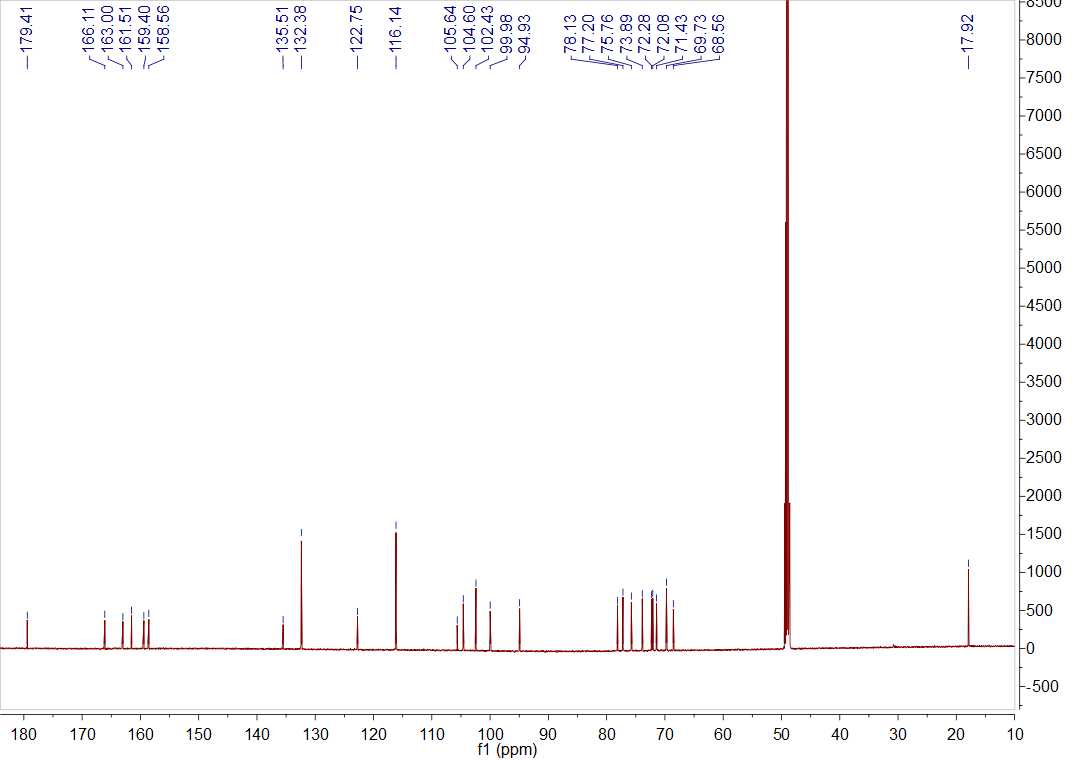


^1^H NMR spectrum (600 MHz) of compound **7** in DMSO-*d*_6_.


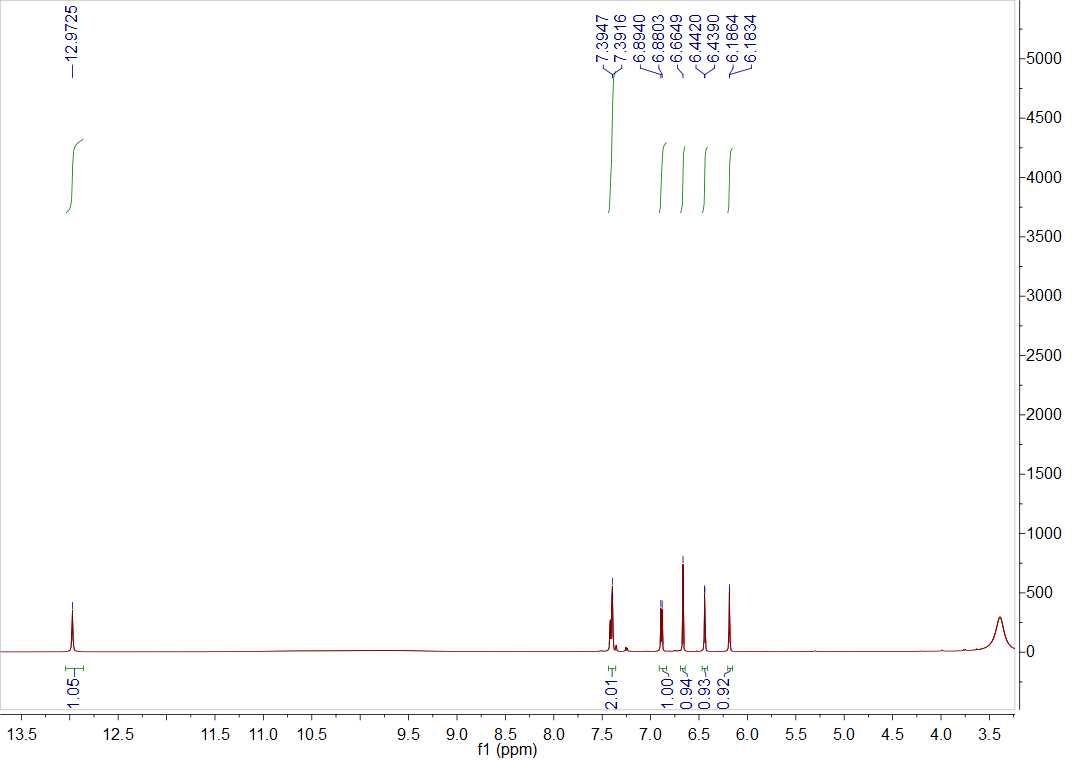


^13^C NMR spectrum (151 MHz) of compound **7** in DMSO-*d*_6_.


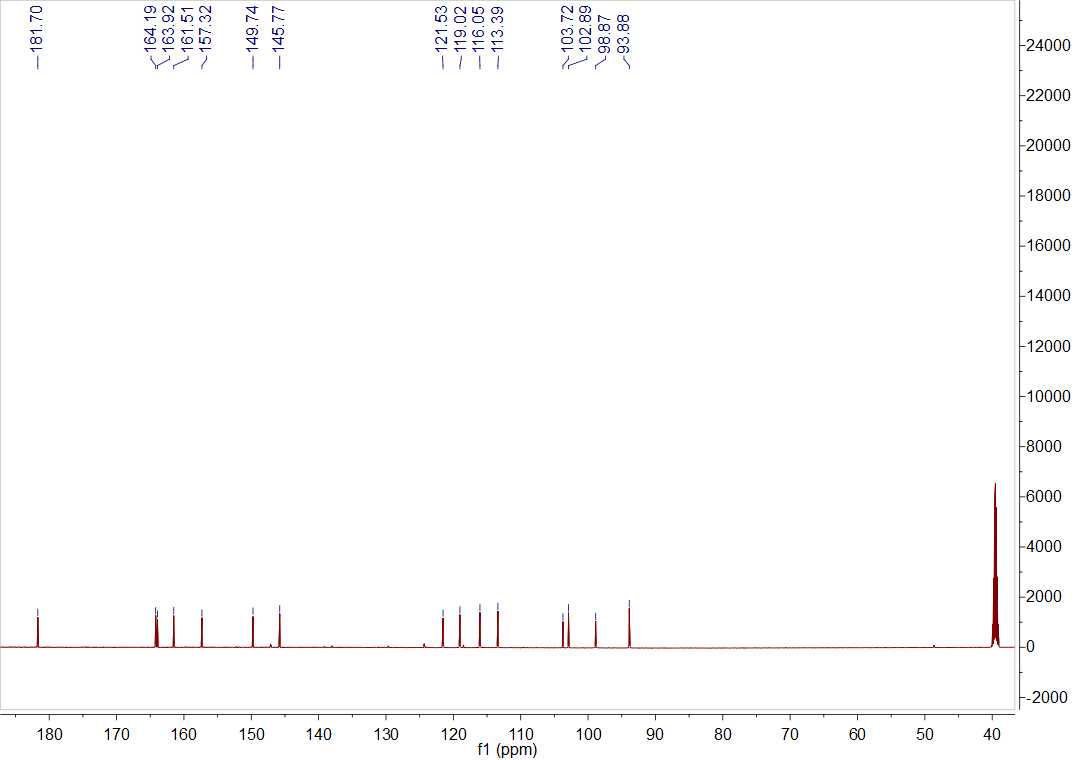


^1^H NMR spectrum (600 MHz) of compound **8** in DMSO-*d*_6_.


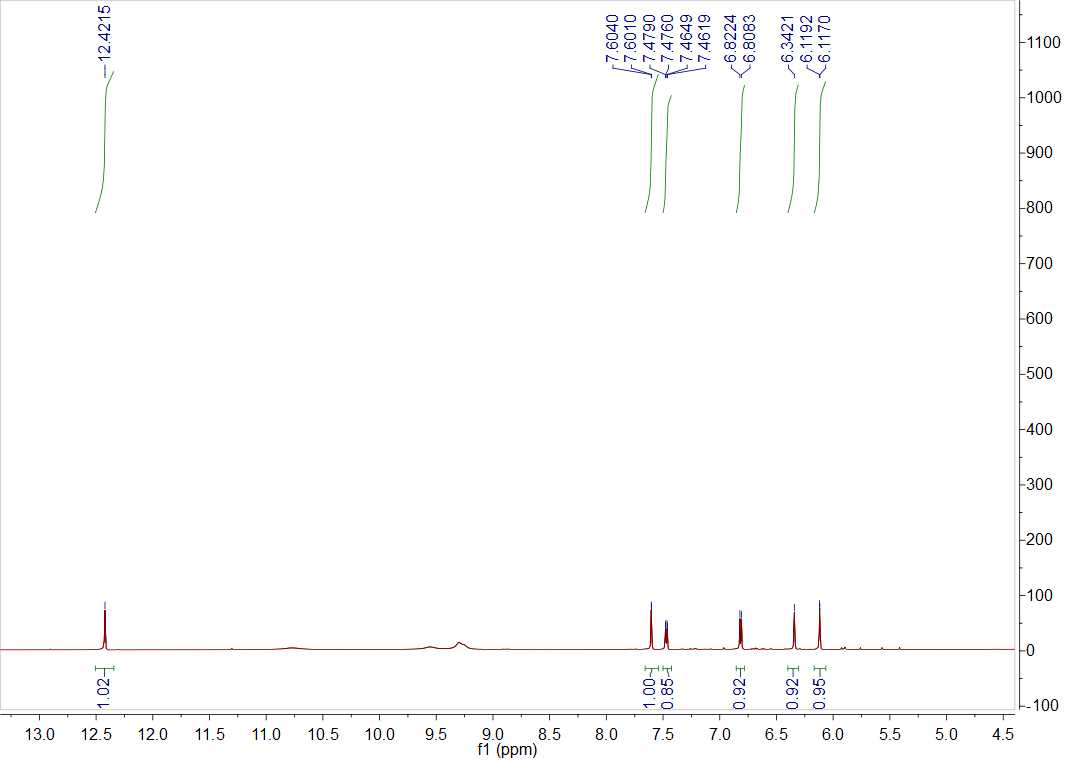


^13^C NMR spectrum (151 MHz) of compound **8** in DMSO-*d*_6_.


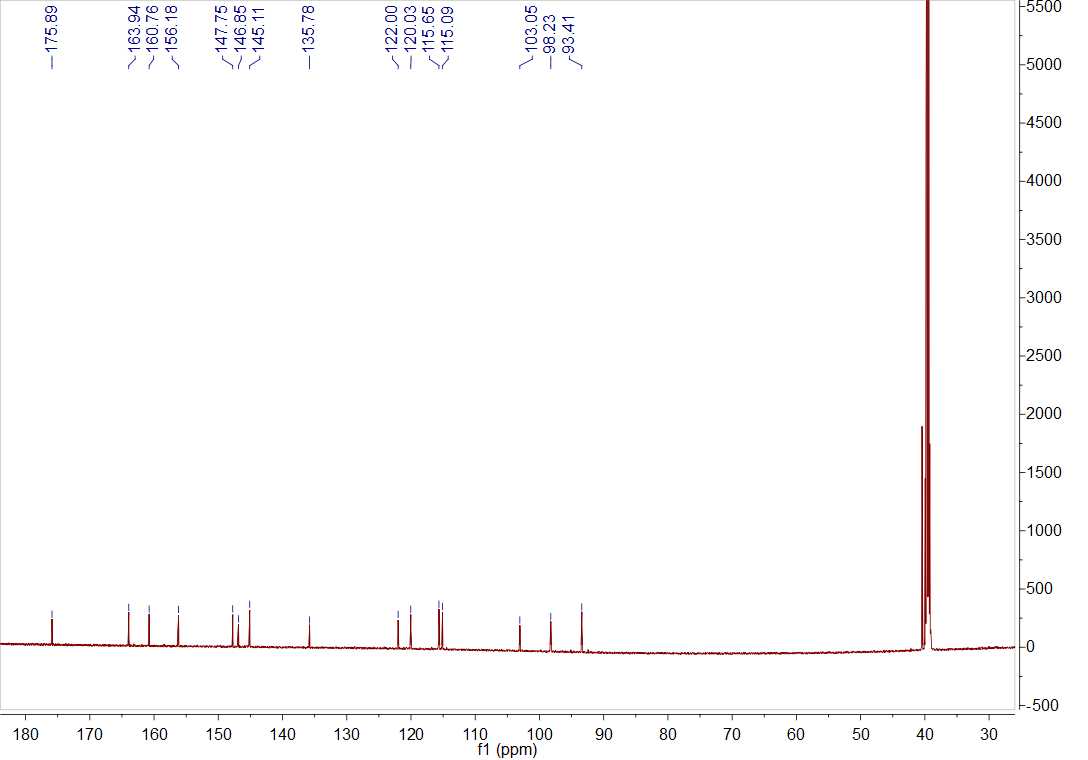


^1^H NMR spectrum (600 MHz) of compound **9** in CD_3_OD.


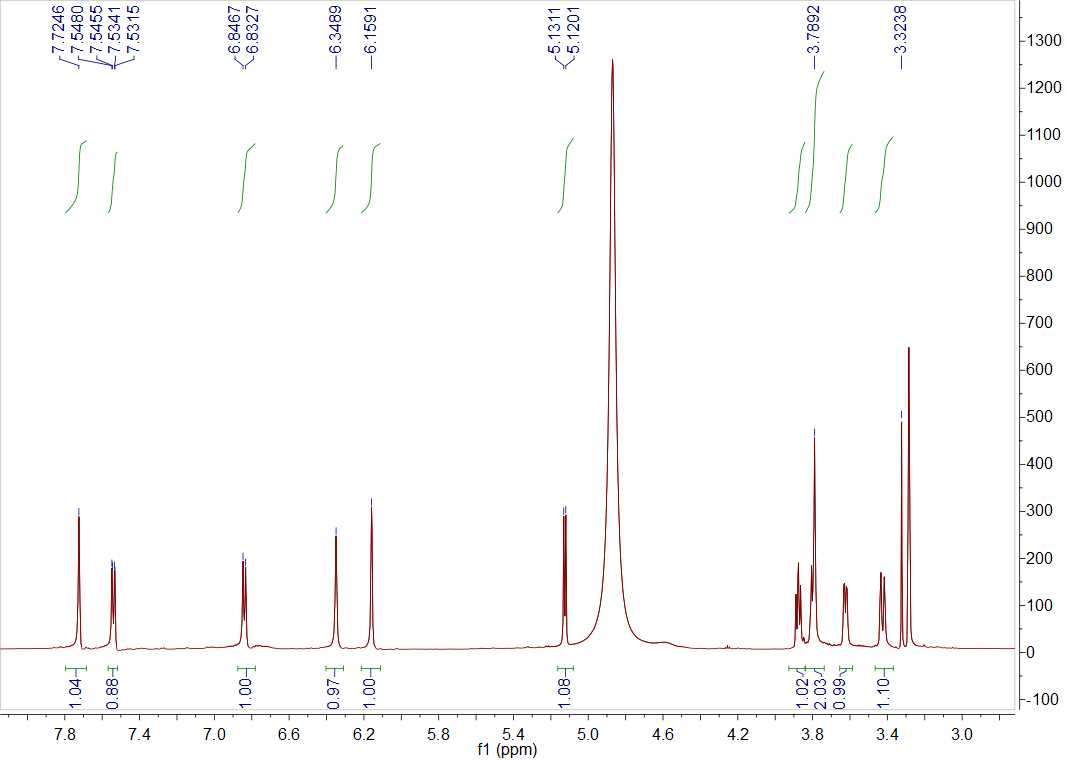


^13^C NMR spectrum (151 MHz) of compound **9** in CD_3_OD.


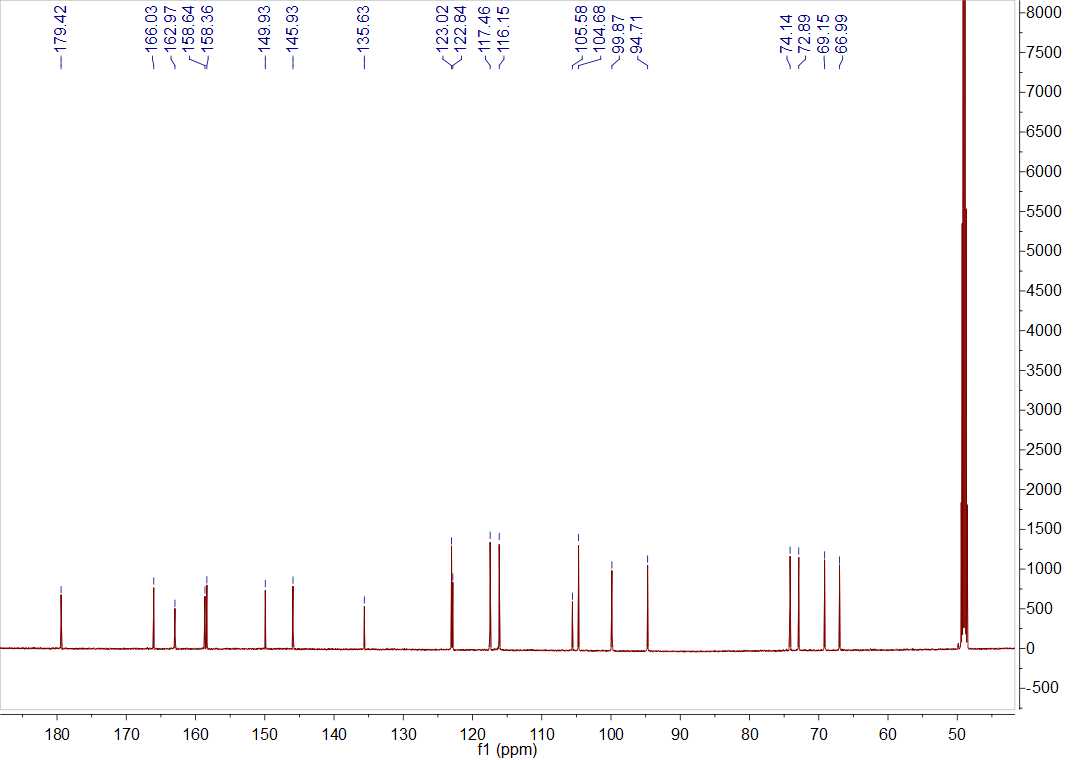


^1^H NMR spectrum (600 MHz) of compound **10** in DMSO-*d*_6_.


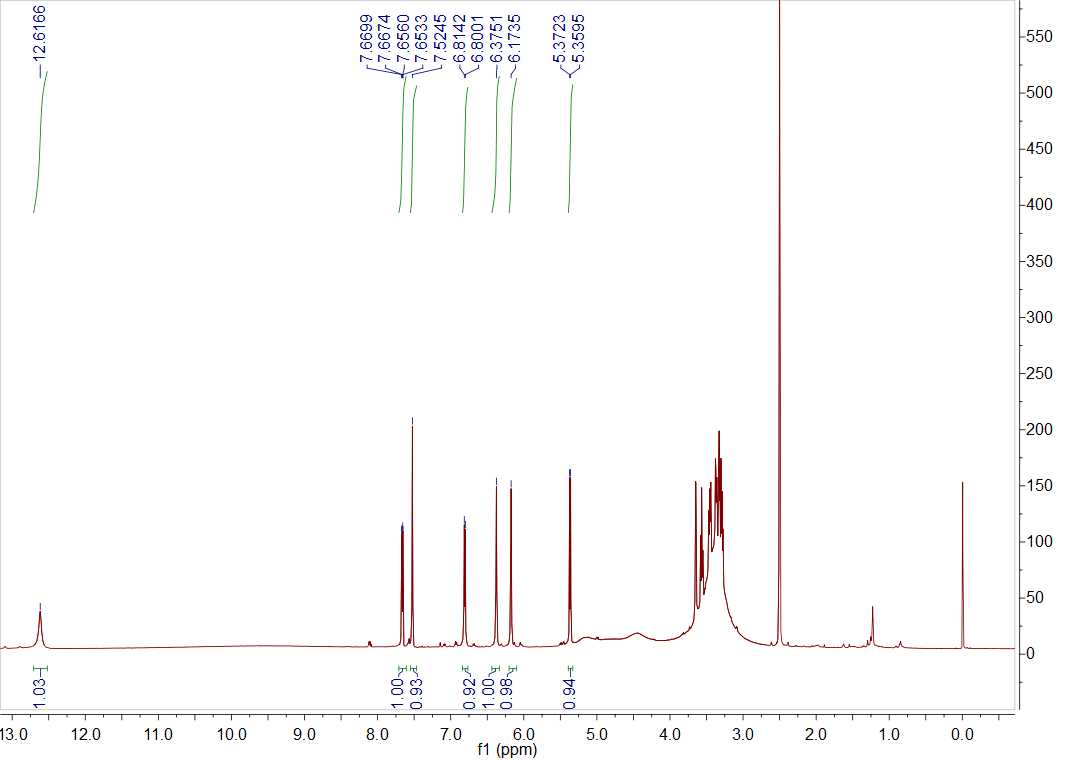


^13^C NMR spectrum (151 MHz) of compound **10** in DMSO-*d*_6_.


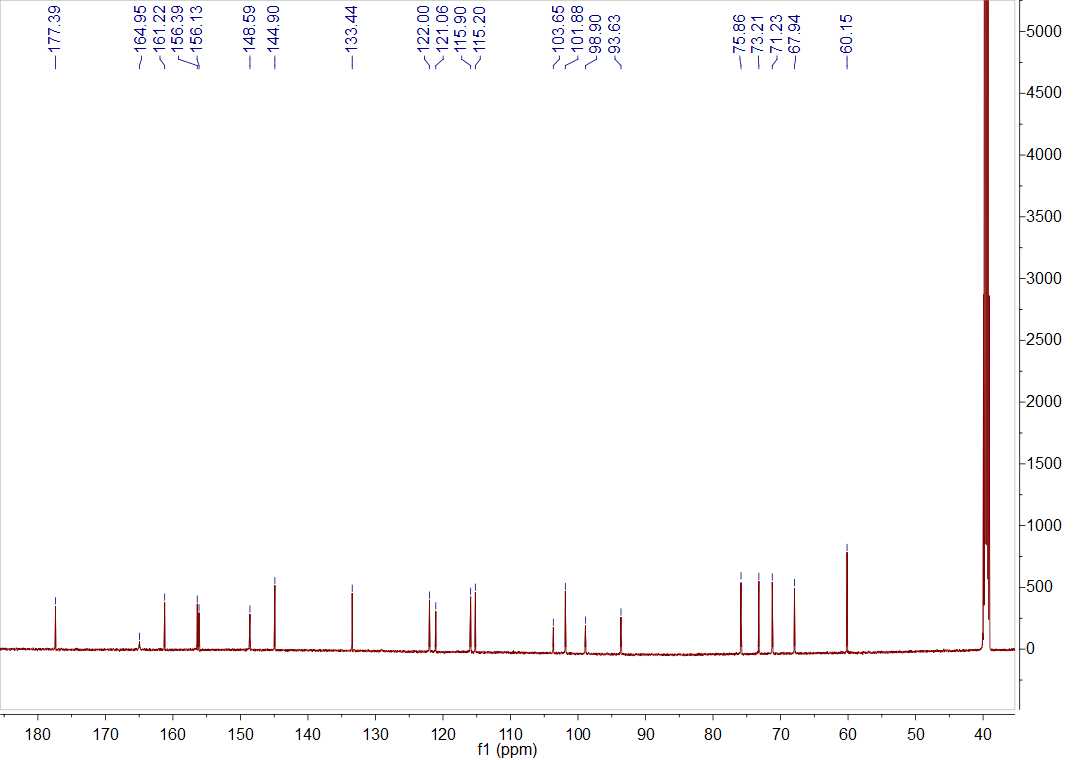


^1^H NMR spectrum (600 MHz) of compound **11** in CD_3_OD.


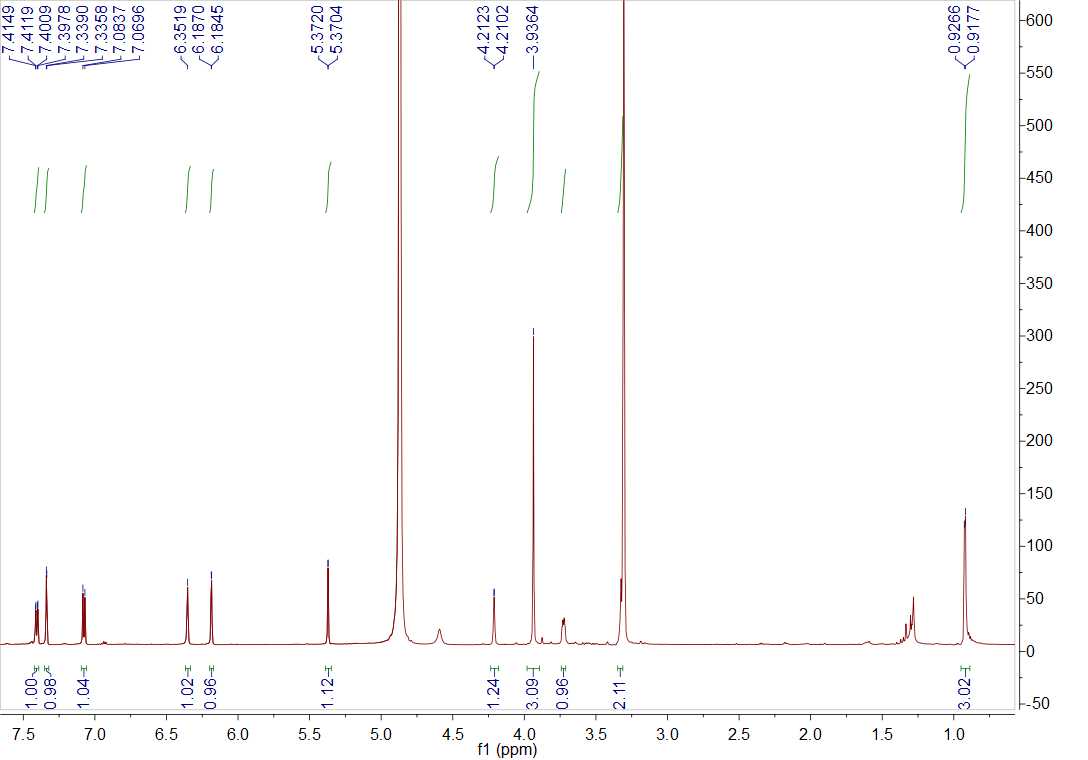


^13^C NMR spectrum (151 MHz) of compound **11** in CD_3_OD.


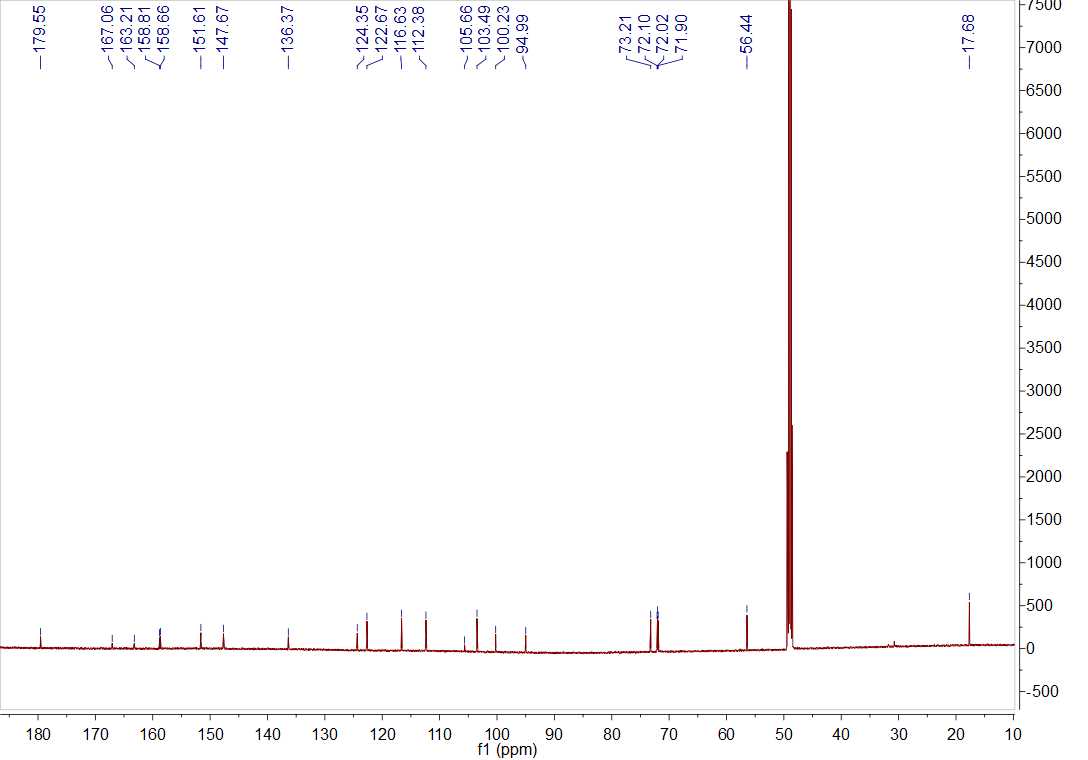


^1^H NMR spectrum (600 MHz) of compound **12** in CD_3_OD.


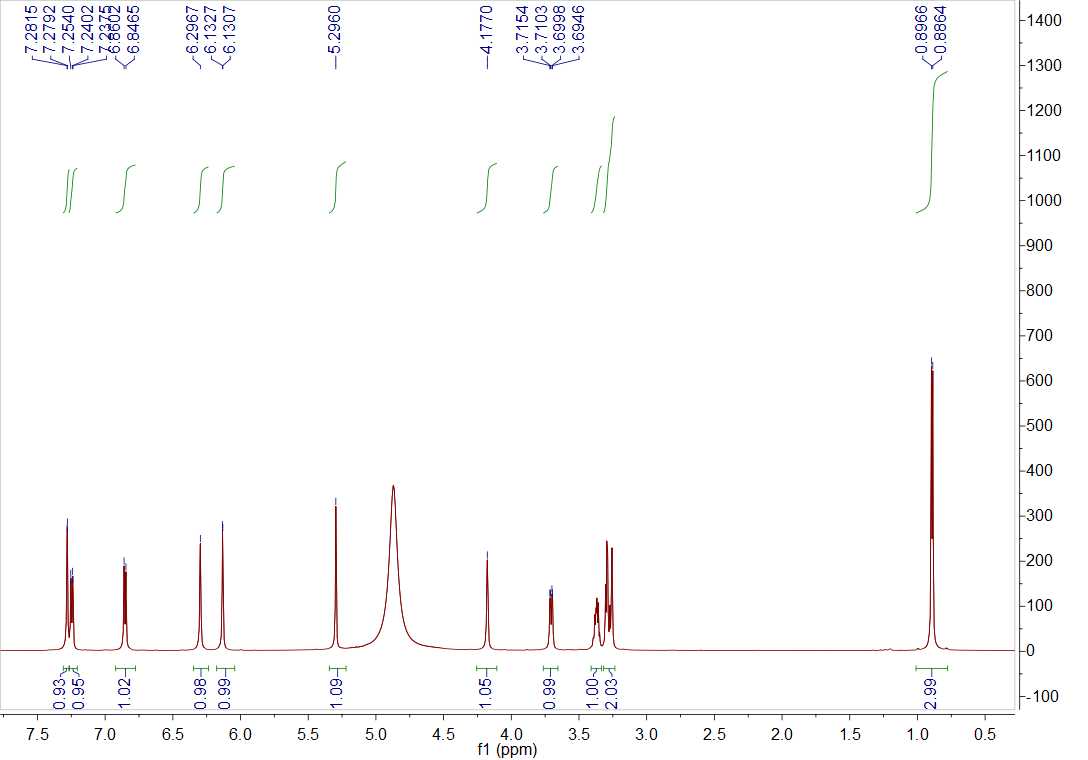


^13^C NMR spectrum (151 MHz) of compound **12** in CD_3_OD.


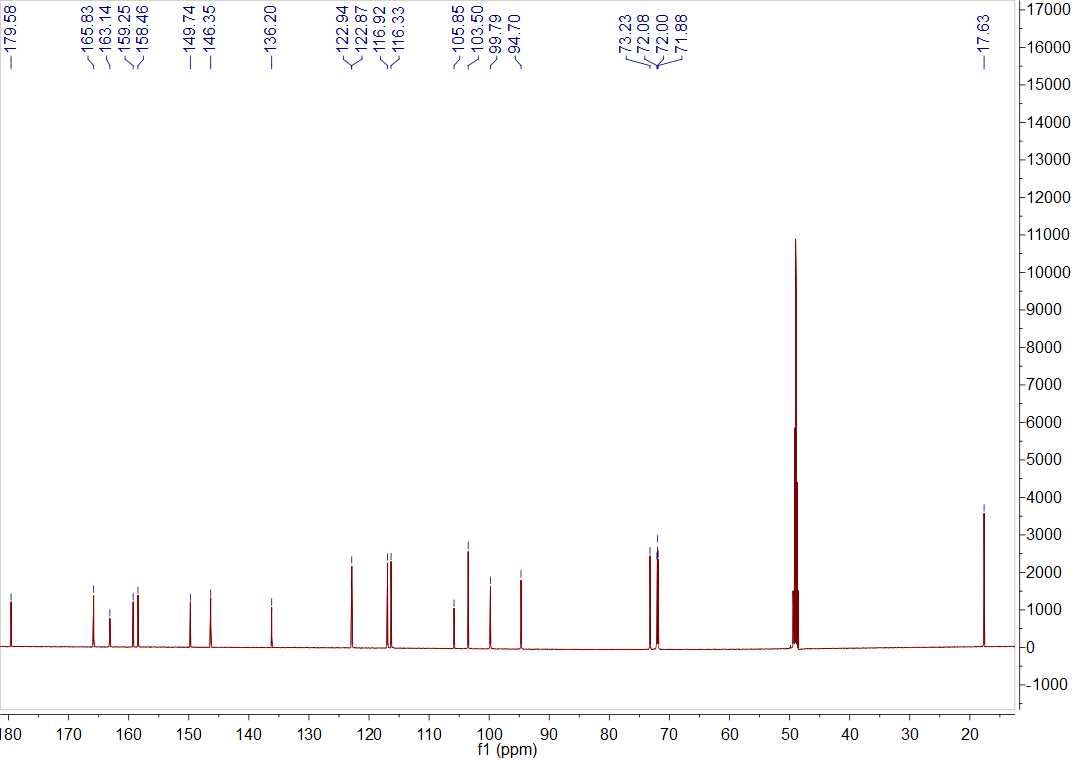


^1^H NMR spectrum (600 MHz) of compound **13** in DMSO-*d*_6_.


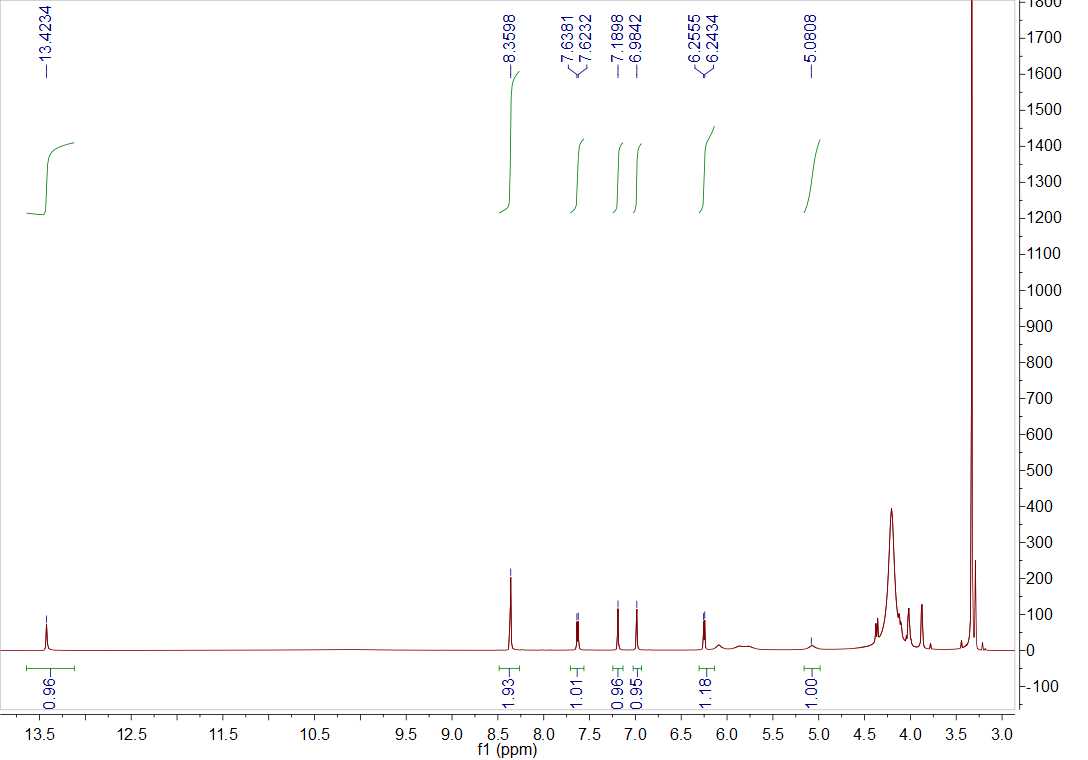


^13^C NMR spectrum (151 MHz) of compound **13** in DMSO-*d*_6_.


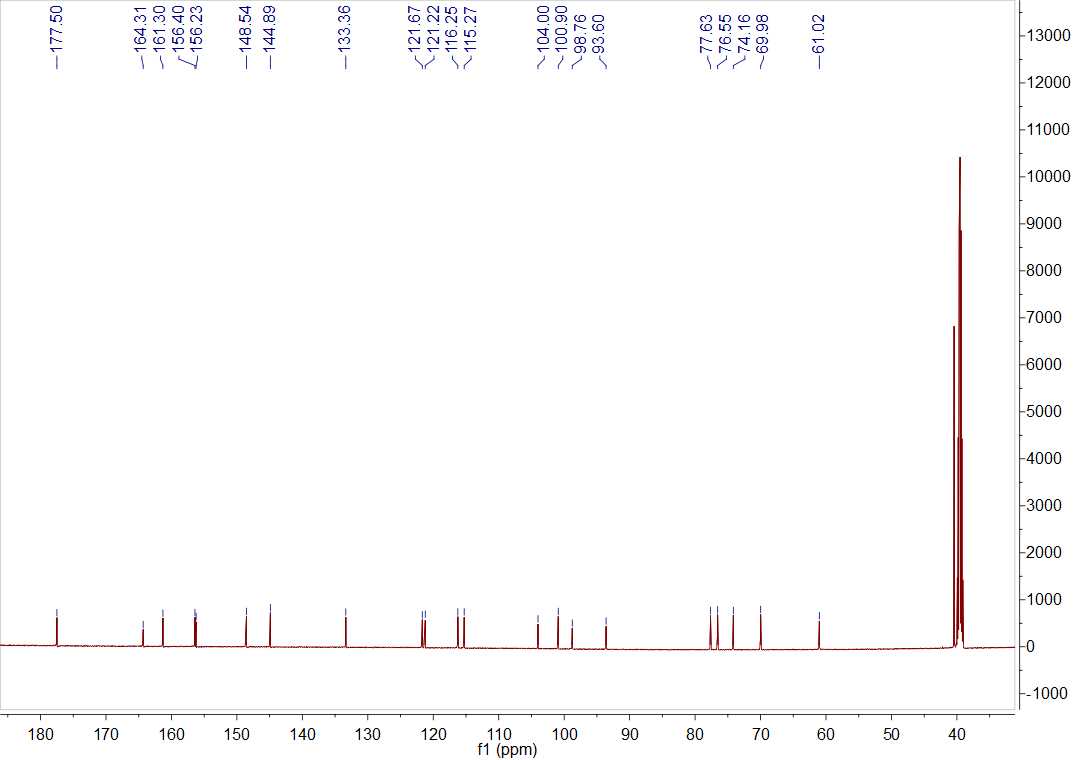


^1^H NMR spectrum (600 MHz) of compound **14** in CD_3_OD.


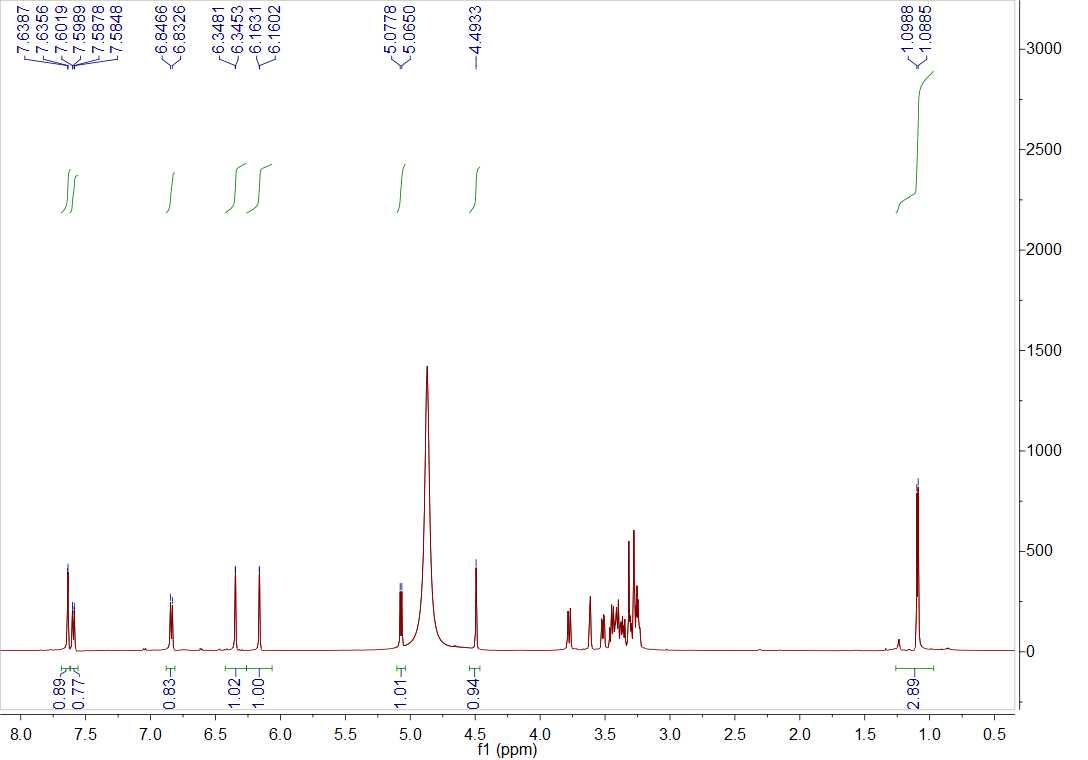


^13^C NMR spectrum (151 MHz) of compound **14** in CD_3_OD.


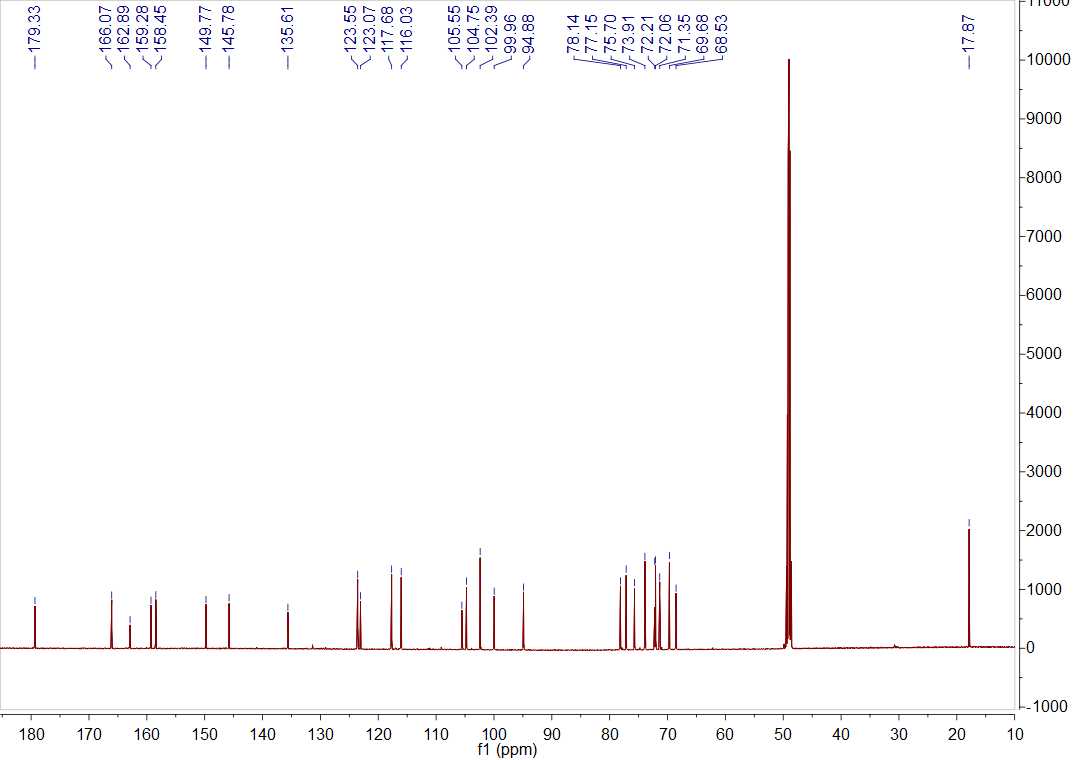


^1^H NMR spectrum (400 MHz) of compound **15** in DMSO-*d*_6_.


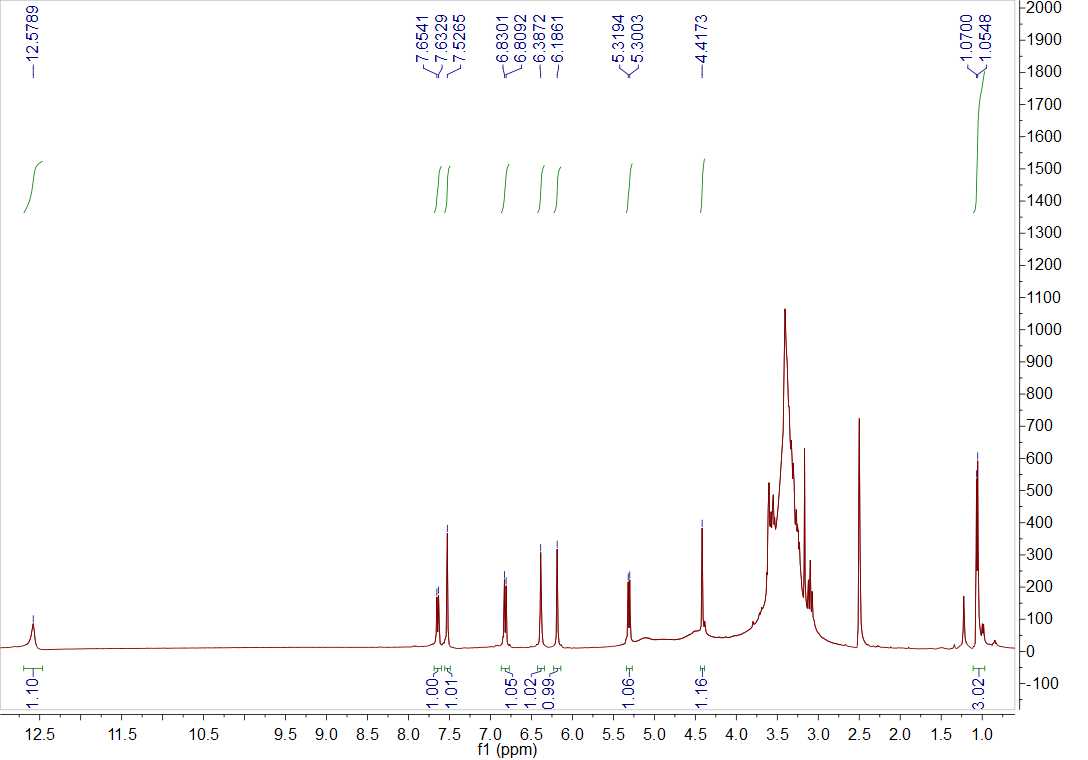


^13^C NMR spectrum (101 MHz) of compound **15** in DMSO-*d*_6_.


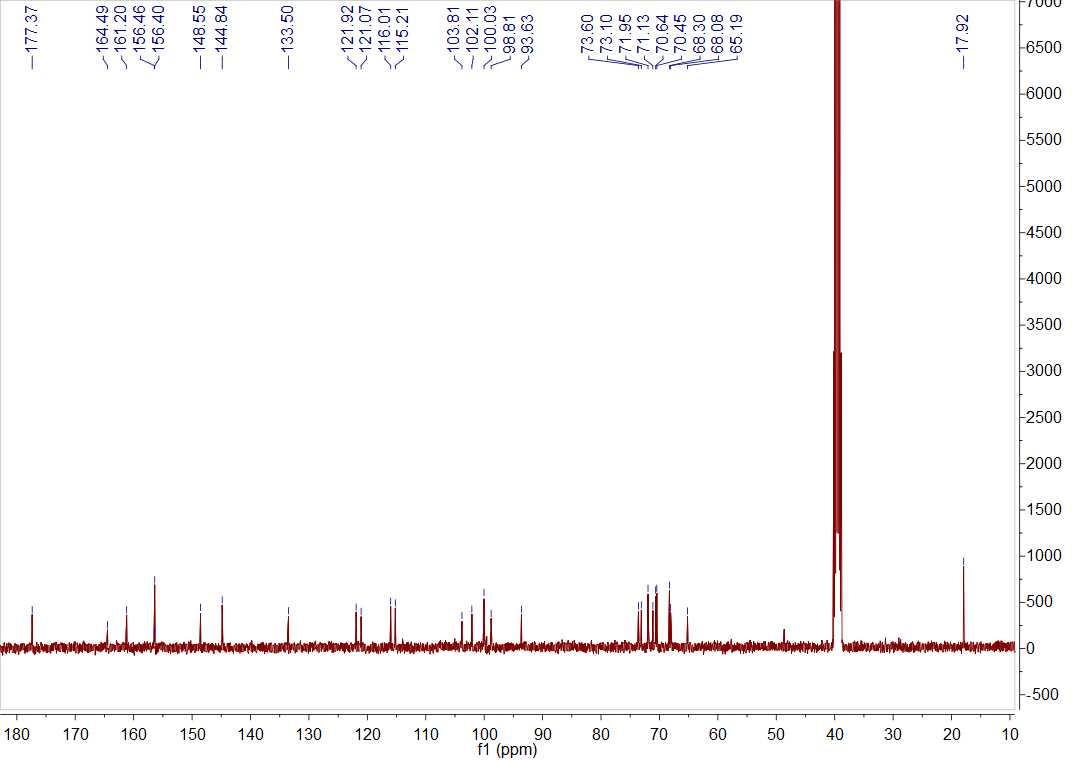


^1^H NMR spectrum (600 MHz) of compound **16** in DMSO-*d*_6_.


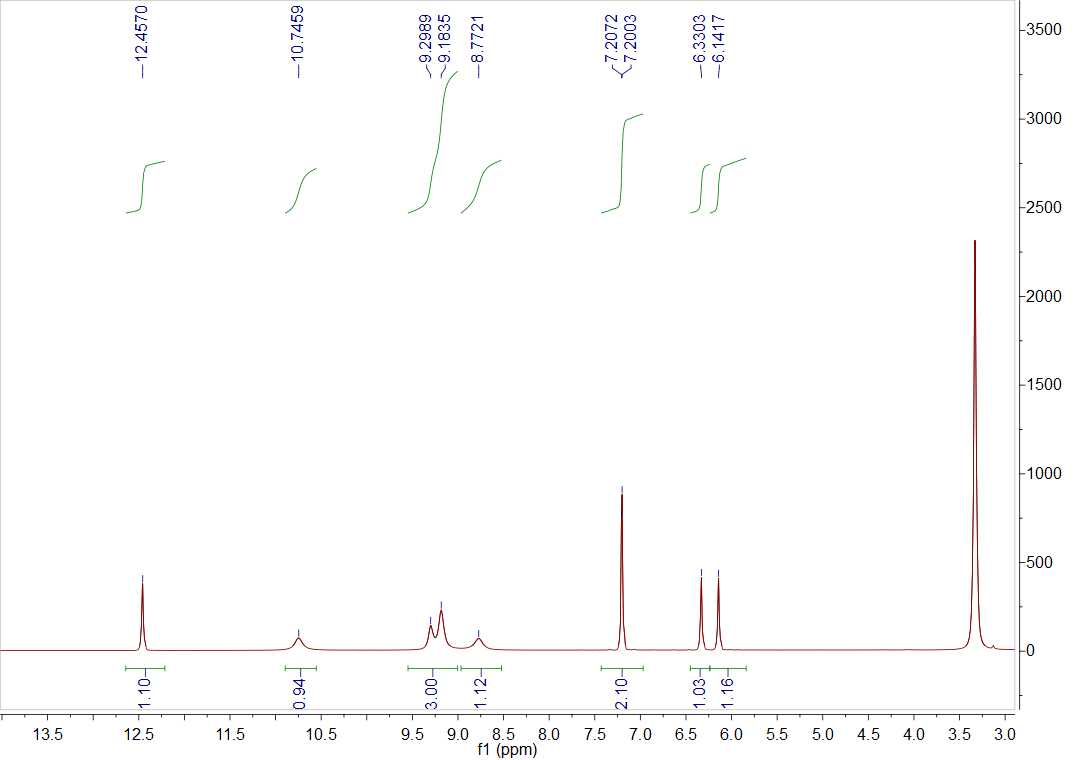


^13^C NMR spectrum (151 MHz) of compound **16** in DMSO-*d*_6_.


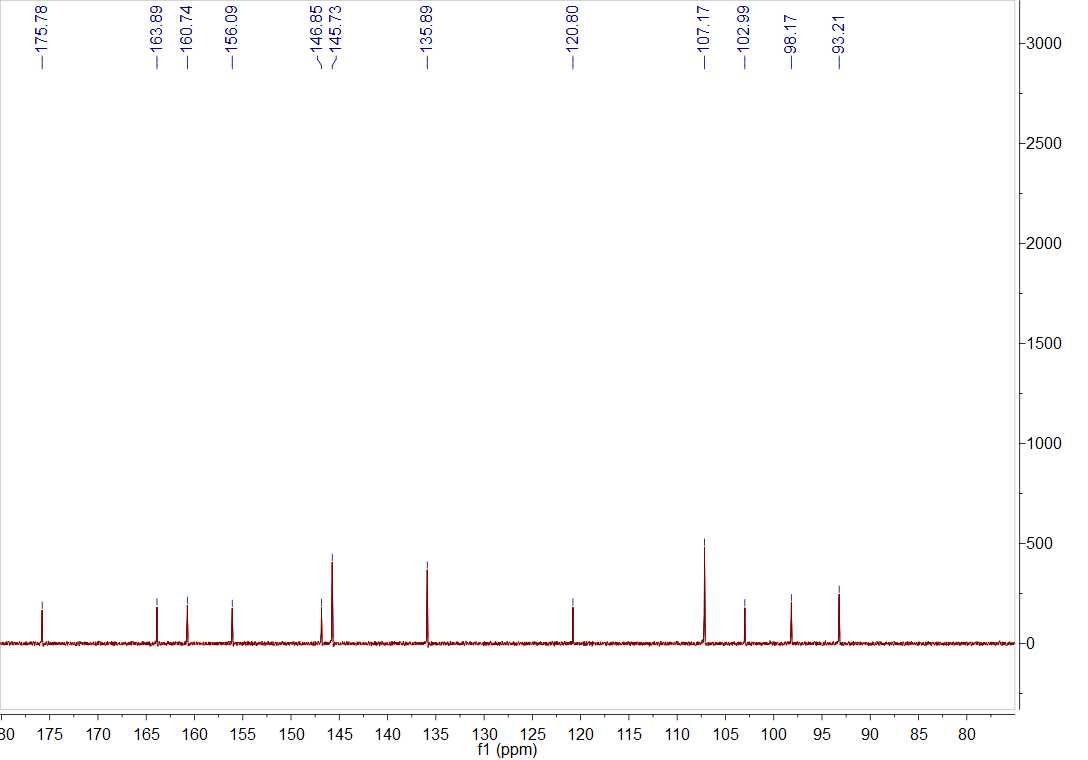


^1^H NMR spectrum (600 MHz) of compound **17** in DMSO-*d*_6_.


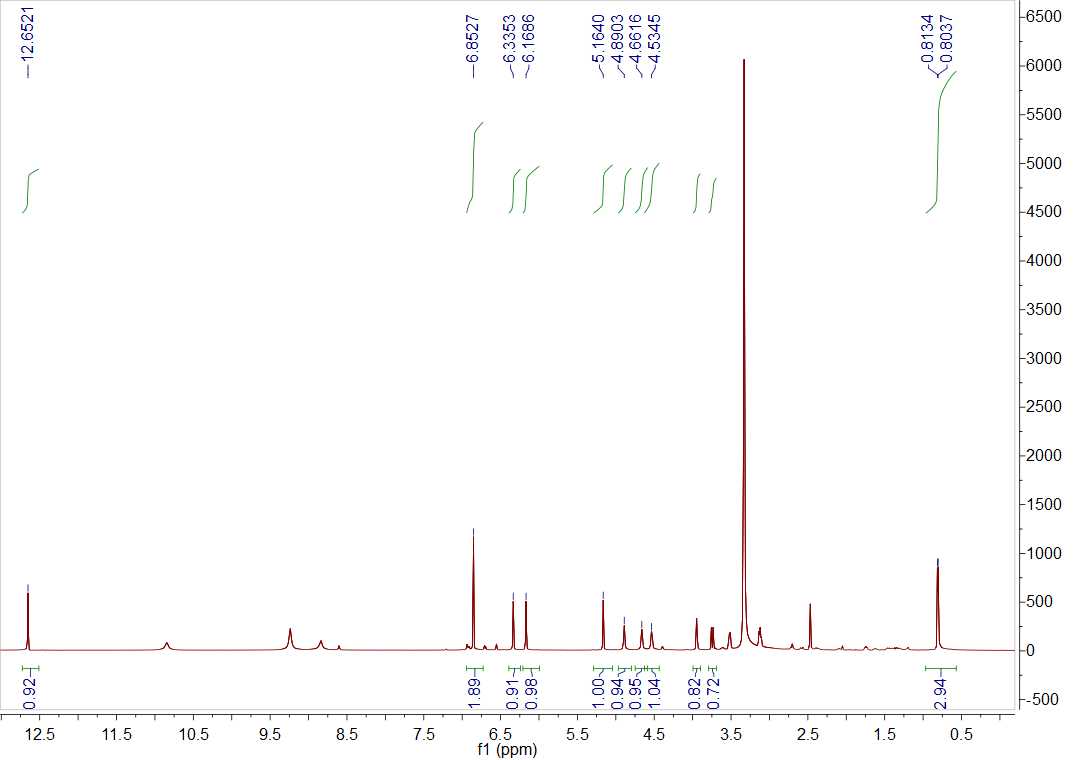


^13^C NMR spectrum (151 MHz) of compound **17** in DMSO-*d*_6_.


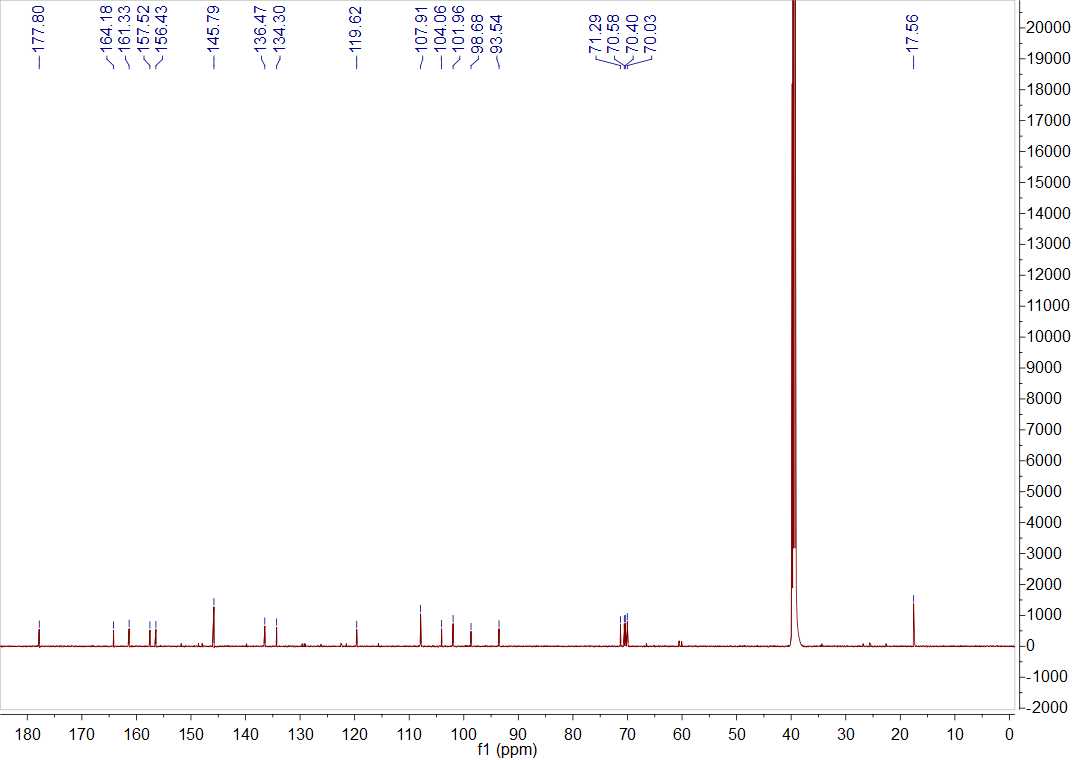


^1^H NMR spectrum (400 MHz) of compound **18** in CD_3_OD.


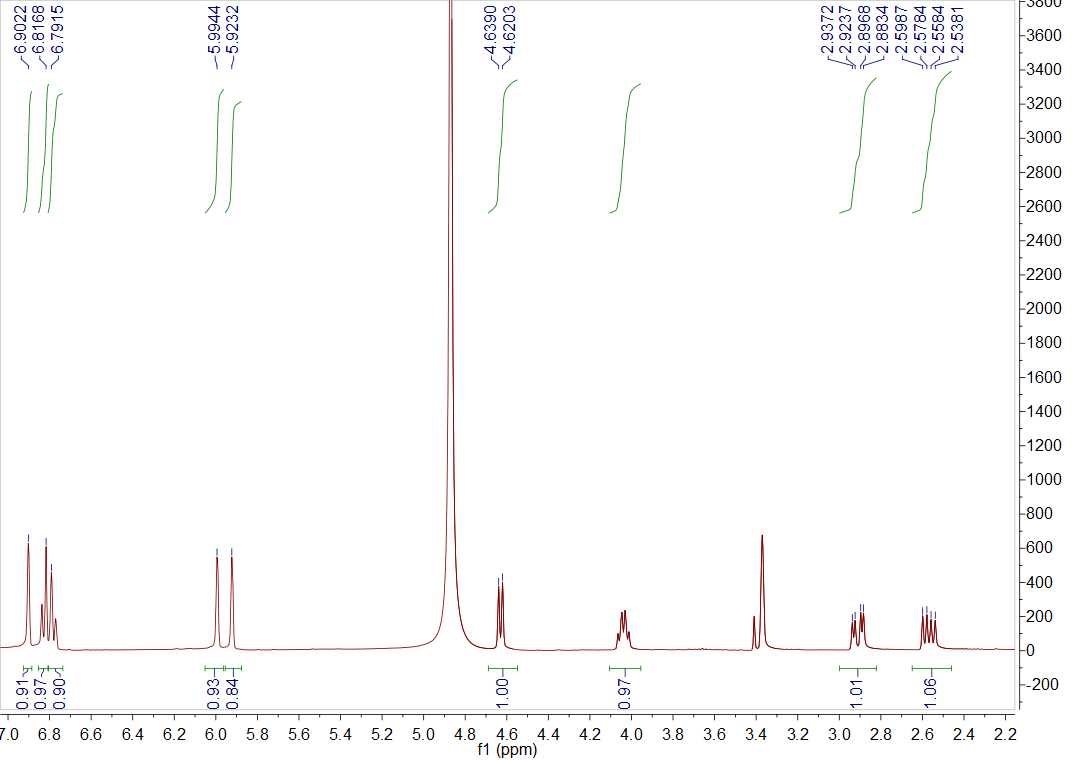


^13^C NMR spectrum (101 MHz) of compound **18** in CD_3_OD.


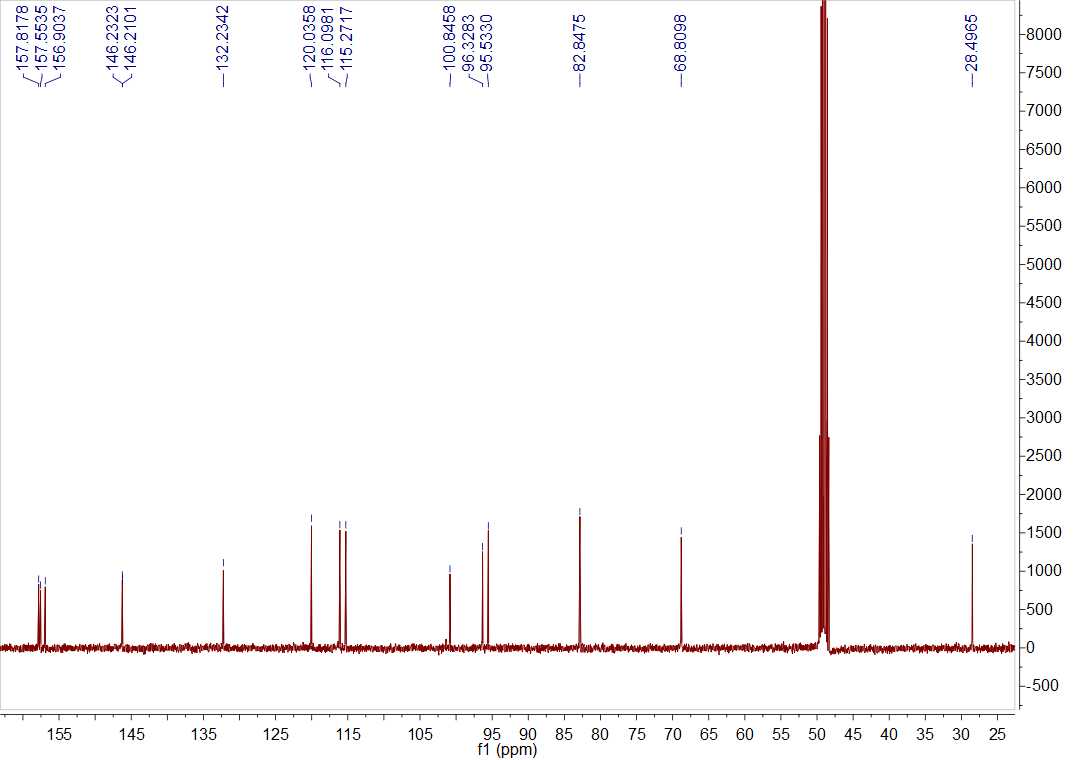


^1^H NMR spectrum (600 MHz) of compound **19** in CD_3_OD.


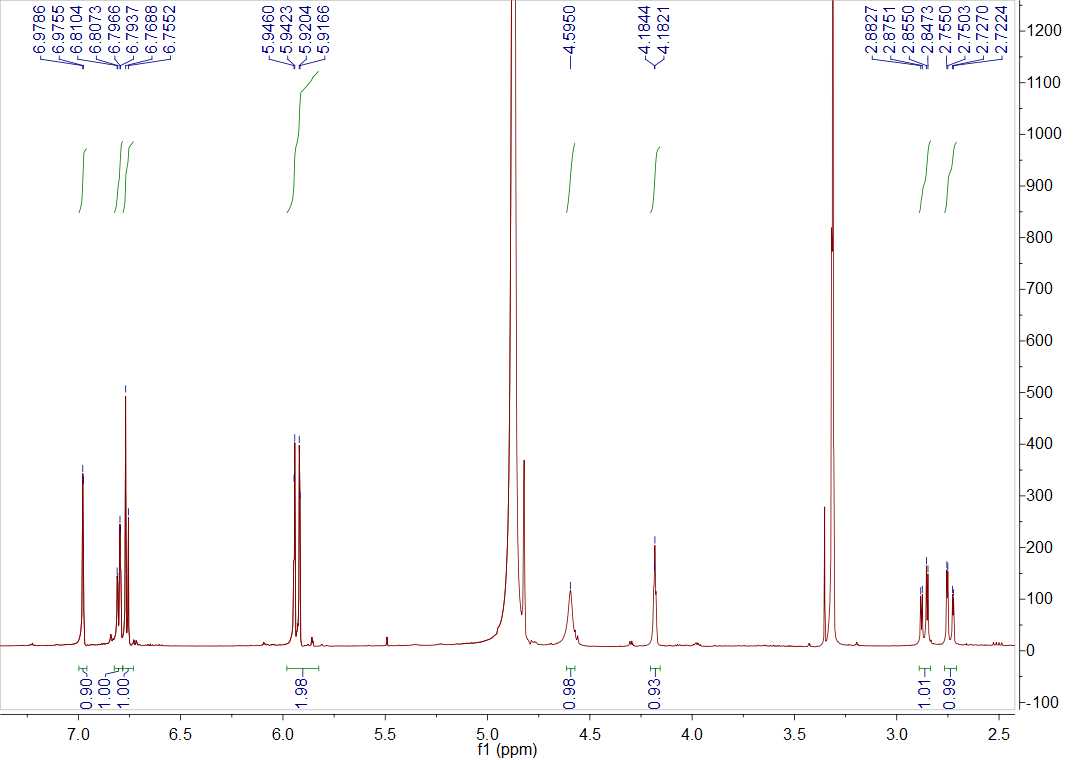


^13^C NMR spectrum (151 MHz) of compound **19** in CD_3_OD.


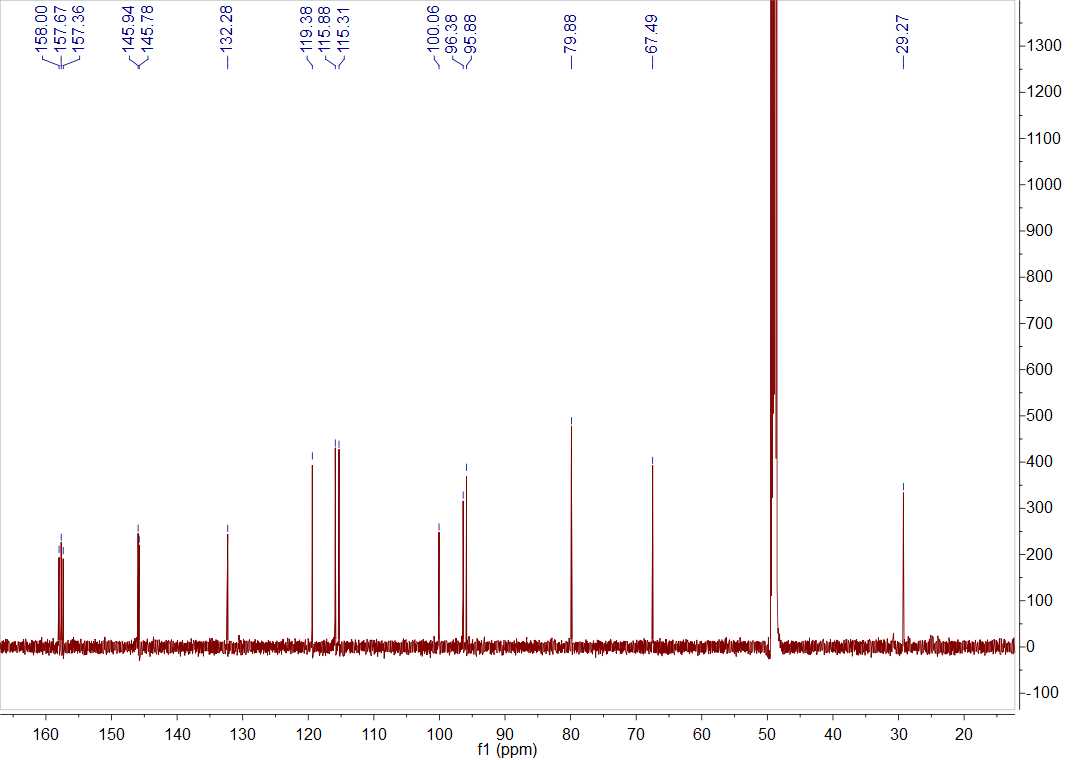


^1^H NMR spectrum (600 MHz) of compound **20** in CD_3_OD.


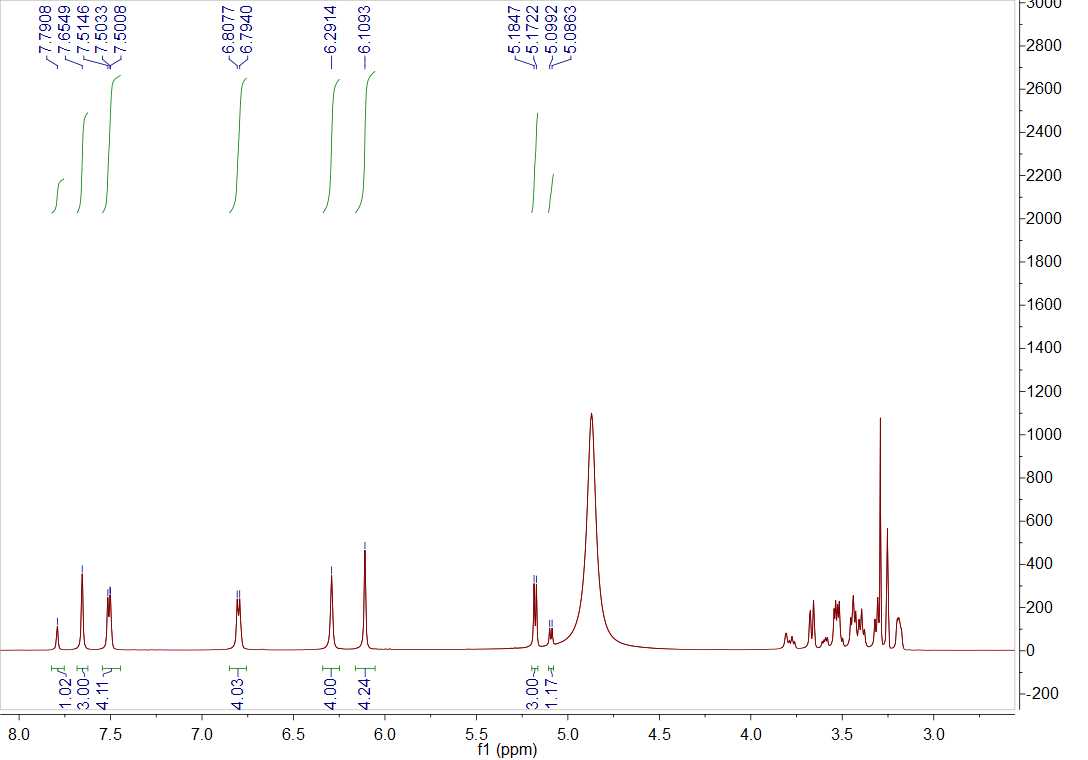


^13^C NMR spectrum (151 MHz) of compound **20** in CD_3_OD.


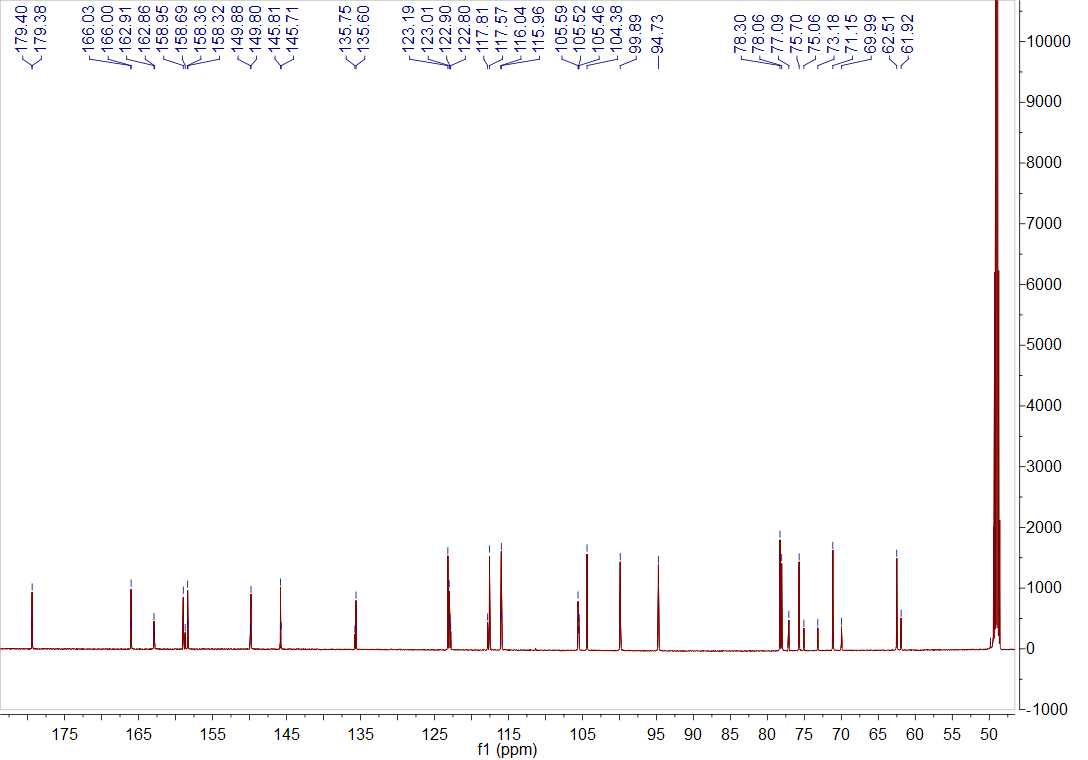


^1^H NMR spectrum (400 MHz) of compound **21** in DMSO-*d*_6_.


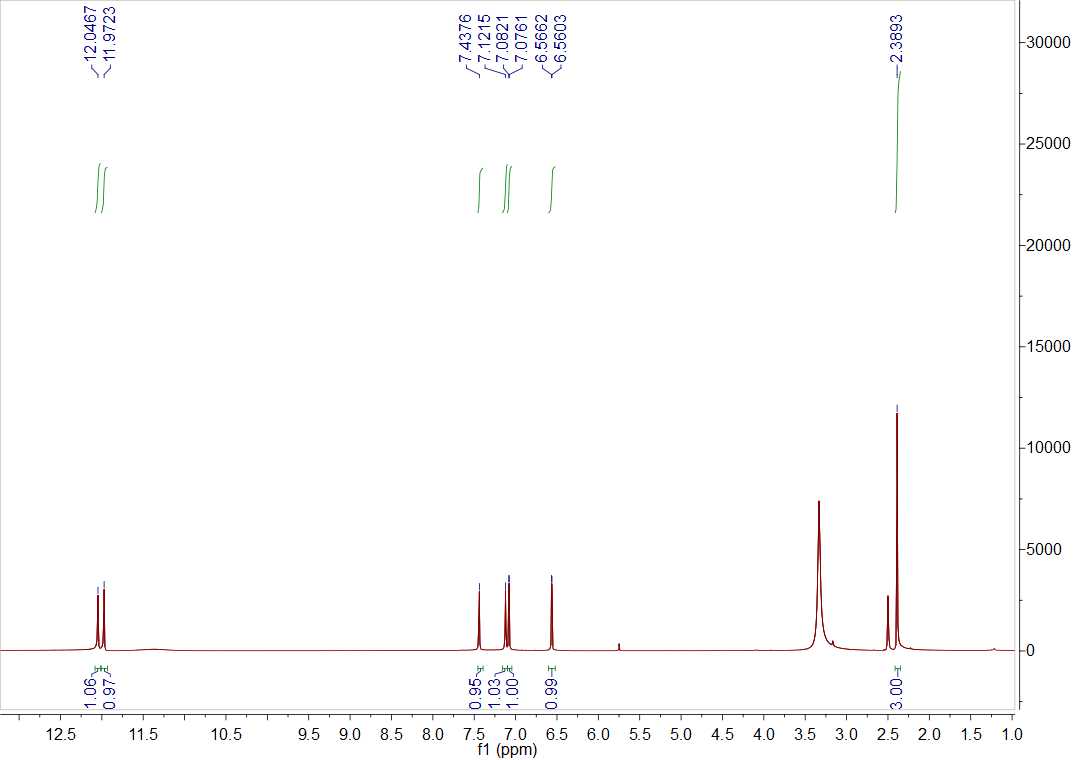


^13^C NMR spectrum (101 MHz) of compound **21** in DMSO-*d*_6_.


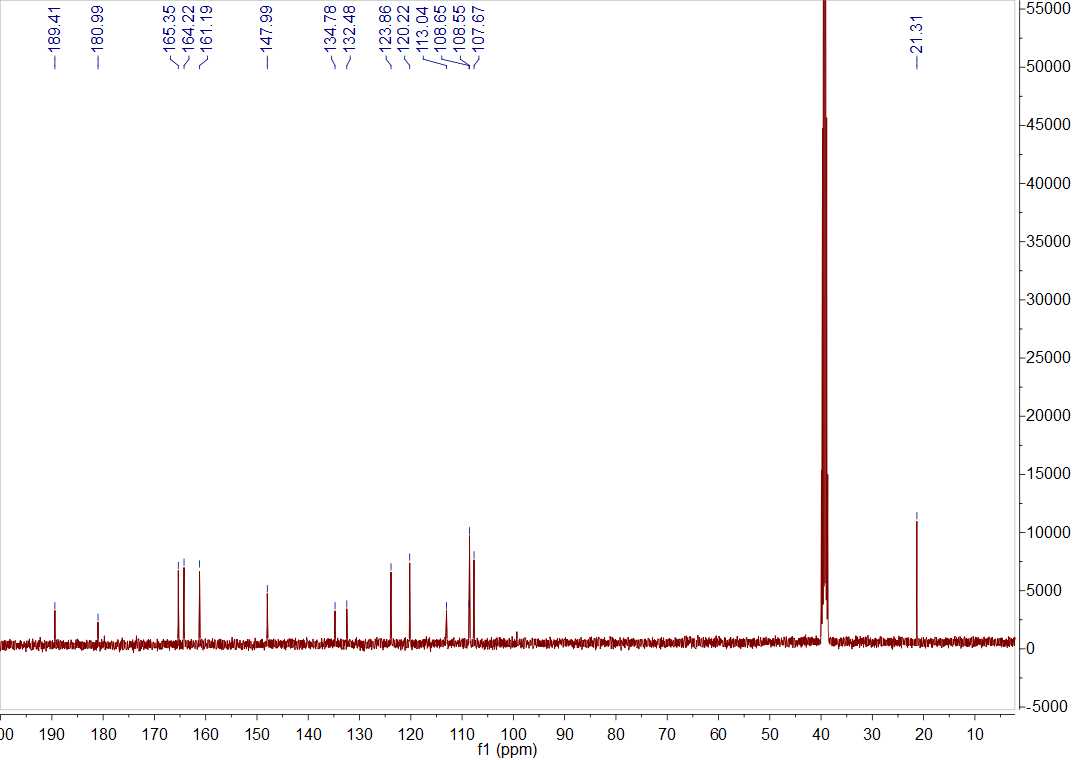


^1^H NMR spectrum (400 MHz) of compound **22** in DMSO-*d*_6_.


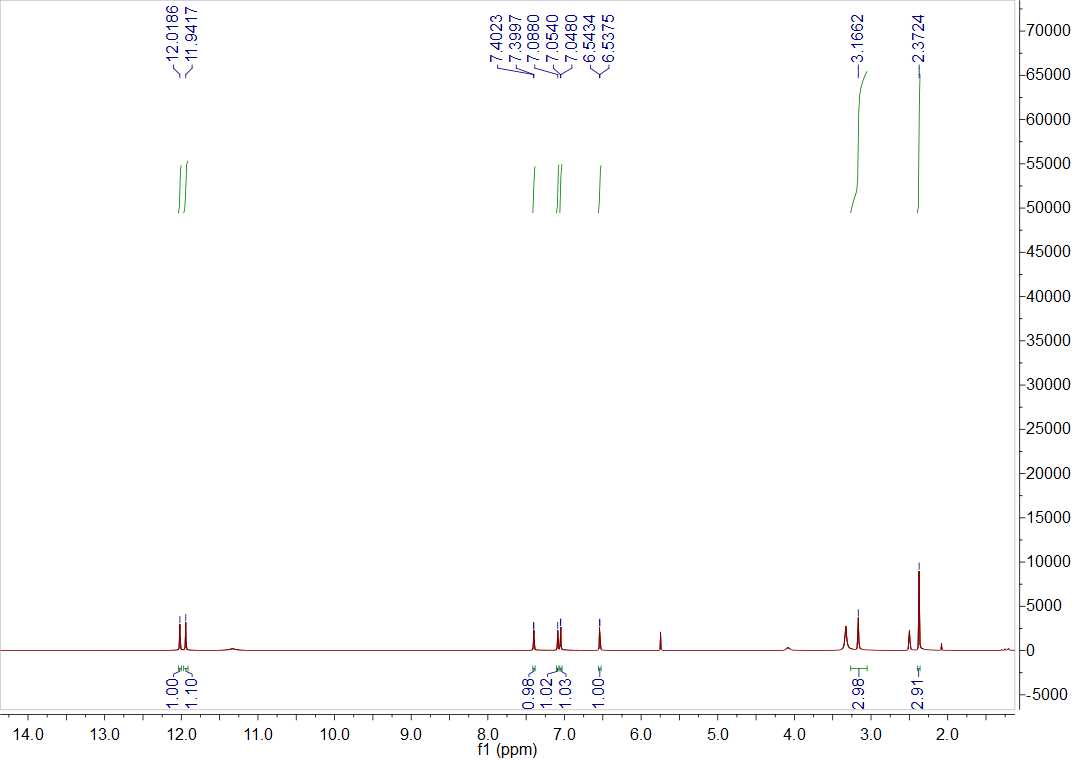


^13^C NMR spectrum (101 MHz) of compound **22** in DMSO-*d*_6_.


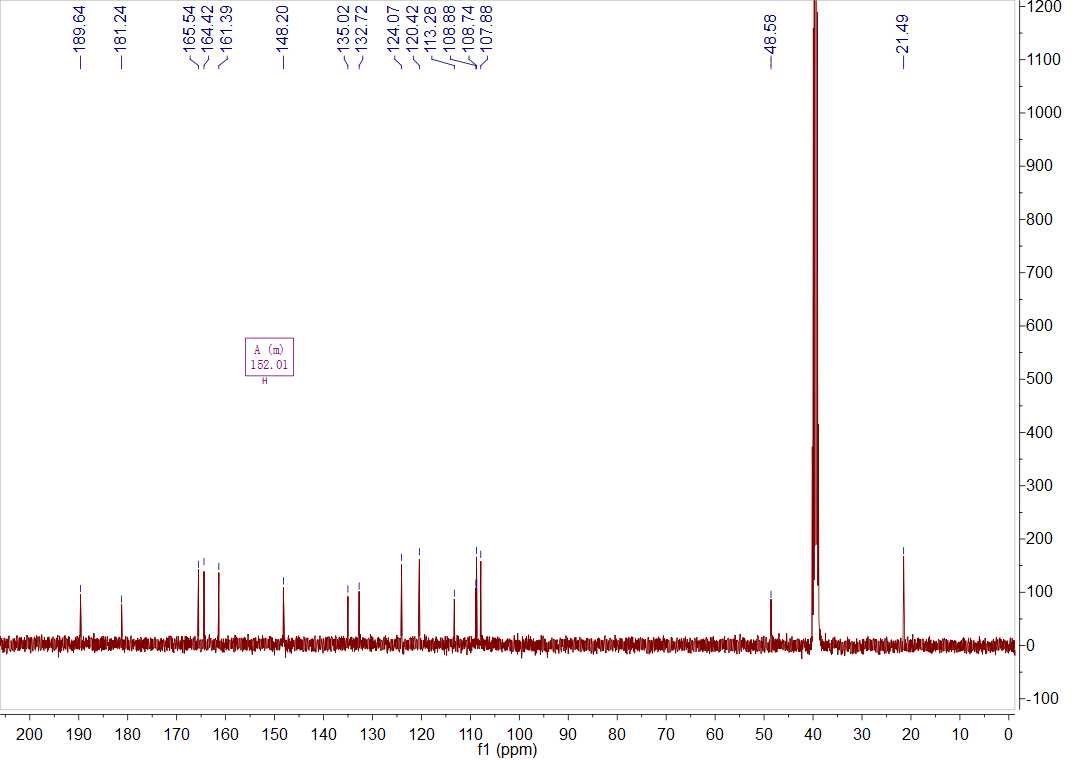


^1^H NMR spectrum (600 MHz) of compound **23** in CD_3_OD.


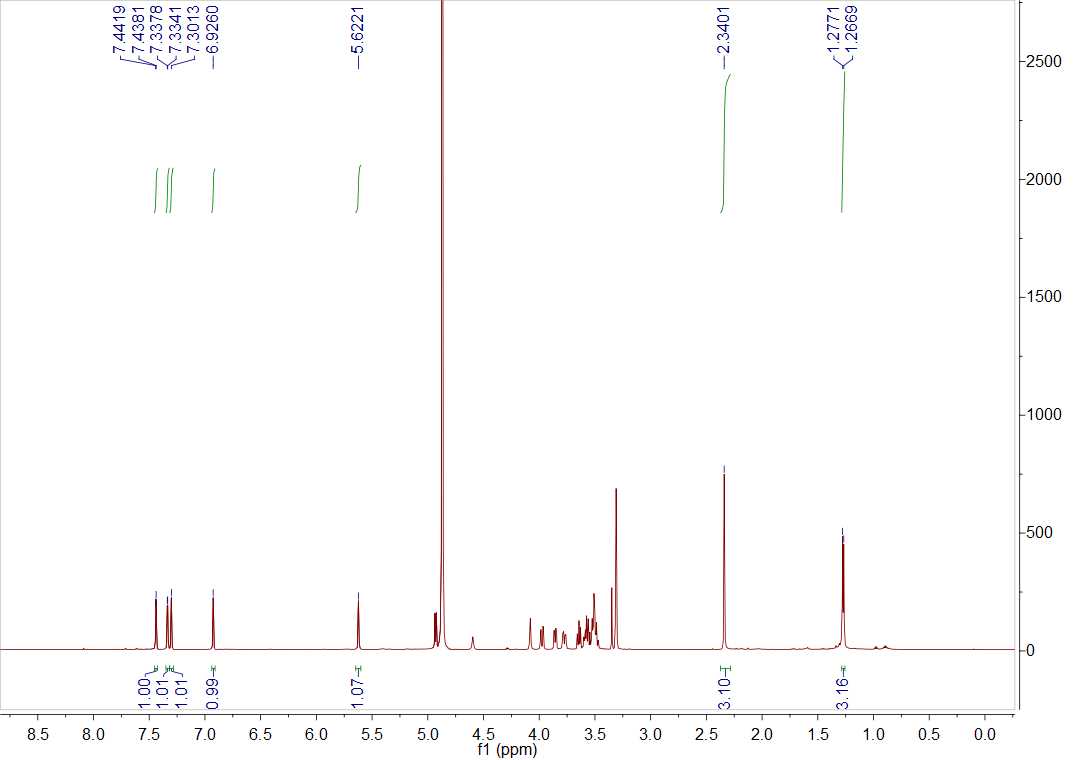


^13^C NMR spectrum (151 MHz) of compound **23** in CD_3_OD.


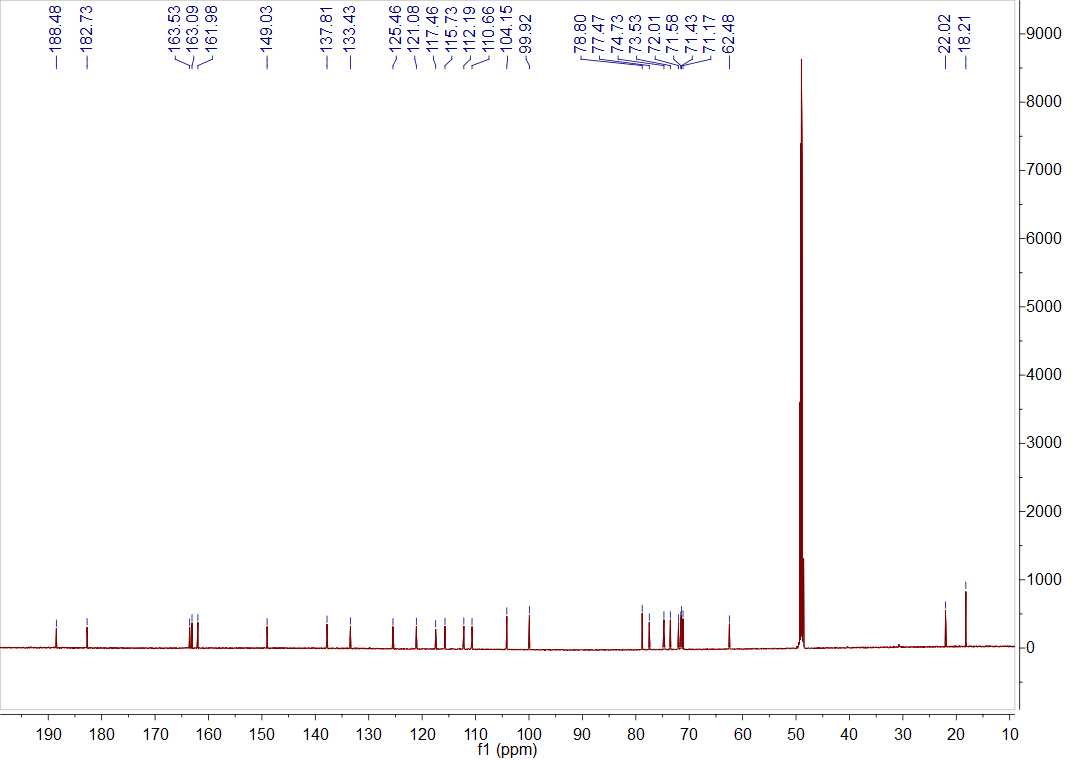


^1^H NMR spectrum (600 MHz) of compound **24** in CD_3_OD.


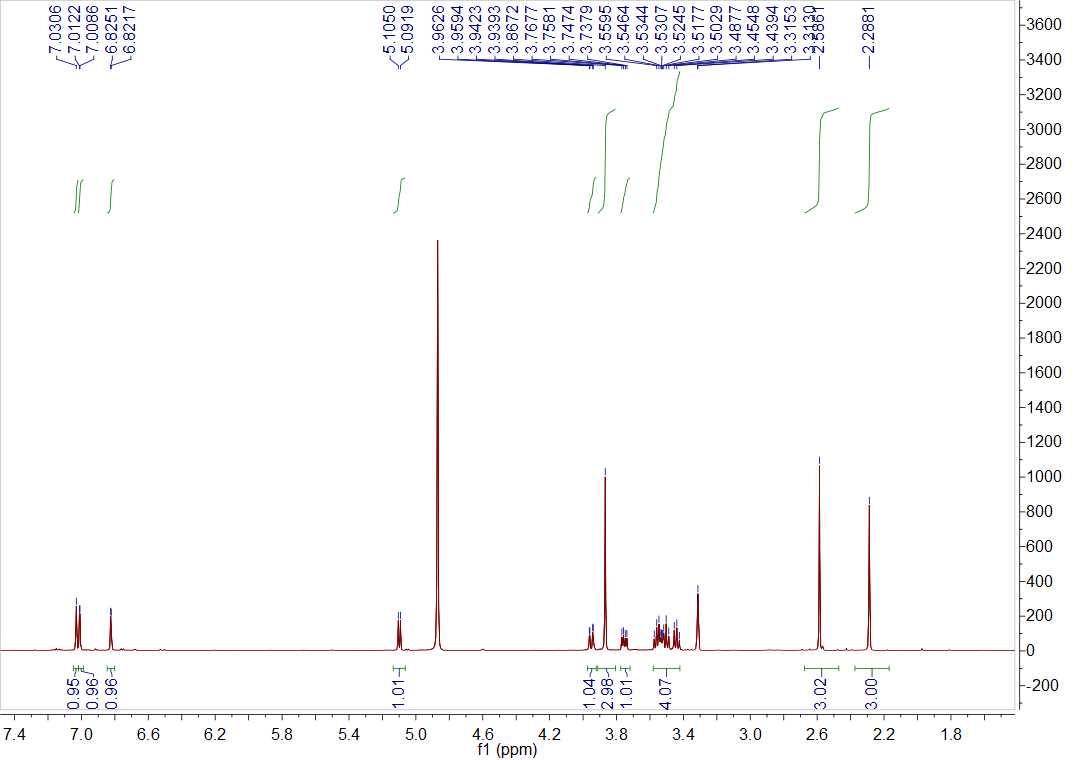


^13^C NMR spectrum (151 MHz) of compound **24** in CD_3_OD.


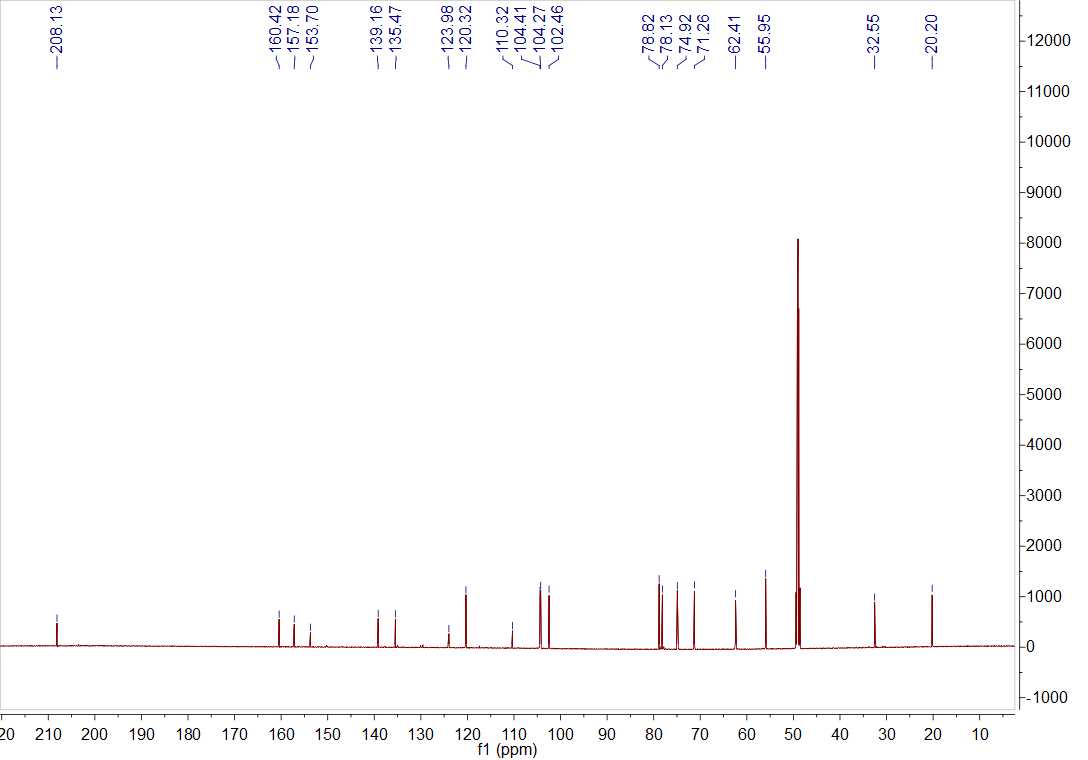


^1^H NMR spectrum (600 MHz) of compound **25** in CD_3_OD.


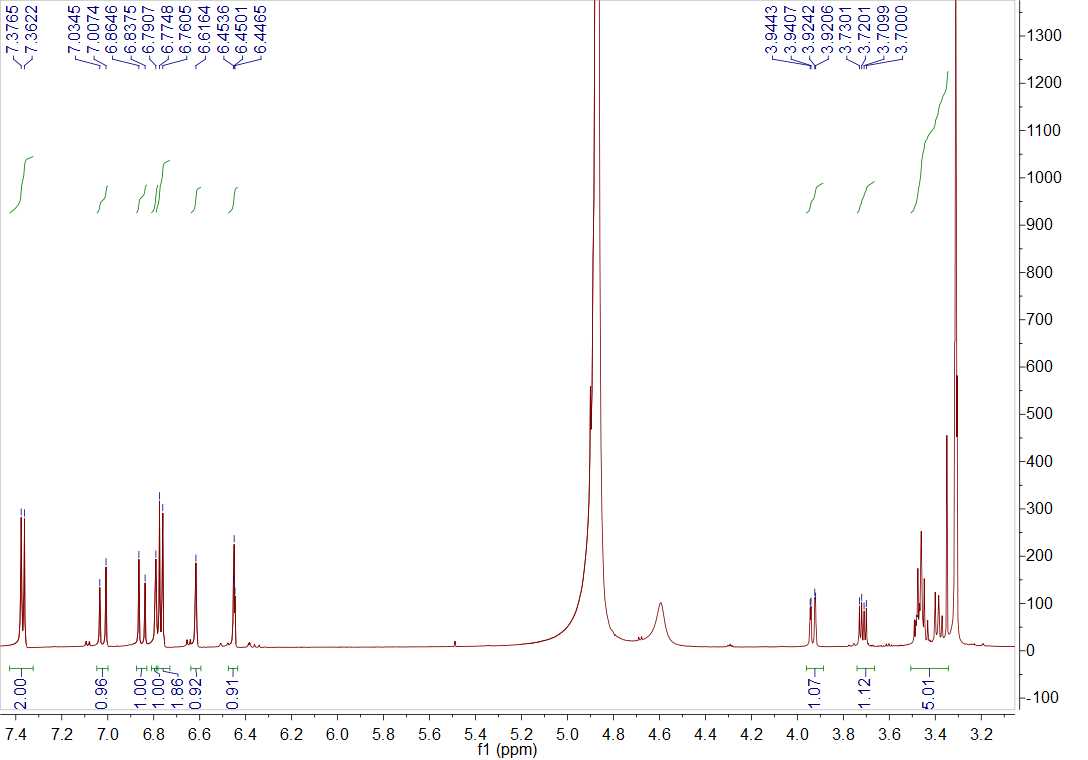


^13^C NMR spectrum (151 MHz) of compound **25** in CD_3_OD.


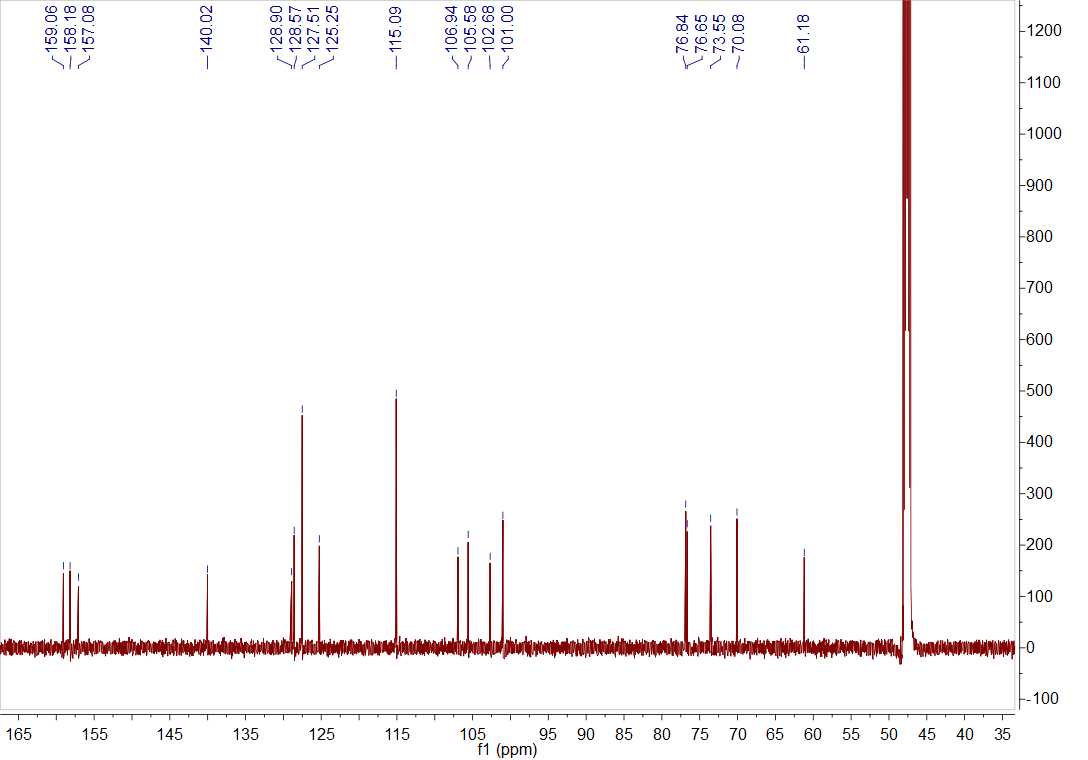


^1^H NMR spectrum (600 MHz) of compound **26** in CD_3_OD.


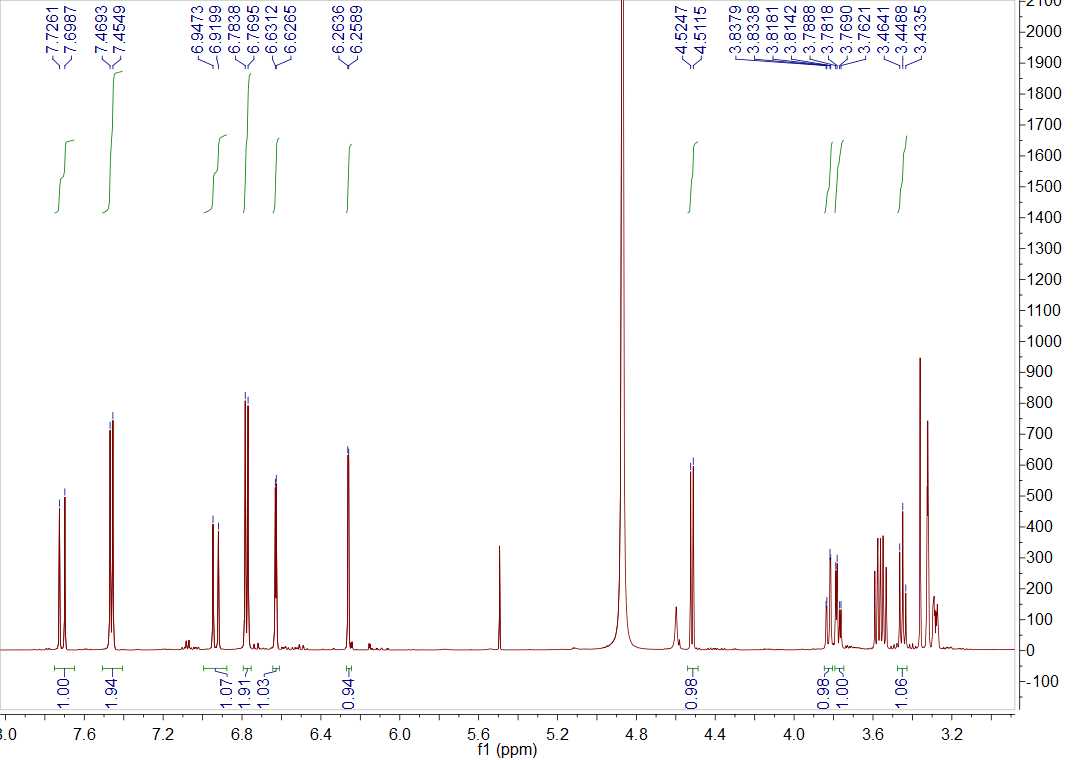


^13^C NMR spectrum (151 MHz) of compound **26** in CD_3_OD.


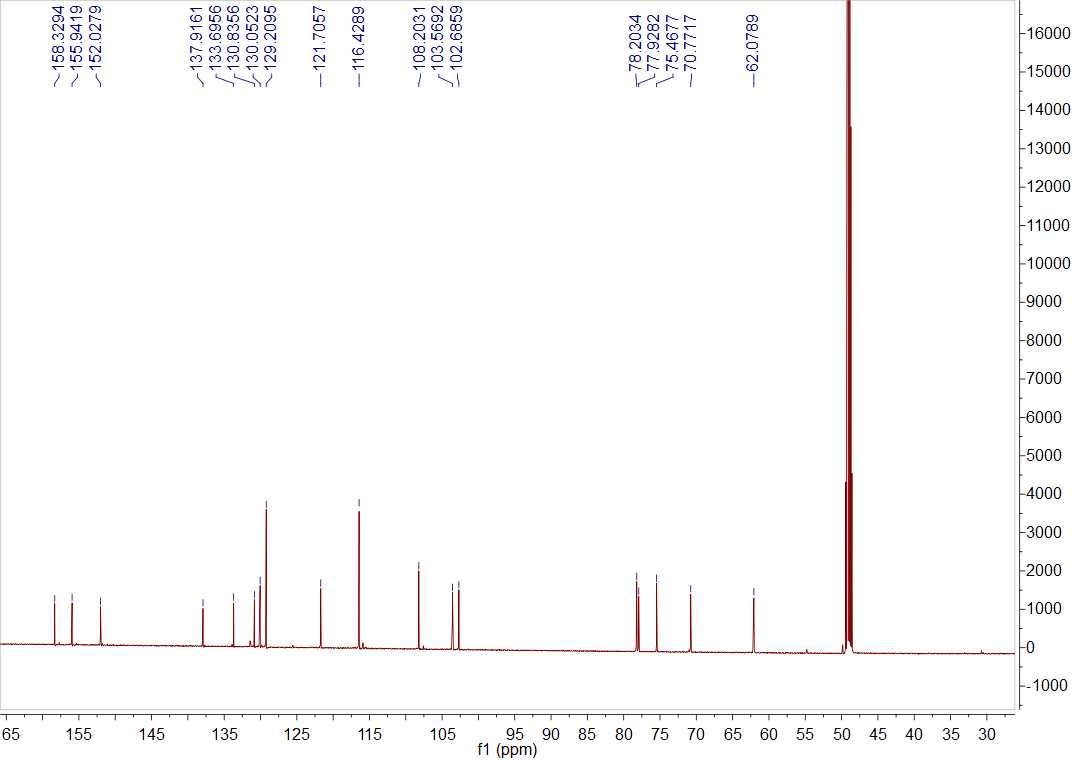


^1^H NMR spectrum (400 MHz) of compound **27** in CD_3_OD.


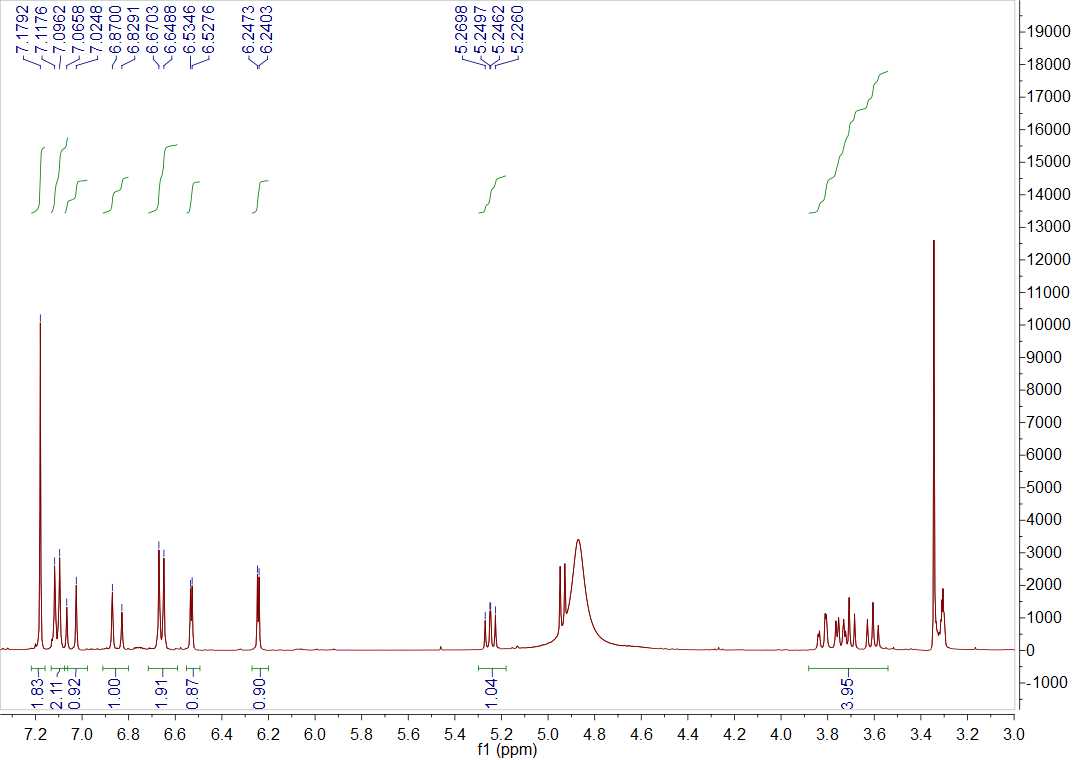


^13^C NMR spectrum (101 MHz) of compound **27** in CD_3_OD.


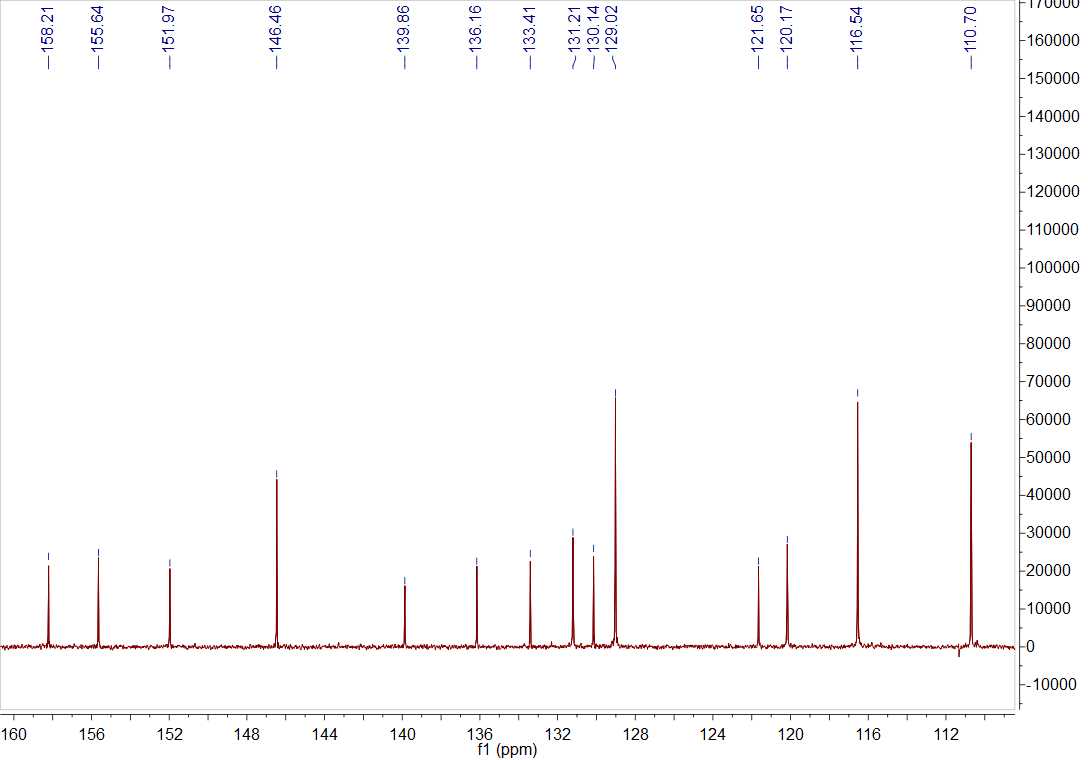


^1^H NMR spectrum (400 MHz) of compound **28** in CD_3_OD.


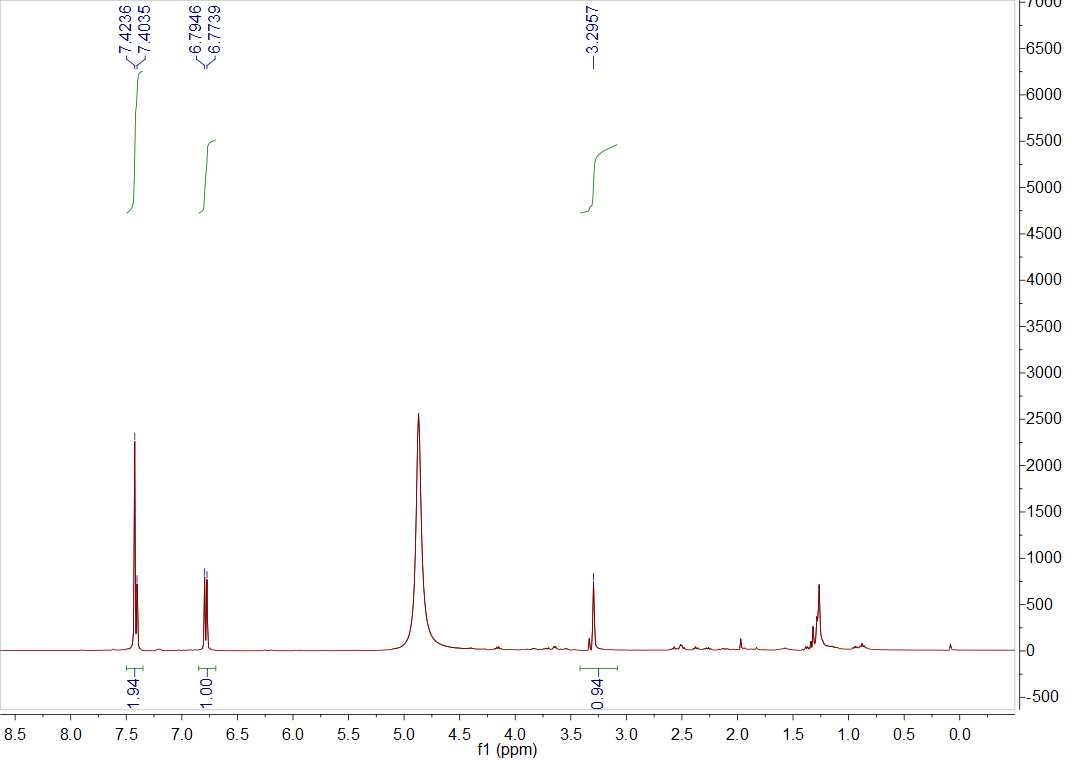


^13^C NMR spectrum (101 MHz) of compound **28** in CD_3_OD.


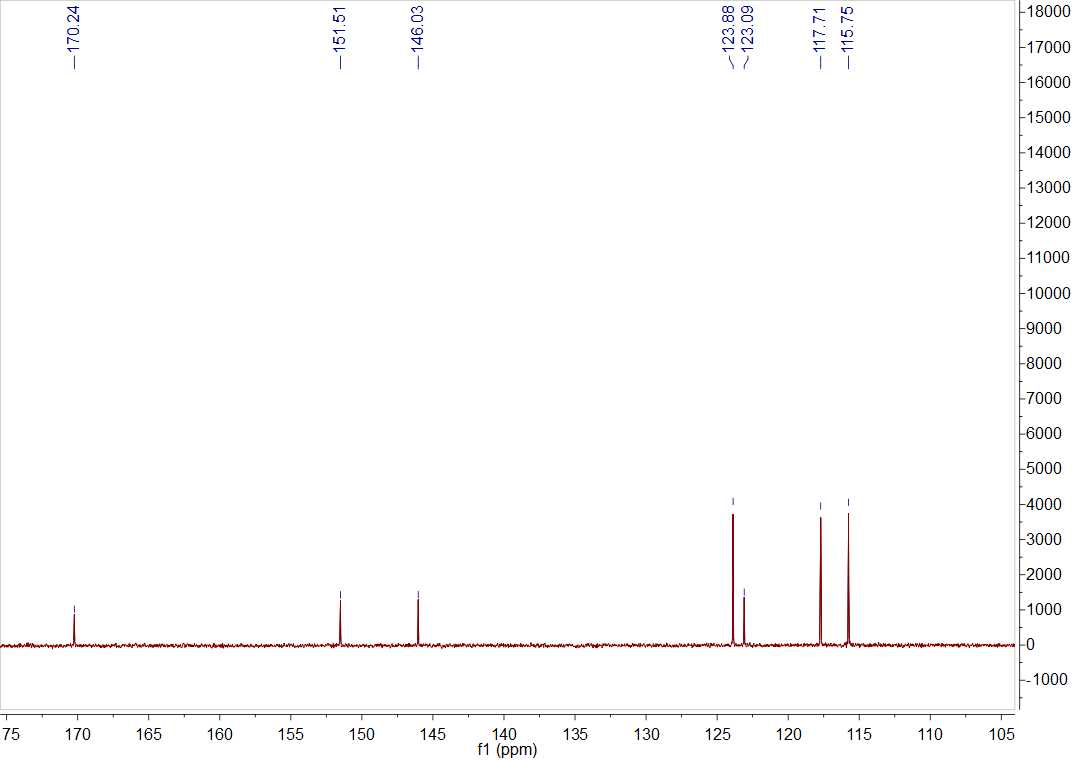


^1^H NMR spectrum (400 MHz) of compound **29** in CD_3_OD.


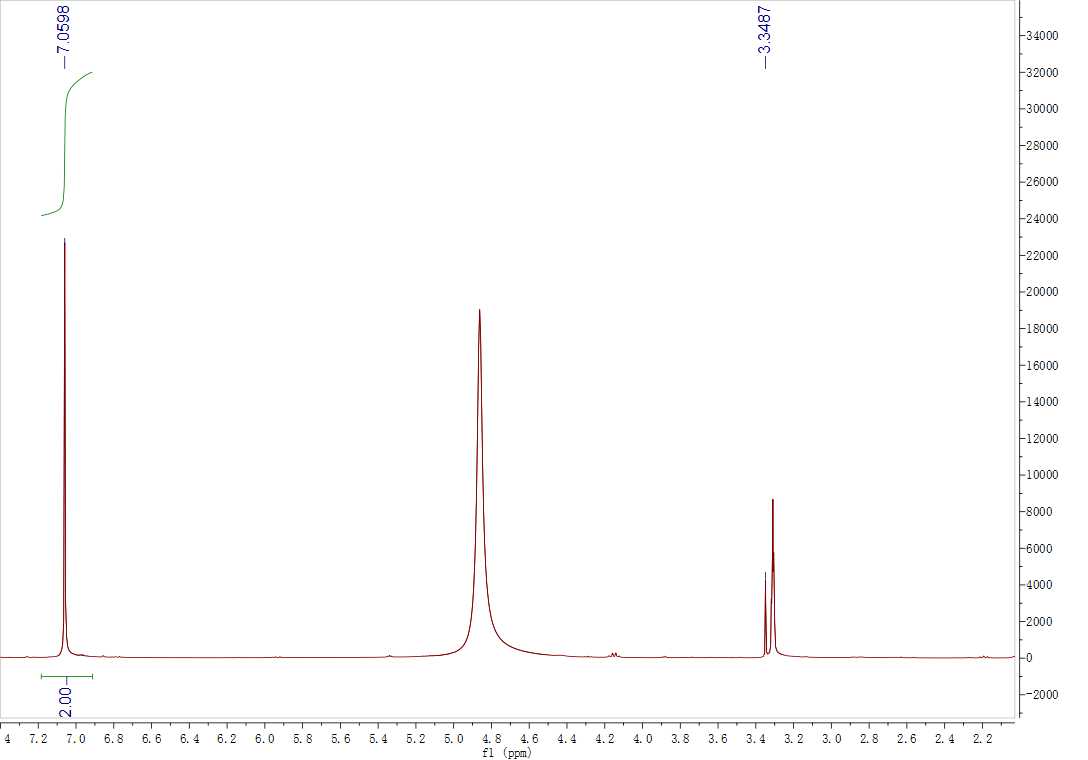


^13^C NMR spectrum (101 MHz) of compound **29** in CD_3_OD.


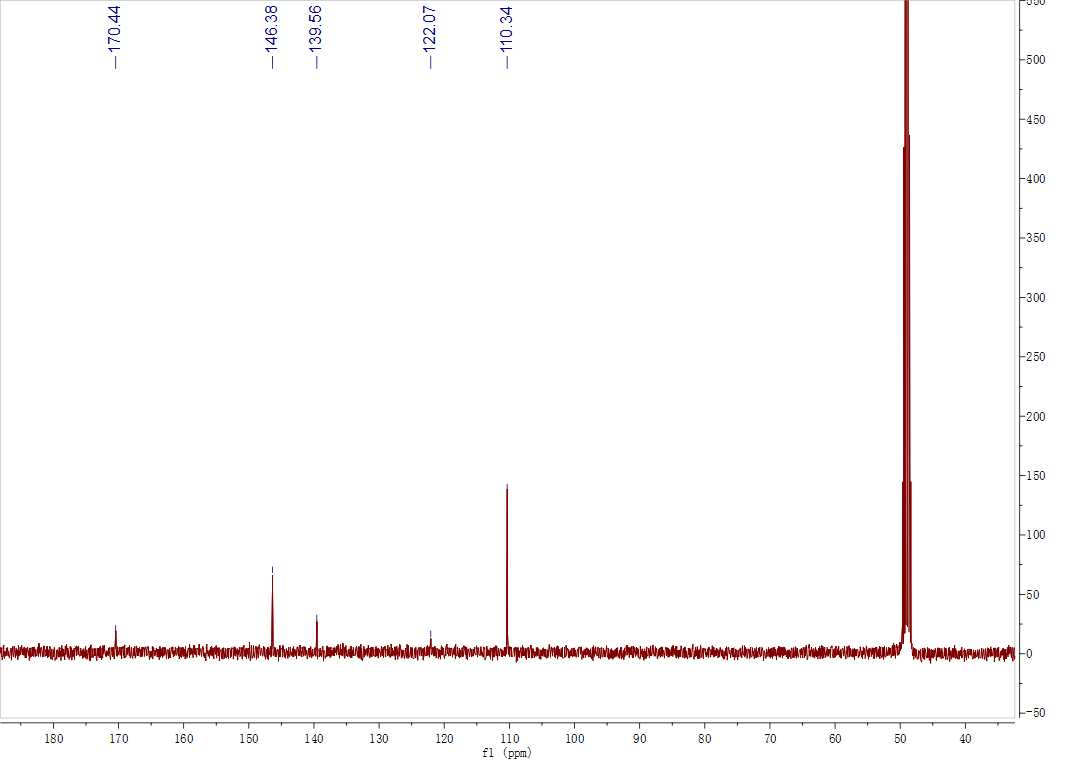


^1^H NMR spectrum (600 MHz) of compound **30** in CD_3_OD.


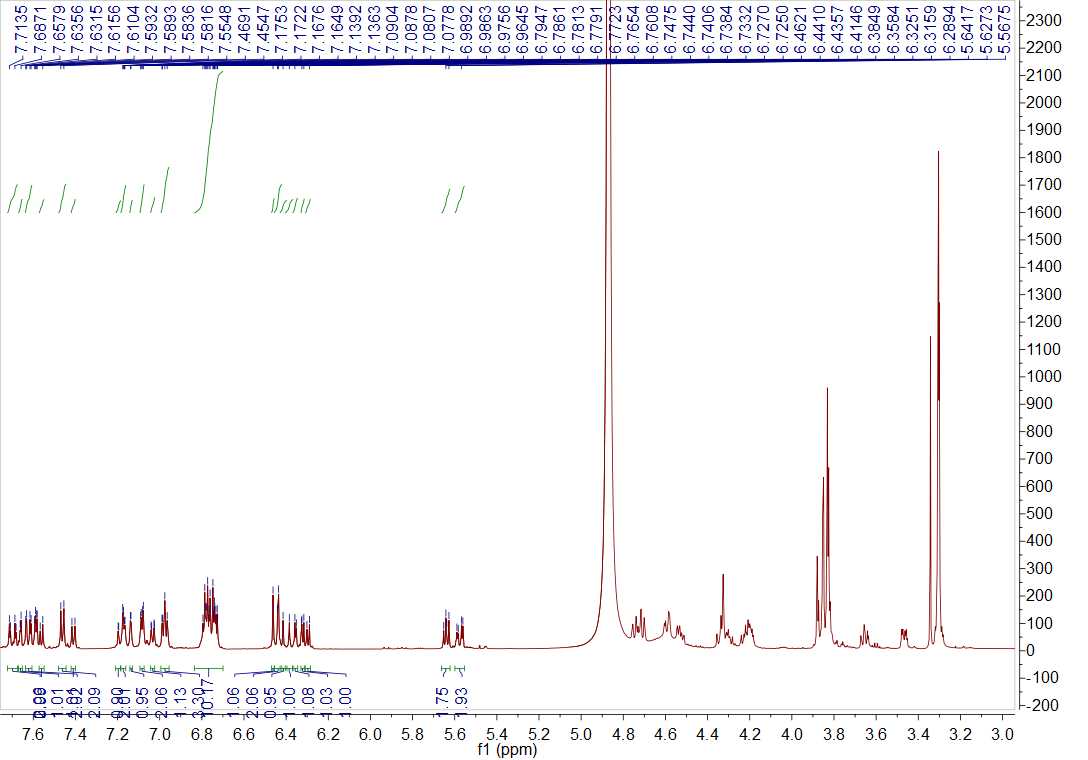


^13^C NMR spectrum (151 MHz) of compound **30** in CD_3_OD.


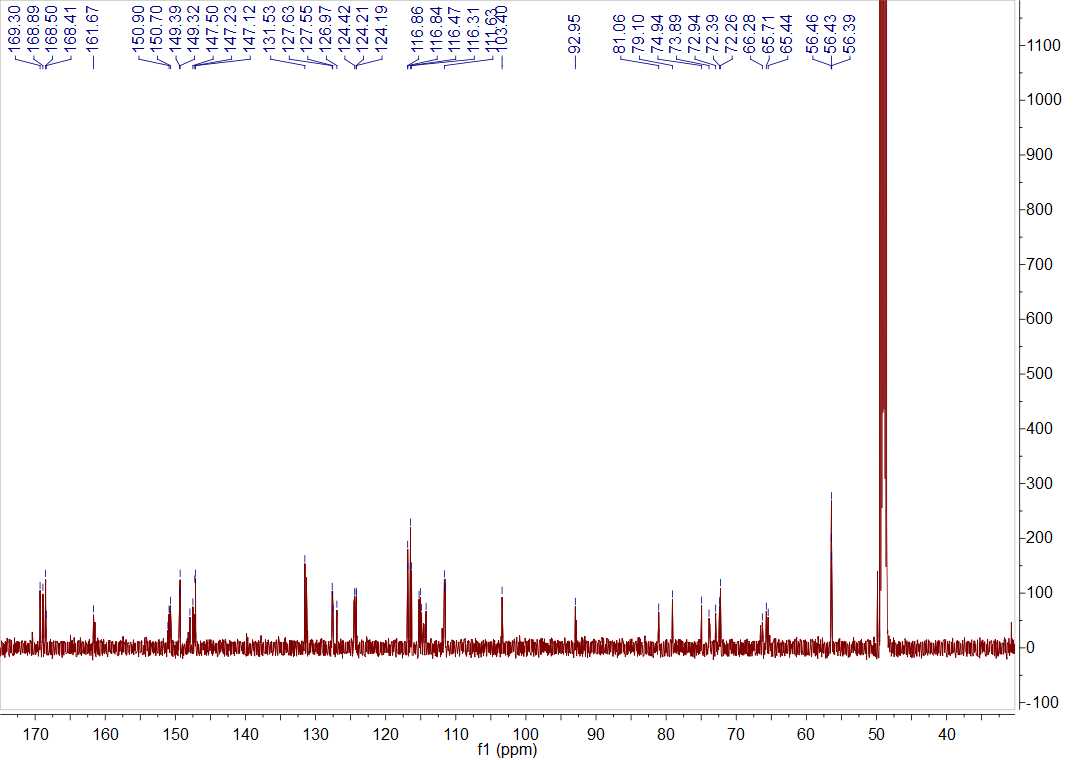


^1^H NMR spectrum (600 MHz) of compound **31** in CD_3_OD.


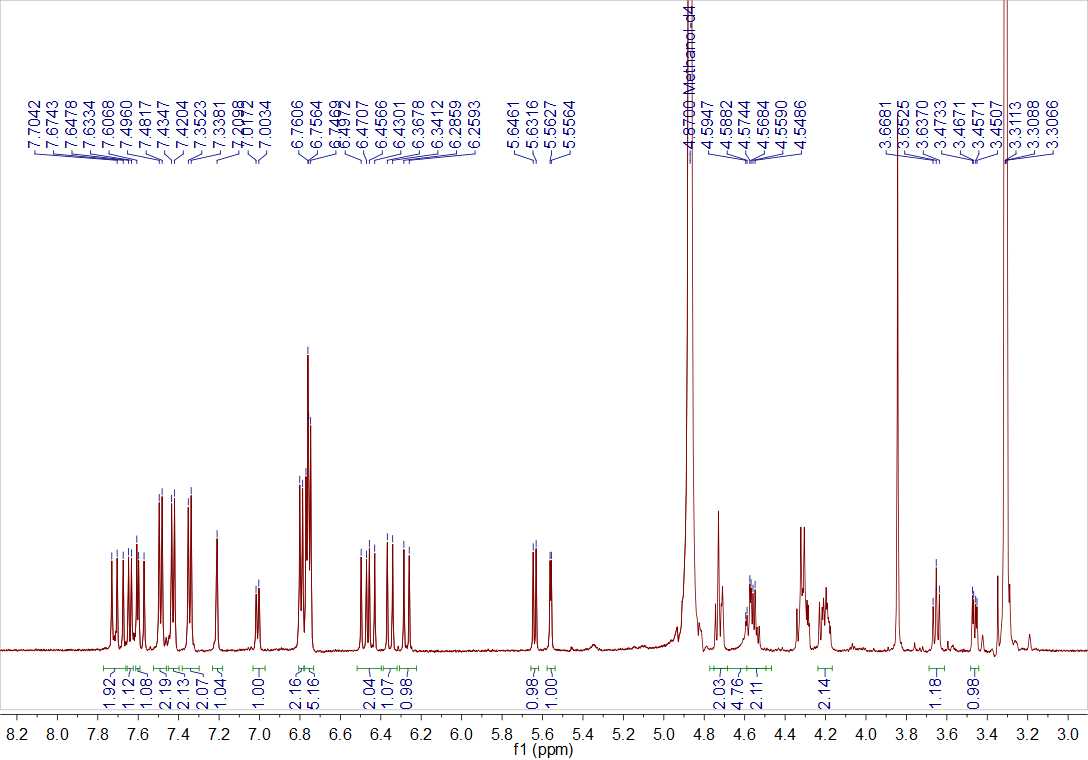


^13^C NMR spectrum (151 MHz) of compound **31** in CD_3_OD.


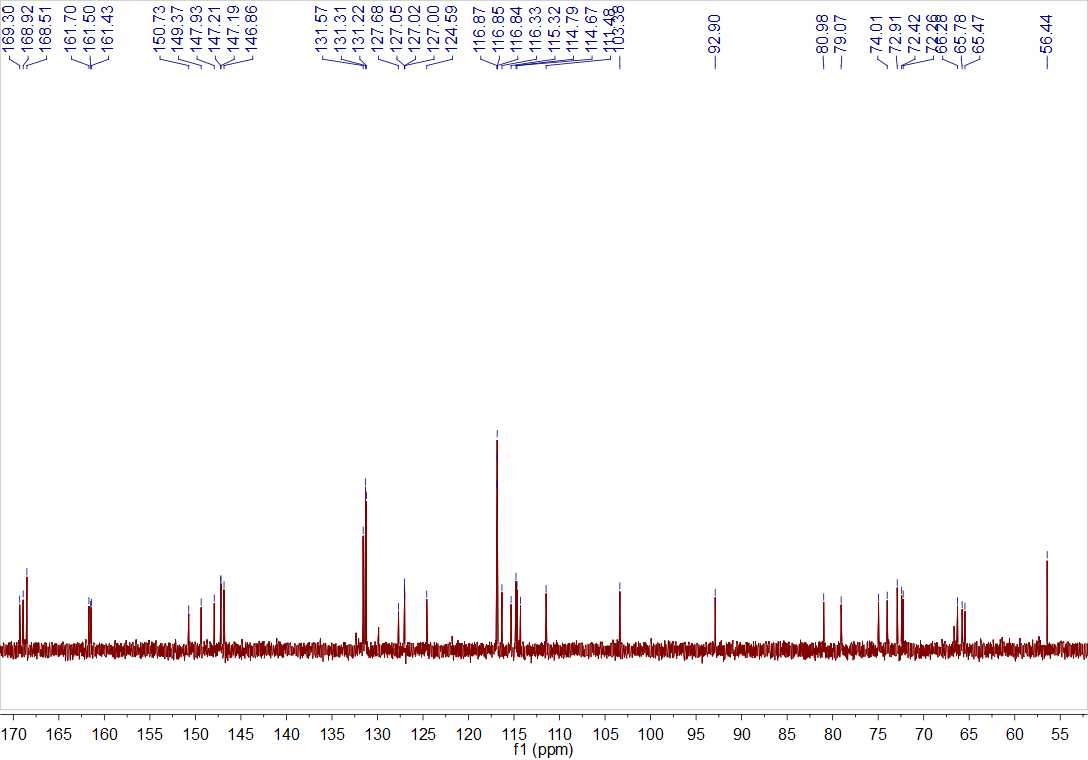


^1^H NMR spectrum (600 MHz) of compound **32** in CD_3_OD.


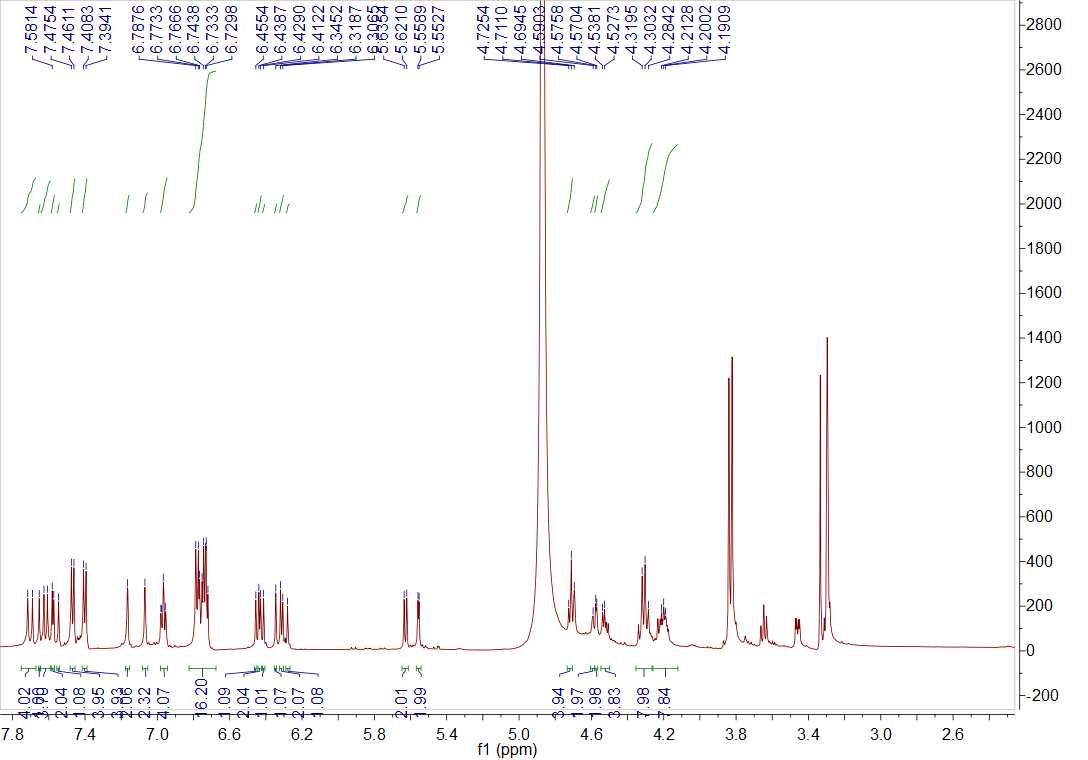


^13^C NMR spectrum (151 MHz) of compound **32** in CD_3_OD.


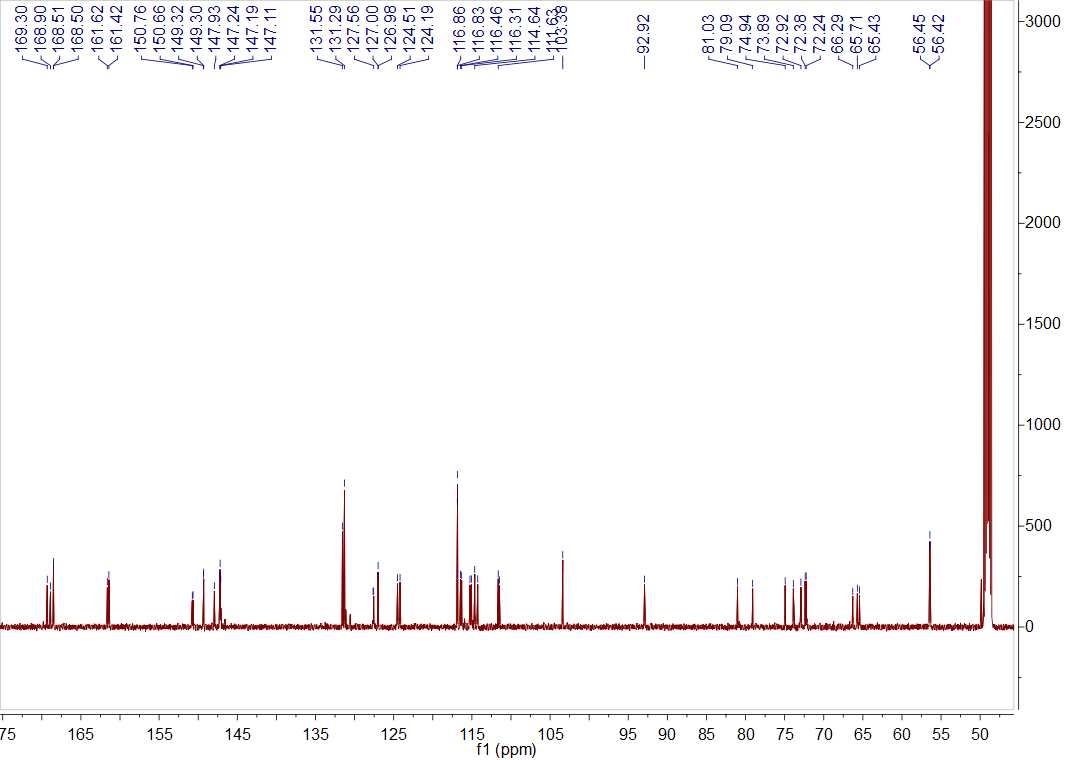


^1^H NMR spectrum (400 MHz) of compound **33** in CD_3_OD.


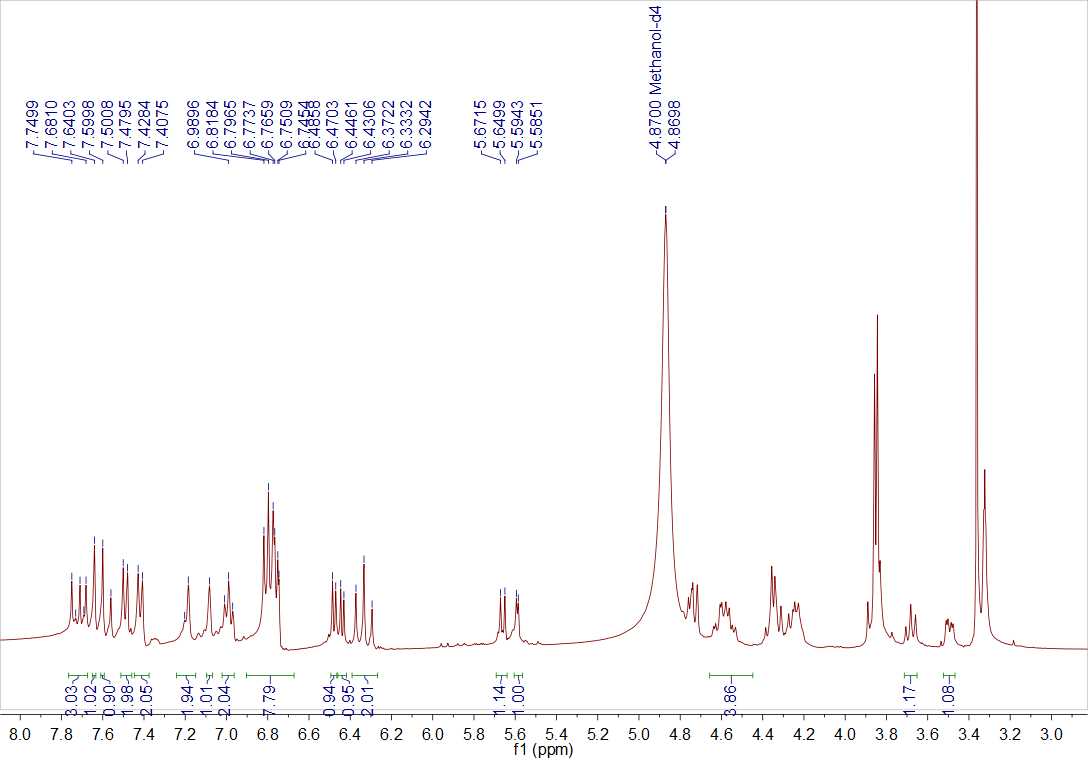


^13^C NMR spectrum (101 MHz) of compound **33** in CD_3_OD.


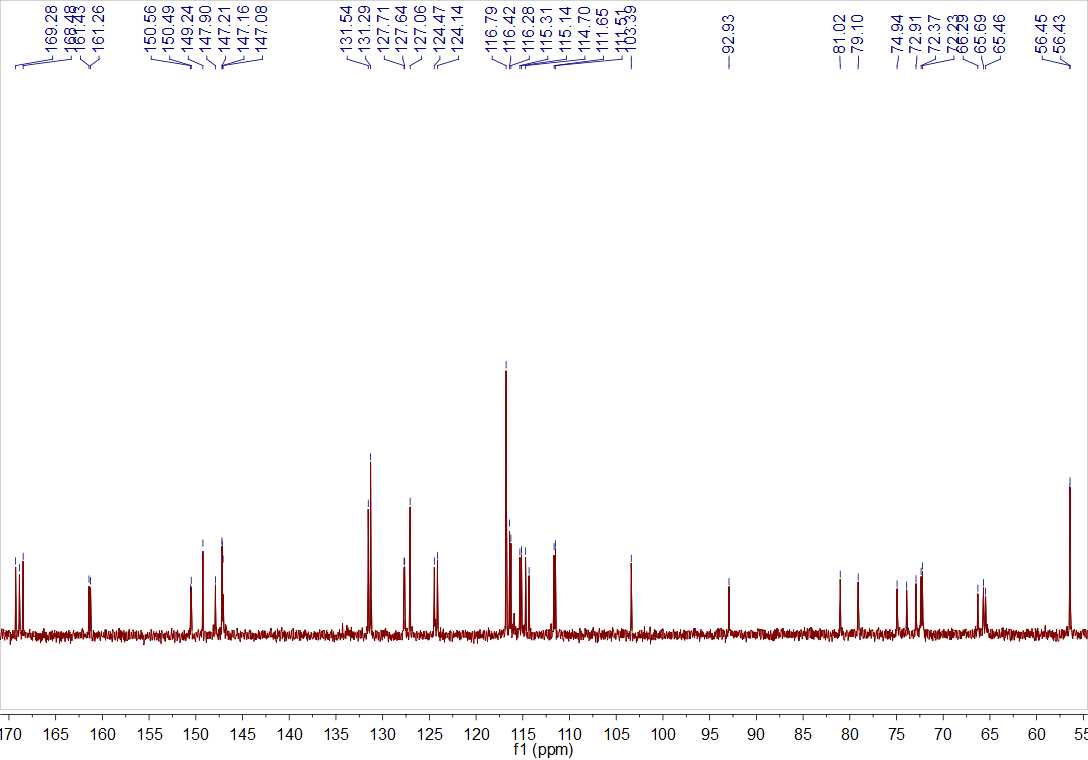


^1^H NMR spectrum (600 MHz) of compound **34** in CD_3_OD.


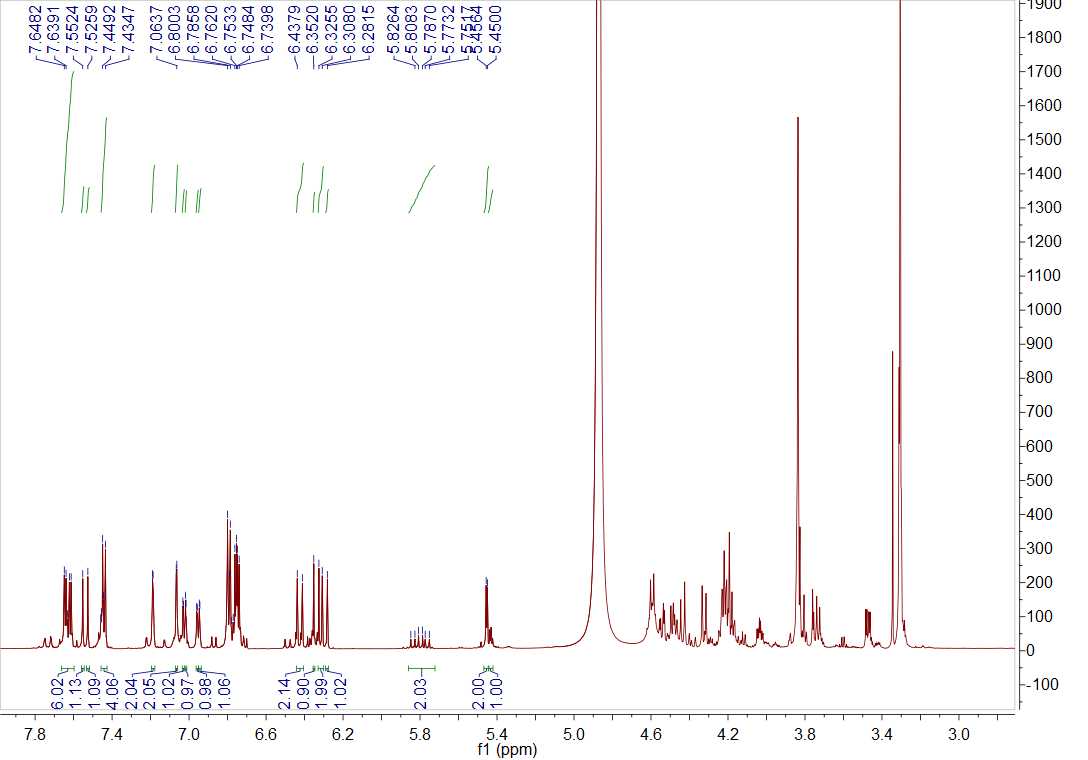


^13^C NMR spectrum (151 MHz) of compound **34** in CD_3_OD.


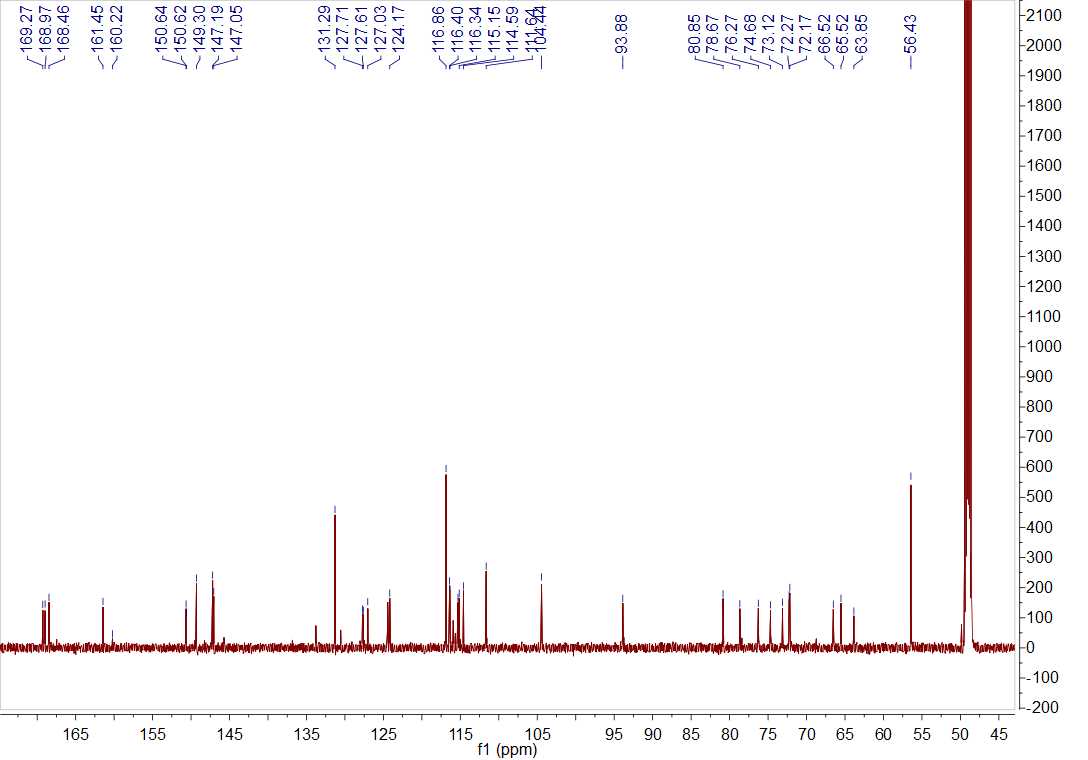

Supplement: Supplementary file 2 [file DataSheet1.docx]
